# The functional interactome of PYHIN immune regulators reveals IFIX is a sensor of viral DNA

**DOI:** 10.15252/msb.20145808

**Published:** 2015-02-09

**Authors:** Benjamin A Diner, Tuo Li, Todd M Greco, Marni S Crow, John A Fuesler, Jennifer Wang, Ileana M Cristea

**Affiliations:** Department of Molecular Biology, Lewis Thomas Laboratory, Princeton UniversityPrinceton, NJ, USA

**Keywords:** DNA sensing, IFIX, innate immunity, interactome, PYHIN

## Abstract

The human PYHIN proteins, AIM2, IFI16, IFIX, and MNDA, are critical regulators of immune response, transcription, apoptosis, and cell cycle. However, their protein interactions and underlying mechanisms remain largely uncharacterized. Here, we provide the interaction network for all PYHIN proteins and define a function in sensing of viral DNA for the previously uncharacterized IFIX protein. By designing a cell-based inducible system and integrating microscopy, immunoaffinity capture, quantitative mass spectrometry, and bioinformatics, we identify over 300 PYHIN interactions reflective of diverse functions, including DNA damage response, transcription regulation, intracellular signaling, and antiviral response. In view of the IFIX interaction with antiviral factors, including nuclear PML bodies, we further characterize IFIX and demonstrate its function in restricting herpesvirus replication. We discover that IFIX detects viral DNA in both the nucleus and cytoplasm, binding foreign DNA via its HIN domain in a sequence-non-specific manner. Furthermore, IFIX contributes to the induction of interferon response. Our results highlight the value of integrative proteomics in deducing protein function and establish IFIX as an antiviral DNA sensor important for mounting immune responses.

## Introduction

In mammalian cells, intrinsic and innate immune effectors form the first line of defense against intracellular pathogens. The human interferon-inducible PYHIN proteins, AIM2, IFI16, IFIX, and MNDA, have recently emerged as critical cellular factors in mediating both intrinsic and innate immune responses to pathogen infection and tumorigenesis. PYHIN proteins have been implicated in a wide variety of cellular processes, ranging from intracellular immune signaling, cell cycle regulation, transcription, and apoptosis (Dawson *et al*, 1995; Ding *et al*, 2004; Fujiuchi *et al*, 2004; Xin *et al*, 2004; Ludlow *et al*, 2005; Chen *et al*, 2006; Fernandes-Alnemri *et al*, 2009; Hornung *et al*, 2009; Fotouhi-Ardakani *et al*, 2010; Rathinam *et al*, 2010; Song *et al*, 2010; Unterholzner *et al*, 2010; Schattgen & Fitzgerald, 2011; Cridland *et al*, 2012; Sagulenko *et al*, 2013). The PYHIN family name derives from the shared structural domains that mediate their many functions. Each member possesses an N-terminal pyrin (PY) domain, which mediates homotypic protein–protein interactions (Park *et al*, 2007; Jin *et al*, 2013), and C-terminal HIN200 domain(s), which bind double-stranded DNA (dsDNA). Despite this functional and structural knowledge of PYHIN proteins, their mechanisms of action and interactions remain largely uncharacterized.

Recent studies from several laboratories, including ours, have established the PYHIN proteins AIM2 and IFI16 as sensors of viral dsDNA, important for eliciting immune and inflammatory responses to infection (Fernandes-Alnemri *et al*, 2009; Hornung *et al*, 2009; Rathinam *et al*, 2010; Unterholzner *et al*, 2010; Kerur *et al*, 2011; Li *et al*, 2012, 2013; Orzalli *et al*, 2012). Upon binding dsDNA, AIM2 triggers the formation of a multi-subunit complex, termed the inflammasome. This event initiates a lytic form of programmed cell death, termed ‘pyroptosis’ (Sagulenko *et al*, 2013), which propagates inflammatory signaling via release of cellular contents. Despite the reported roles of AIM2-mediated immunity in both viral and bacterial infections (Fernandes-Alnemri *et al*, 2010; Kim *et al*, 2010; Rathinam *et al*, 2010; Wu *et al*, 2010; Ge *et al*, 2012; Reinholz *et al*, 2013), the factors regulating AIM2-mediated cell death remain poorly understood. IFI16 has also been established as a critical factor in immune response. After binding viral dsDNA in the cytoplasm, IFI16 induces robust expression of interferon-β (IFN-β) in differentiated THP-1 monocytes (Unterholzner *et al*, 2010). We and others have recently demonstrated, however, that IFI16 functions in the nucleus to sense nuclear-replicating dsDNA herpesviruses in primary human fibroblasts (Orzalli *et al*, 2012; Li *et al*, 2013), challenging the long-withstanding belief that sensing occurs exclusively in the cytosol. Additional evidence has suggested that, like AIM2, IFI16 can also initiate inflammasome formation in response to herpesvirus infection (Kerur *et al*, 2011; Ansari *et al*, 2013; Johnson *et al*, 2013; Singh *et al*, 2013). Altogether, these data implicate AIM2 and IFI16 as critical antimicrobial factors in mammalian cells.

Despite a high degree of sequence similarity within the PYHIN family (Ludlow *et al*, 2005; Cridland *et al*, 2012), little is understood regarding the functions of the other two human PYHIN proteins—IFIX and MNDA. IFIX is thought to act as a tumor suppressor through indirect stabilization of p53. Tumor xenograft studies in mice have demonstrated that ectopic IFIX expression drastically reduces tumorigenicity (Ding *et al*, 2004, 2006). Similar tumor suppressive activities through p53- and NF-κB-dependent pathways have been suggested for IFI16 and AIM2 (Chen *et al*, 2006; Song *et al*, 2008; Kondo *et al*, 2012). Additionally, expression of certain PYHIN proteins is lost or abnormally low in a variety of human cancers (Fujiuchi *et al*, 2004; Xin *et al*, 2004; Liao *et al*, 2010; Lee *et al*, 2012). Although MNDA is the founding member of the family, this protein still remains largely uncharacterized. MNDA acts as a transcriptional regulator and is known to be critical in cellular differentiation (Dawson *et al*, 1995; Briggs *et al*, 2005; Ludlow *et al*, 2005). Dysregulation of MNDA-dependent apoptotic processes in neutrophils is thought to contribute to sepsis-associated hyper-inflammation (Fotouhi-Ardakani *et al*, 2010). More recently, we demonstrated that the PY domains of IFIX and MNDA, as well as IFI16, are targeted by the human cytomegalovirus (HCMV) protein pUL83, which prevents their oligomerization within the nucleus (Li *et al*, 2013). As our analyses revealed that pUL83 abates IFI16-dependent immune signaling during HCMV infection, these results suggest that IFIX and MNDA may also have intrinsic antiviral roles in the cell.

Here, to gain mechanistic insights into their functions, we designed a cell-based system for the inducible expression, affinity capture, and functional assaying of all four human PYHIN proteins—AIM2, IFI16, IFIX, and MNDA. Integrating fluorescence microscopy, immunoaffinity purifications, quantitative mass spectrometry, and bioinformatics analyses, we built the first global protein interactome of the PYHIN family. We identified over 300 PYHIN interacting proteins with roles in intracellular signaling, cell cycle control, DNA damage response, apoptosis, transcriptional regulation, and ribosome biogenesis. Altogether, these interactions provide insight into the mechanisms by which the PYHIN family executes their purported tumor suppressor and immune regulatory functions. Focusing on the least characterized PYHIN protein IFIX, we utilized orthogonal molecular virology and biochemical methods to demonstrate its interaction with components of a critical antiviral defense pathway against dsDNA viruses—PML nuclear bodies. We show that, similar to PML body components, IFIX exerts an antiviral effect to limit herpesvirus replication. Importantly, we establish IFIX as a previously unrecognized sensor of intracellular viral dsDNA. We determine the domain that mediates its DNA binding capabilities and prove the requirement of IFIX for initiating innate immune programs. Finally, we demonstrate IFIX binding to viral DNA in the nucleus during herpesvirus infection. Our studies underscore the value of integrative proteomics in deducing protein function based on interaction networks, as well as characterizing an important immune function mediated by the human PYHIN protein IFIX.

## Results

### Construction of a cell-based system to characterize the interactome of PYHIN proteins

To elucidate PYHIN protein interactions, we generated a series of cell lines that inducibly express each PYHIN protein tagged with a fluorescent tag for visualization, affinity isolation, and subsequent proteomic analyses. This was necessary given the lack of appropriate antibodies for immunoaffinity purification of all four PYHIN proteins. We selected HEK293 cells as a model system, as these cells are commonly used for characterizing fundamental molecular and cellular processes, such as transcription, cell cycle, and apoptosis, and are highly suited for transgene expression. Previous studies have used HEK293T cells to reconstitute immune signaling pathways by reintroducing specific pathway components, which are normally absent in these cells. (Ablasser *et al*, 2013a,b, 2014; Diner *et al*, 2013; Sun *et al*, 2013). Therefore, we instead used a system derived from HEK293 cells, in which we could detect endogenous STING and IRF3 (Supplementary Fig S1B), two central components of DNA stimulated immune signaling pathway (Tanaka & Chen, 2012). This is in agreement with previous reports that HEK293 cells already express STING (Ishikawa & Barber, 2008) and do not require its reintroduction for virus-triggered interferon response (Li *et al*, 2012), as in the case of HEK293T cells. Additionally, we observed that the *pyhin* genes, although present at low levels, are inducible by type I interferon in wild-type HEK293 cells (Supplementary Fig S1A). We therefore generated tetracycline-inducible HEK293 cell lines that express integrated PYHIN transgenes, allowing transient overexpression of the four PYHIN proteins. This transient overexpression avoided the potential PYHIN-mediated cell cycle arrest, apoptosis, and transcriptional changes that may be associated with this family of proteins (Ding *et al*, 2004; Fujiuchi *et al*, 2004; Chen *et al*, 2006; Sun *et al*, 2014). As IFI16 and IFIX have multiple isoforms, we used transgenes encoding the longest and most abundantly expressed isoforms for each—IFI16B and IFIXα1 (Ding *et al*, 2004; Li *et al*, 2012). GFP was chosen as the affinity tag to ensure efficient isolation of PYHIN proteins using high-affinity anti-GFP antibodies, as we have previously described (Cristea *et al*, 2005; Joshi *et al*, 2013). PYHIN proteins were tagged at either the N- or C-termini to eliminate tag-dependent effects on protein localization and interactions, providing a total of nine HEK293 cell lines inducibly expressing one of the GFP-tagged PYHIN proteins or GFP alone as control (FigA and B, Supplementary Fig S2A). While some variations in expression levels were observed, all tagged PYHIN proteins were expressed well and at levels comparable to the control GFP (Supplementary Fig S2B).

**Figure 1:**
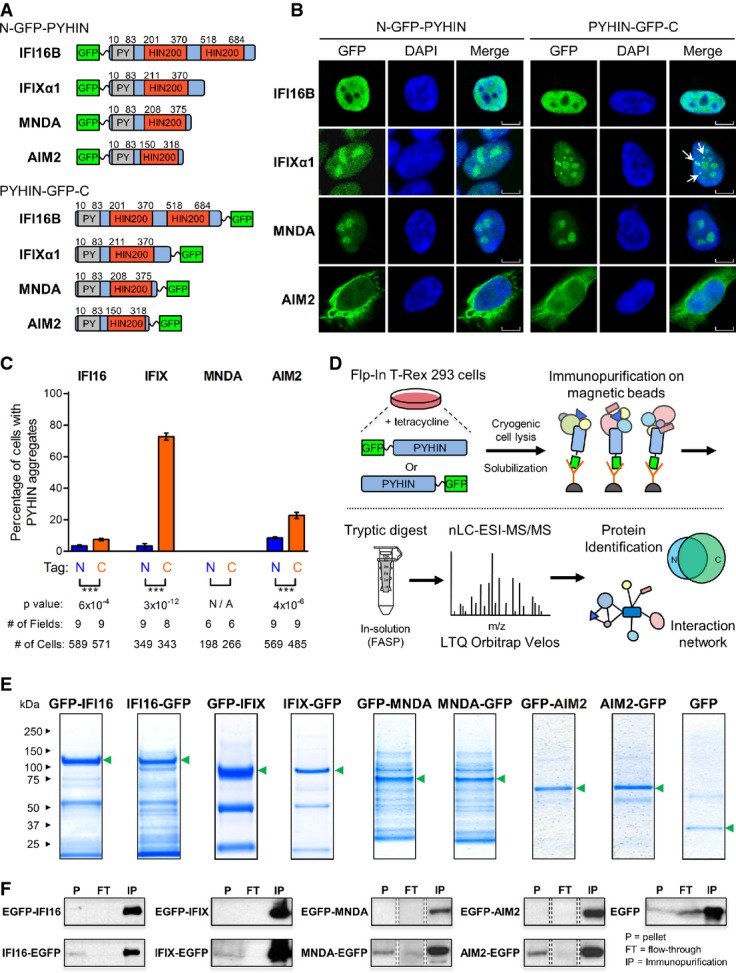
Construction of a cell-based system for elucidating the PYHIN family protein interaction network A PYHIN proteins were tagged with GFP at either the N-terminus, adjacent to the pyrin domain (gray), or the C-terminus, adjacent to the HIN200 domain (red).

B The localization of all eight PYHIN-GFP constructs within their respective inducible HEK293 cell lines was visualized by fluorescence confocal microscopy. Punctate structures are indicated by white arrows. Scale bars, 5 μm.

C The percentages of cells displaying PYHIN-GFP punctate structures were determined for all PYHIN-GFP HEK293 cell lines using fluorescence confocal microscopy. The number of cells and fields of view are indicated. Mean values ± SEM (*n* varies). ***P ≤ 0.001, comparing N- and C-terminally tagged constructs (Student's unpaired *t*-test; two-tailed).

D Proteomic workflow for mapping the protein interaction network of GFP-tagged PYHIN proteins. Specificity of PYHIN–prey interactions was assessed by label-free AP-MS and SAINT.

E PYHIN-GFP and GFP immunoisolates were resolved by SDS–PAGE and Coomassie staining. PYHIN-GFP baits are indicated (green arrows).

F Pellet (P), flow-through (FT), and immunopurified (IP) fractions from PYHIN-GFP immunoisolations were assessed by Western blotting to determine isolation efficiency of PYHIN targets. Protein bands represent 1% loading of each indicated fraction.

The localization of the GFP-tagged PYHIN proteins was assessed by immunofluorescence confocal microscopy. While the control cell line had a uniform distribution of GFP staining (Supplementary Fig S2A), all four PYHIN proteins exhibited appropriate sub-cellular distributions (Fig1B). IFI16, IFIX, and MNDA displayed nuclear localization, consistent with possessing functional nuclear localization signals (NLS) (Duhl *et al*, 1989; Ding *et al*, 2004; Li *et al*, 2012). In addition to localization within the nucleoplasm, IFIX and MNDA displayed prominent staining within nucleoli. In contrast, both GFP-tagged AIM2 constructs displayed a diffuse localization that predominated in the cytoplasm, as previously observed (Hornung *et al*, 2009). Although AIM2 lacks an NLS, its nuclear localization has been reported (Cresswell *et al*, 2005). Interestingly, we observed additional punctate staining enriched in the C-terminally tagged IFI16, IFIX, and AIM2 constructs (Fig1B and C and Supplementary Fig S2C). The most striking punctate formation was observed for IFIX, for which almost 75% of cells expressing C-terminally tagged IFIX displayed nuclear punctate structures, compared to the approximately 3% of cells expressing the N-terminally tagged IFIX (*n* > 300) (Fig1C). IFIX puncta were dispersed exclusively within nuclei (Supplementary Fig S2C). While IFIX-containing punctate structures have not been reported for the alpha isoform, a previous study utilizing N-terminally GFP-tagged IFIX showed similar puncta formation for the IFIX γ1 isoform (Ding *et al*, 2004). In contrast to IFIX, IFI16, and AIM2, we did not observe punctate structures for either MNDA constructs. Because the N-terminal PY domain mediates cooperative self-assembly of PYHIN proteins (Li *et al*, 2013; Morrone *et al*, 2014), the observed tag-dependent behavior may be due to an alteration in PY domain structure. The PY domain is thought to be maintained in an inaccessible, auto-inhibited state by the HIN200 domain and to become accessible upon the HIN200 domain binding to dsDNA, allowing oligomerization via intermolecular PY–PY interactions (Jin *et al*, 2012, 2013). Thus, MNDA's inability to form puncta may reflect structural differences relative to the other PYHIN proteins. This is in agreement with our recent report that the PY domains of IFI16 and IFIX can oligomerize within the nucleus, while the MNDA PY domain maintains a predominantly diffuse localization (Li *et al*, 2013). Overall, this differential tagging allowed us to focus on the most consistent interactions that were not affected by the tag location. This was particularly important given the scarcity of prior knowledge regarding PYHIN protein interactions and therefore the expected challenge in extrapolating key interactions for validation and functional studies.

### Efficient immunoaffinity purification of N- and C-terminus GFP-tagged PYHIN proteins

To build their interaction network, the GFP-tagged PYHIN proteins were inducibly expressed and immunoaffinity-purified (IP) (Fig1D) using a recently described approach (Joshi *et al*, 2013). The lysis buffer conditions were optimized for efficient isolation of the PYHIN baits with their interacting protein complexes, while minimizing non-specific associations. The isolations were supplemented with DNase I to limit the abundance of indirect DNA-mediated interactions. All PYHIN baits were observed as the most prominent bands within their respective SDS–PAGE-resolved immunoisolates (Fig1E). Furthermore, in all PYHIN IPs, the majority of the bait proteins were isolated (‘IP’ fractions), with minor amounts left in the flow-through (‘FT’) fractions (Fig1F; full blots shown in Supplementary Fig S3A and B). As expected, the negative control GFP IP was relatively clean, assuring that our lysis and isolation conditions minimized non-specific interactions. Overall, these analyses demonstrate adequate optimization of our IPs and efficient isolations of all PYHIN proteins. In terms of differences between the isolations of N- and C-terminally tagged constructs, while N-terminally tagged PYHIN baits were not present in the pellet (‘P’) fractions, C-terminally tagged baits were evident at low levels (Fig1F). This insoluble PYHIN protein may partly correspond to the tag-dependent aggregates observed by microscopy (Fig1C and Supplementary Fig S2C).

**Figure 2:**
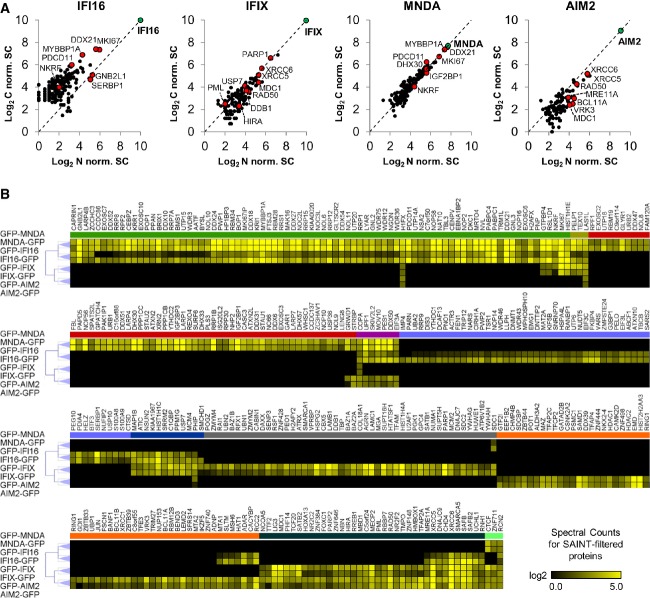
Label-free quantification analysis of PYHIN family interactions A Spectral counts of SAINT-filtered prey proteins from N- and C-terminally tagged PYHIN isolations were normalized by the spectral abundance of their respective baits, log_2_-transformed, and scatter-plotted. Spectral counts were averaged across at least two biological replicates. PYHIN baits are labeled in green, while interactions of interest are labeled in red.

To identify interactions, the proteins co-isolated with the tagged baits were digested using trypsin and analyzed by mass spectrometry using a label-free quantification approach. Non-specific interactions were filtered out via ‘significance analysis of interactome’ (SAINT) probabilistic scoring (Choi *et al*, 2011). Given the limited knowledge regarding validated PYHIN protein interactions, we assessed the distribution of SAINT probability scores. We optimized the selection of SAINT thresholds to achieve a balance between specificity (for reducing false positives) and sensitivity (for maximizing the identification of candidate interactions) (Supplementary Fig S4). As a result, we selected an average confidence score threshold of ≥ 0.85 for IFI16, IFIX, and AIM2. In comparison with the other PYHIN proteins, MNDA had the highest number of co-isolated proteins (Fig1E). Thus, to maximize the selection of the most prominent and likely specific interactions, we used a more stringent average SAINT score of ≥ 0.95 for MNDA. Importantly, all candidate interactions passing these thresholds were identified in both biological replicates. Relative abundances of SAINT-filtered interactions were next assessed by averaging prey spectral counts across biological replicates and then normalizing to the spectral abundances of appropriate PYHIN baits. Given their variation in cellular distributions, we first compared the interactions of N- and C-terminally tagged baits (Fig2A). Notably, for IFIX, MNDA, and AIM2, interactions were not significantly biased for either N- or C-terminal tagged baits. Therefore, the majority of these observed interactions were not impacted by tagging. For IFIX, this suggests that the observed interactions are mainly representative of its nucleoplasmic and nucleolar localizations, and less representative of its punctate localization (a subset likely partly lost in the pellet fraction, Fig1F). In contrast to the other PYHIN proteins, IFI16 displayed a higher abundance of protein interactions with C-terminally tagged protein versus its N-terminal counterpart (Figs1E and 2A). It is possible that, although not triggering a change in localization, the tagging at the N-terminus disrupts some of its PY-mediated interactions. Alternatively, this bias may be associated with the greater expression of the N-terminally tagged IFI16, skewing the label-free quantification following normalization to bait amounts across different isolations. Highlighted in red are several prominent interactions detected with both N- and C-terminally tagged PYHIN proteins (Fig2A). For IFI16 and MNDA, these interactions included regulators of NF-κB (PDCD11 and NKRF) and c-Jun (MYBBP1A and DDX21) and antiviral helicases MOV10 and DHX30; for IFIX and AIM2, abundant interactions included proteins involved in DNA damage response (e.g., XRCC6/XRCC5, MRE11A, RAD50, MDC1, and USP7).

B Hierarchical clustering of all 355 SAINT-filtered PYHIN–prey interactions identified by label-free AP-MS as a function of their log_2_-transformed spectral counts averaged across biological replicates. Clustering is based on Pearson's correlation and complete linkage between both N- and C-terminally GFP-tagged PYHIN immunoisolations. Colored clusters illustrate the following groupings of SAINT-filtered interactions with indicated PYHIN proteins: green: IFI16, MNDA; gold: IFI16, MNDA, IFIX; red: MNDA; violet: IFI16, MNDA, AIM2; light blue: IFI16; dark blue: IFI16, IFIX; gray: IFI1X; orange: AIM2; dark green: IFIX, AIM2; mint: IFIX, MNDA, AIM2.

### Protein interaction clustering reflects unique and common functions among PYHIN proteins

To extrapolate unique or shared molecular functions within the PYHIN family, we next used hierarchical clustering to group the proteins predicted to be specific for each PYHIN protein (Fig2B). All SAINT-filtered interactions were clustered according to their spectral counts. As expected, for each PYHIN protein, N- and C-terminally tagged counterparts displayed the largest degree of similarity between their interaction profiles. Across PYHIN proteins, the most closely related interaction profiles existed between IFI16 and MNDA, suggesting functional similarities. This cluster of interactions (Fig2B, green cluster) was enriched in proteins functioning in rRNA processing and ribosome biogenesis. Clusters of common preys were also observed for IFIX and AIM2, IFIX and IFI16, as well as for three or all four PYHIN proteins. IFIX and AIM2 common interaction profiles (dark green cluster) contained proteins involved in dsDNA repair, telomere maintenance, and chromatin remodeling. Clustering also provided a clear representation of interactions unique to a given PYHIN protein. For example, the uncharacterized protein IFIX was found to be uniquely associated with proteins involved in apoptosis, antiviral defense, and regulation of chromosomal architecture and transcription (Fig2B gray cluster). Altogether, the clustering of all protein interactions observed from our AP-MS studies allowed us to assess the relatedness of PYHIN family functions.

### The functional PYHIN protein interaction network

**Figure 3:**
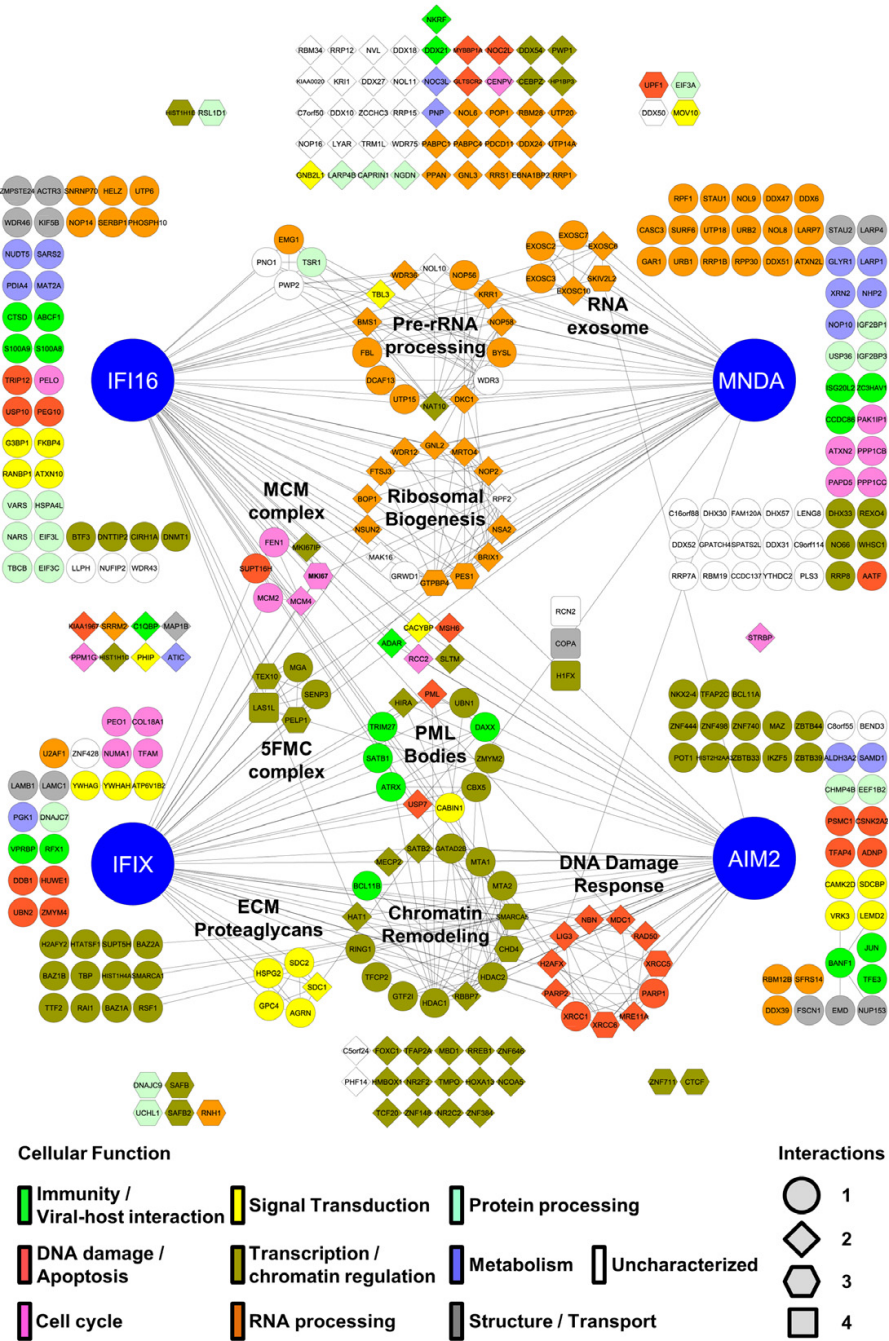
The interaction network of the PYHIN protein family 345 SAINT-filtered PYHIN–prey protein interactions, common between N- and C-terminally tagged PYHIN isolations, were assessed using the STRING database and visualized in Cytoscape. GO terms were assigned to individual prey nodes and color-coded based on assigned molecular functions (key, bottom left). The number of PYHIN proteins found to interact with a particular prey is represented by the prey node shape (key, bottom right). Known complexes are annotated and network edges indicate interactions with either a PYHIN protein or other prey proteins. Edgeless nodes are arranged in clusters closest to the PYHIN protein(s) with which they interact.

To integrate the putative PYHIN interactions within a network and assess functional complex enrichment, we next searched the SAINT-filtered interactions from all PYHIN isolations against the STRING database of known protein association networks (Szklarczyk *et al*, 2011). Given the scarcity of information regarding PYHIN interactions and to avoid variations from the tag location, we chose to construct the network by illustrating interactions present with both N- and C-terminally tagged PYHIN proteins. These reflected the majority (345 out of 355) of the detected interactions (Supplementary Tables S4, S5 and S6). Individual PYHIN interaction networks were merged via Cytoscape (Shannon *et al*, 2003), and gene ontology (GO) terms were assigned to all interactions (Fig3 and Supplementary File S1). The relative enrichment of the proteins isolated with each bait was estimated by calculating a normalized spectral abundance factor (NSAF) for each protein (Zybailov *et al*, 2007), which was then normalized to the estimated abundance of the protein within the human proteome (PAX values) (Weiss *et al*, 2010), as we previously reported (Tsai *et al*, 2012). This analysis highlighted the most enriched SAINT-filtered interactions within the isolation of each PYHIN protein (Supplementary Figs S5, S6, S7 and S8). Interestingly, several prominent functional protein complexes were evident in the PYHIN network (Fig3). Consistent with our hierarchical clustering data, complexes involved in ribosome biogenesis and RNA processing were enriched within IFI16 and MNDA shared networks, whereas IFIX and AIM2 associated with distinct complexes related to antiviral responses, DNA damage responses (DDR), and chromatin remodeling.

In addition to identifying numerous interactions likely involved in the housekeeping functions of PYHIN proteins, we observed associations possibly reflective of the intrinsic and innate immune roles of the PYHIN proteins. For the least characterized PYHIN protein, IFIX, we observed enriched association with an interaction cluster formed by components of promyelocytic leukemia (PML) nuclear bodies. PML bodies are dynamic assemblies of proteins that form distinct puncta in the nuclear matrix and have been implicated in DNA repair processes and intrinsic immunity (Xu *et al*, 2003; Lallemand-Breitenbach & de The, 2010; Cuchet-Lourenco *et al*, 2013; Glass & Everett, 2013). Associations with proteins involved in immune processes, signal transduction, and apoptosis were also observed for the other PYHIN proteins. Key regulators of tumor suppressor p53 activity USP10 and TRIP12 and anti-apoptotic protein PEG10 were enriched in IFI16 isolations. Additionally, GLTSCR2, a nucleolar stabilizer of p53 during ribosomal stress and a proposed tumor suppressor, was specific in both IFI16 and MNDA networks. As IFI16 has established roles in regulating p53-dependent transcription and apoptosis (Aglipay *et al*, 2003; Fujiuchi *et al*, 2004), these interactions may underlie the mechanisms through which it accomplishes such functions. Furthermore, we observed IFI16 interactions with proteins involved in eliciting immune and pro-inflammatory responses, such as the ATP-binding cassette family protein ABCF1. ABCF1 was recently reported to interact with a murine PYHIN protein p204 (Lee *et al*, 2013), which, similar to IFI16, regulates cytokine induction in response to dsDNA (Unterholzner *et al*, 2010). IFI16 and MNDA also interacted with the negative regulator of NF-κB-mediated gene expression, NKRF. Overall, these interactions may be important in propagating immune signals upon IFI16-dependent DNA sensing. Similar to IFI16, we observed several AIM2 interactions with roles in signal transduction. For instance, regulator of ERK signaling VRK3, interferon-inducible interleukin-5 receptor adapter molecule SDCBP, and casein kinase II subunit CSNK2A2 were uniquely associated with AIM2. This is particularly intriguing as casein kinase II is known to modulate transcriptional factors mediating immune and apoptotic responses, such as NF-κB, STAT1, IRF1, IRF2, and c-JUN. In fact, we additionally found c-JUN to uniquely interact with AIM2 within the PYHIN family. As c-JUN is known to modulate cellular processes involved in immunity, including expression of antiviral cytokines, this association may provide a new mechanistic perspective of AIM2-dependent immune functions.

In addition to the PYHIN interactions related to immune processes, we identified numerous associations indicative of functional versatility for this protein family. IFIX, for instance, was enriched in interactions involved in transcriptional regulation and chromatin modulation. These interactions included nearly all of the components of the Five Friends of Methylated Chromatin (5FMC) complex (TEX10, SENP3, LAS1L, and PELP1). This complex is involved in desumoylation of target transcription factors, such as ZNF148, which we also observed as a specific interaction with IFIX and AIM2 (Fanis *et al*, 2012). One subset of interactions shared between IFI16 and MNDA were the members of the RNA exosome complex. These included structural components EXOSC2, EXOSC3, EXOSC6, and EXOSC7, RNA helicase SKIV2l2, and RNA nucleases EXOSC10 and DIS3. The RNA exosome is responsible for the degradation of all classes of RNAs in both nuclear and cytoplasmic compartments (Chlebowski *et al*, 2013). Of interest, the RNA exosome specifically degrades mRNAs containing AU-rich elements (AREs), typically found in the 3′ untranslated regions of short-lived mRNAs encoding cytokines and inflammatory proteins (Stoecklin *et al*, 2006; Murray & Schoenberg, 2007). Another example of PYHIN functional versatility is the association shared by AIM2 and IFIX with proteins involved in DNA damage responses (DDR), including the XRCC6/Ku70-XRCC5/Ku80 complex and the MRE11A-RAD50-NBN (MRN) complex with MDC1. The association with the XRCC6/XRCC5 complex was also observed for IFI16. Additionally, IFI16 and MNDA specifically interacted with the histone variant H2AFX, known to be required for recruitment of DDR pathway components and p53-mediated cell cycle arrest following genotoxic stress (Paull *et al*, 2000). This may indicate that all four PYHIN proteins function at different levels within the DDR pathway.

### Identification of core IFI16 interactions in fibroblasts and monocytes

**Figure 4:**
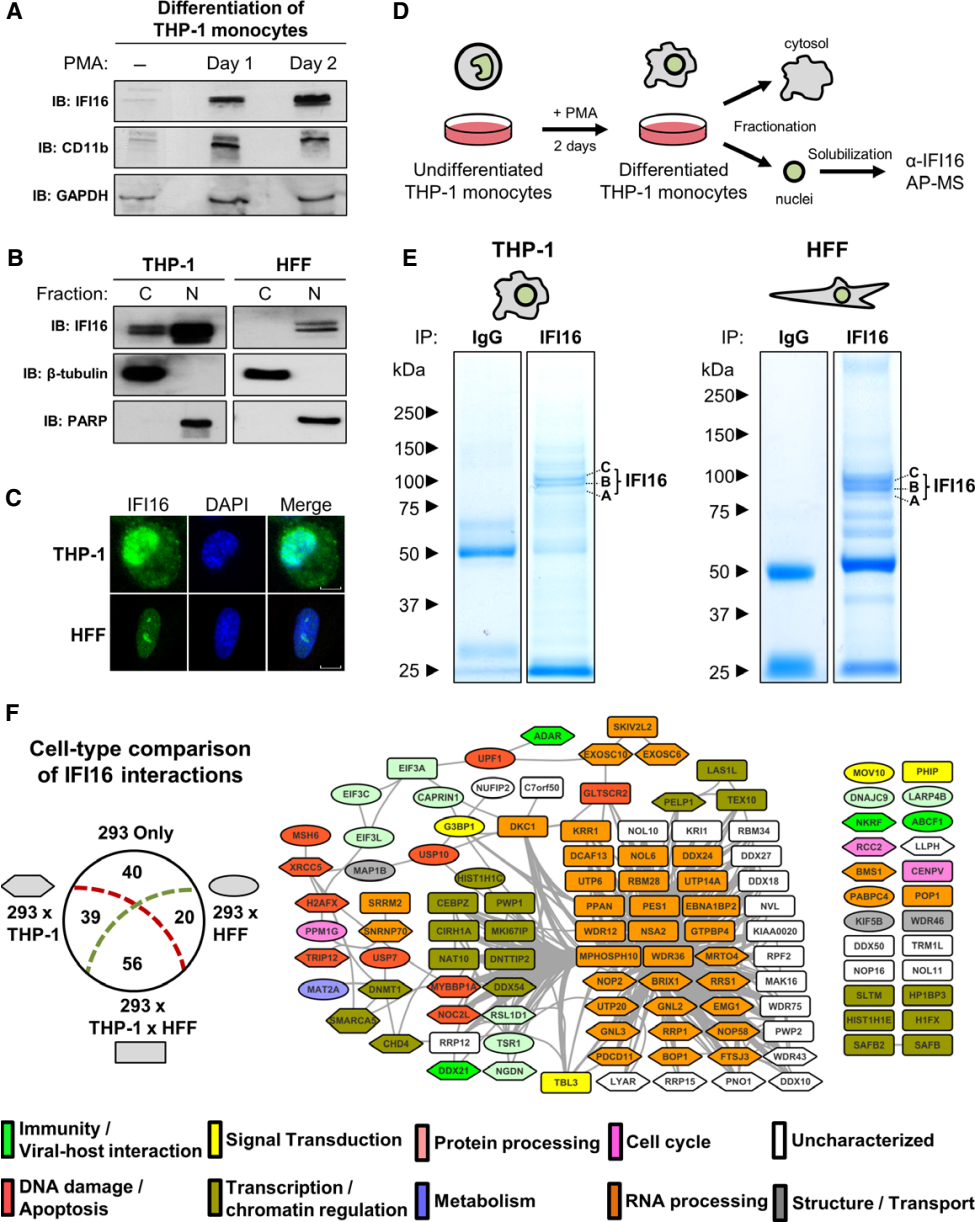
Endogenous IFI16 interactions in differentiated monocytes and primary fibroblasts A Differentiation of THP-1 monocytes was monitored by Western blotting against CD11b after PMA treatment. IFI16 expression was probed.

Based on our analysis in HEK293 cells, the identified interactions represent a range of housekeeping functions of the PYHIN proteins. While the majority of PYHIN interactions observed in HEK293 cells (Fig3) remained fairly stable even after re-scoring against additional negative controls from the CRAPOME database (Mellacheruvu *et al*, 2013) (see Supplementary Fig S9), we next assessed the conservation of these interactions in additional cell types. Identification of conserved interactions would not only validate our findings, but also point to core functional interactions. We selected to focus on IFI16, as we have already optimized the immunoaffinity purification of endogenous IFI16 using a mixture of two monoclonal antibodies that recognize all three IFI16 isoforms (Li *et al*, 2012). This analysis could not be performed for the other PYHIN proteins, as antibodies for effective immunoisolation are not yet available. We compared IFI16 interactions in HEK293 cells to those in primary human fibroblasts (HFF) and differentiated monocytes (THP-1), given the use of these cells in characterizing antiviral factors (Muruve *et al*, 2008; Burckstummer *et al*, 2009; Unterholzner *et al*, 2010; Orzalli *et al*, 2012; Li *et al*, 2013). The levels of endogenous IFI16 were induced upon differentiation of monocytes into macrophages (Fig4A and D). Similar to the expression of IFI16-GFP in our HEK293 cell lines, the localization of endogenous IFI16 was observed to be predominantly nuclear in fibroblasts by cellular fractionation (Fig4B) and immunofluorescence microscopy (Fig4C). However, in differentiated THP-1 monocytes, a subset of IFI16 was also present in the cytoplasm (Fig4B and C). Therefore, for an appropriate comparison to IFI16 interactions in HEK293 cells, AP-MS analysis was performed using the nuclear THP-1 fractions (Fig4D). To address non-specific interactions, we performed parallel IgG isolations from THP-1 (*N* = 2) and HFF (*N* = 2) cells (Fig4E). We increased the stringency of the specificity filter by including additional control immunoisolations from GFP-expressing HEK293 cells (*N* = 3) and employed a similar SAINT scoring strategy and threshold (≥ 0.85) as above (Supplementary Tables S7 and S8). Overall, out of the 155 IFI16 interactions in HEK293 cells, nearly 75% (115 proteins) were confirmed in at least one other cell type. 56 of these were present in all three cell types, while 39 and 20 interactions were shared between HEK293 and THP-1, and HEK293 and HFF, respectively (Fig4F, left). Similar to the STRING analysis of IFI16 interactions in HEK293 cells, the most prominent functional protein classes of transcription, ribosomal biogenesis, and RNA processing were conserved (Fig4F, right). Among the shared interactions, we also observed proteins with roles in immune response, such as NKRF and ABCF1. Interestingly, 88% (23 out of 26) of the proteins with uncharacterized gene ontology molecular functions were retained among the IFI16 interactomes (Fig4F, white nodes). Therefore, further investigation into the cellular function of these proteins is likely to provide insight into the cell type-independent housekeeping roles of IFI16. The large percentage of IFI16 interactions identified in HEK293 cells and conserved across significantly different cell types highlights the importance of the PYHIN interactome delineated in our inducible cell system. Therefore, we next focused on the interactions of the least characterized PYHIN protein, IFIX.

B Cytoplasmic (C) and nuclear (N) fractions of differentiated THP-1 monocytes and HFFs were probed for IFI16 by Western blot. Fractionation efficiency was confirmed by the enrichment of β-tubulin or PARP in C or N fractions, respectively.

C Localization of endogenous IFI16 in THP-1 and HFFs by fluorescence confocal microscopy. Scale bars, 5 μm.

D Workflow for analysis of IFI16 complexes in THP-1 nuclei.

E Proteins from endogenous IFI16 complexes and IgG controls from THP-1 (left) and HFFs (right) were visualized by SDS–PAGE and Coomassie staining. IFI16 isoforms are indicated.

F Comparison of the 155 IFI16 SAINT-filtered interactions in HEK293 cells with SAINT-filtered interactions from THP-1 and HFF cells. The number of IFI16 interacting proteins unique to HEK293 or shared with THP-1 (293 × THP-1), HFF (293 × HFF), or between all three cell types (293 × THP-1 × HFF) are illustrated (left). The core set of 115 IFI16 interactions shared between HEK293 and at least one other cell type was visualized by STRING (right). Node shape indicates the cell type overlap (node key in diagram, left), while node color indicates protein molecular function based on gene ontology classification.

### IFIX interacts with components of PML bodies and DNA damage response pathways

**Figure 5:**
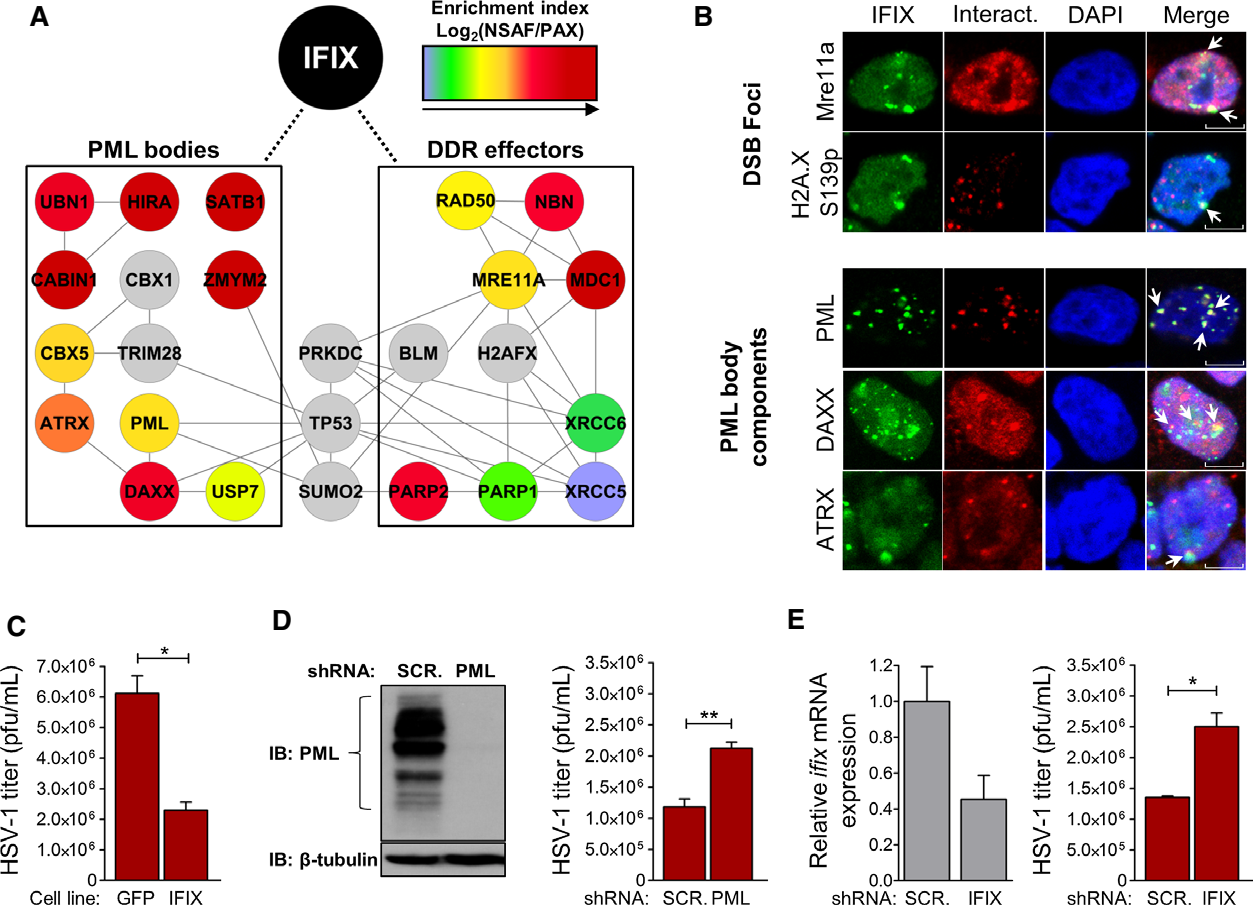
IFIX interactions with PML bodies and DNA damage effectors reflect antiviral properties A SAINT-filtered IFIX interactions related to PML body function or DNA damage response were assessed by STRING and visualized in Cytoscape. Node colors correspond to the log_2_-transformed NSAF/PAX values. Several interactions failing to pass SAINT, but enriched ≥ twofold relative to GFP control immunoisolations were added manually (gray color).

Considering what little is known about the cellular functions of IFIX, we were intrigued by its interactions with several components of nuclear PML body structures and with DDR effectors. The prominence of these interactions is supported by their presence in both N- and C-terminally tagged IFIX IPs (Fig2A, Supplementary Fig S6, and Supplementary Table S4) and by their enrichment as assessed by NSAF/PAX analysis (Fig5A). Furthermore, although they failed to pass our SAINT filter criteria, several additional prey proteins integral to these cellular pathways had enriched spectral counts relative to the GFP control isolations. These included CBX5 interacting partners CBX1 (2.7-fold) and TRIM28 (3.8-fold), DNA damage response effector proteins PRKDC (3.4-fold) and histone variant H2AFX (2.8-fold), small ubiquitin-like modified SUMO2 (3.0-fold), and tumor suppressor TP53 (2.0-fold enriched). In agreement with its association with DNA repair pathway components, we observed a partial co-localization of IFIX with proteins known to be enriched at DSB foci—MRE11A and histone variant H2AFX phosphorylated at serine 139 (Fig5B). IFIX was also excluded from several DSB foci, suggesting its selective recruitment to sites of dsDNA repair.

B Co-localization of IFIX-GFP with selected interactions in HEK293 cells was assessed by fluorescence confocal microscopy (white arrows). Scale bars, 5 μm.

C IFIX-GFP-expressing HEK293 cells were infected at MOI = 0.1 with HSV-1. At 72 h post-infection, progeny virus was harvested and titered. Mean values ± SEM (*n* = 3) are shown. *P ≤ 0.05, compared to control GFP cells.

D PML knockdown efficiency in HFFs relative to shScrambled (shSCR)-HFFs was assessed by Western blot (left). shPML-HFFs were infected at MOI = 0.1 with HSV-1. Progeny virus was harvested at 48 h post-infection and titered (right). Mean values ± SEM (*n* = 3) are shown. **P ≤ 0.01, compared to shSCR-HFFs.

E *ifix*mRNA levels in shIFIX-HFFs were assessed by RT–qPCR to determine knockdown efficiency (left). mRNA levels were normalized to cellular *β-actin* levels. The basal level of *ifix* expression in shSCR HFF cells relative to actin was 1.8E-5, which corresponds to a raw Ct value of ˜31. shIFIX-HFFs were infected at MOI = 0.1 with HSV-1. Progeny virus was harvested at 48 h post-infection and titered (right). Mean values ± SEM (*n* = 3) are shown. *P ≤ 0.05, compared to shSCR-HFFs. Data information: In (C–E), Student's unpaired *t*-test; two-tailed.

Importantly, co-isolated with IFIX was PML, the integral PML body protein, along with other proteins that transiently or conditionally localize to PML bodies (i.e., ATRX, DAXX, USP7, SATB1, CBX5, ZMYM2, CABIN1, HIRA, UBN1) (Figs3 and 5A). Within this subset, several sub-complexes, including DAXX/ATRX and CABIN1/HIRA/UBN1, are known to be involved in heterochromatinization and transcriptional repression in both uninfected and virus-infected cells (Tavalai *et al*, 2006; Lukashchuk & Everett, 2010; Rai *et al*, 2011; Glass & Everett, 2013). As a validation of the IFIX association with PML bodies, we observed co-localization of IFIX with PML within sub-nuclear punctate structures (Fig5B, white arrows). The nuclear IFIX puncta also displayed some partial overlap with the PML body components ATRX and DAXX (Fig5B, white arrows). As expected, being only transiently associated with PML bodies, ATRX and DAXX did not localize exclusively to these puncta (Weidtkamp-Peters *et al*, 2008). PML is well established to act as a scaffold for the formation of nuclear bodies with punctate appearance (Lallemand-Breitenbach & de The, 2010). Therefore, it is unlikely that IFIX puncta induces the recruitment of PML. Rather, our results suggest that IFIX is likely recruited, through mechanisms still to be determined, to PML-containing nuclear bodies.

### IFIX is an antiviral factor

Previous studies have established critical functions for PML bodies in mediating defense, IFN-dependent immunological and apoptotic responses to viral infection (Djavani *et al*, 2001; Tavalai *et al*, 2006; Lukashchuk & Everett, 2010; Glass & Everett, 2013). Considering our identification of an IFIX-PML interaction, in conjunction with IFIX being a member of the PYHIN family of immune regulators, we hypothesized that IFIX may have antiviral functions. To test this hypothesis, we first assessed the impact of IFIX overexpression on the ability of HSV-1 to replicate within our inducible HEK293 cell system. By plaque assay, HSV-1 progeny titers were markedly reduced (approximately threefold) in IFIX-GFP-expressing cells relative to GFP control cells (Fig5C). We next sought to validate IFIX antiviral functions in primary human fibroblasts which are highly permissive and susceptible to HSV-1 infection. As PML is known to limit the replicative capacity of HSV-1, we first generated HFFs stably expressing shRNAs targeting all the isoforms of PML (Fig5D, left). As expected, HSV-1 titers were significantly greater in PML-depleted fibroblasts relative to the scrambled shRNA control (Fig5D, right). Having confirmed this, we next generated HFFs stably expressing shRNAs targeting all isoforms of IFIX. IFIX knockdown was confirmed by RT–qPCR due to the lack of available IFIX antibodies (Fig5E, left). Similar to PML-depleted fibroblasts, knockdown of IFIX significantly increased HSV-1 titers relative to control cells (Fig5E, right). Altogether, these results show an inverse correlation between IFIX expression and HSV-1 replication efficiency, supporting an antiviral role for IFIX during herpesvirus infection.

### The HIN200 domain of IFIX interacts with dsDNA in a sequence-independent manner

Our observations that IFIX physically interacts with PML body components (Fig5A and B) and that it affects HSV-1 replication efficiency (Fig5C–E) implicate its involvement in eliciting antiviral defense responses. Therefore, we next asked through what mechanism IFIX exerts its antiviral function. It is established that upon infection PML nuclear bodies reform directly adjacent to the site at which nuclear-replicating viruses deposit their nucleic acid genome. In response, viruses are known to either degrade, reorganize, or co-opt PML body contents for structuring intranuclear viral replication centers (Doucas *et al*, 1996; Ishov & Maul, 1996; Ahn *et al*, 1998; Chelbi-Alix & de The, 1999; Adamson & Kenney, 2001; Everett *et al*, 2007; Glass & Everett, 2013). Therefore, the recruitment of IFIX to PML bodies may provide the opportunity for binding to viral DNA. Furthermore, IFIX has a substantial sequence conservation with IFI16, the latter of which has been characterized by us and others as a nuclear sensor of herpesviral dsDNA (Kerur *et al*, 2011; Li *et al*, 2012, 2013; Orzalli *et al*, 2012; Johnson *et al*, 2013), and, more recently HIV proviral dsDNA (Jakobsen *et al*, 2013). In light of these observations, we hypothesized that IFIX may also sense and respond to viral DNA.

**Figure 6:**
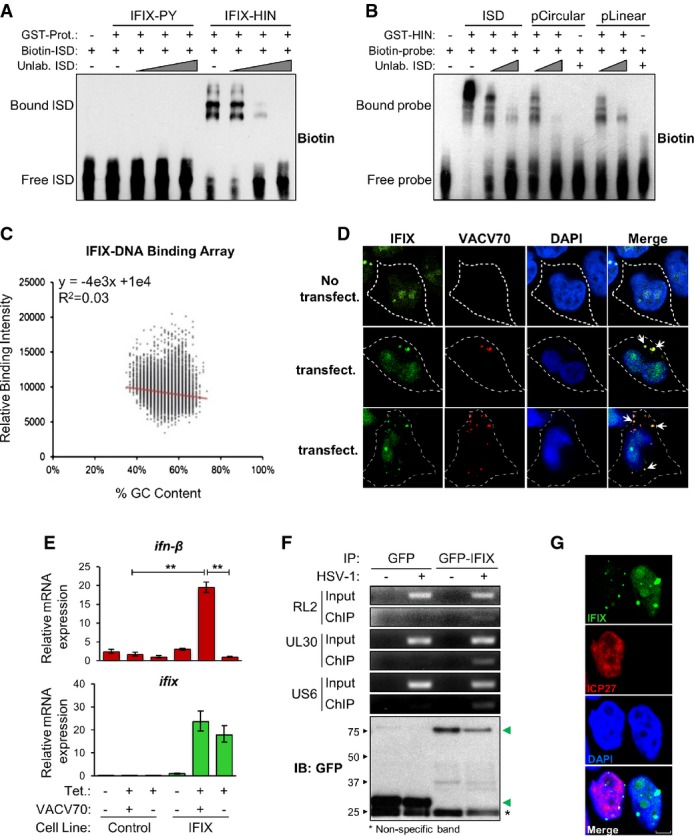
IFIX is a DNA-binding protein that functions as a viral DNA sensor A Biotinylated ISD was incubated with either recombinant GST-tagged IFIX-PY (aa 1–100) or IFIX-HIN200 (aa 200–492) and competed off with unlabeled ISD. DNA–protein complexes were resolved by non-denaturing PAGE, and biotinylated substrates were visualized.

Considering that immune sensors are activated via direct interaction with their cognate ligand, we first assessed the ability of IFIX to bind pathogenic dsDNA *in vitro*. To determine the domain of IFIX mediating such binding, we generated PY-only (amino acids 1–100; herein IFIX-PY) and HIN200-only (amino acids 200–492; herein IFIX-HIN200) truncations. For the binding assays, we used the interferon-stimulating dsDNA (ISD) sequence, previously used in studies of IFI16 (Unterholzner *et al*, 2010). As shown by electrophoretic mobility shift assay (EMSA) (Fig6A), the purified IFIX-HIN200 effectively binds biotinylated ISD, and this association could be titrated away using unlabeled ISD probe. In contrast, no shift was observed using purified IFIX-PY. Thus, the HIN200 domain of IFIX is both necessary and sufficient for binding dsDNA.

B Biotinylated ISD, pcDNA3.1, or *Bam*HI-linearized pcDNA3.1 were incubated with recombinant GST-tagged IFIX-HIN200 and competed off with unlabeled ISD. DNA–protein complexes were resolved as above.

C Recombinant GST-tagged IFIX-HIN200 was incubated with a dsDNA array consisting of all possible 10mer oligonucleotide sequences, and DNA–protein binding was visualized with a fluorescent GST-specific antibody. To illustrate IFIX-HIN200 sequence preferences, fluorescence intensity is plotted against oligomer sequence content.

D IFIX-GFP HEK293 cells were transfected with Cy3-labeled VACV 70mer and imaged by fluorescence confocal microscopy (315× zoom factor). White arrows indicate co-localization between IFIX-GFP and VACV 70mer.

E Control or IFIX-GFP HEK293 cells were transfected with VACV 70mer, and total RNA was collected after 6 h. Relative induction of *ifn-β* was quantified by RT–qPCR. mRNA levels are normalized to cellular *gapdh* levels. The basal expression levels in the IFIX-inducible HEK293 cells for *ifn-β* (right bar at +Tet/-VACV70) and *ifix* (left bar at -Tet/-VAVC70) relative to *gapdh* were 2E-6 and 3E-6, respectively. This corresponds to raw C_T_ values of ˜28 and 27 for *ifn-β* and *ifix*, respectively. Mean values ± SEM (*n* = 3) are shown. **P ≤ 0.01 compared to transfected control cells and mock-transfected IFIX-GFP-expressing cells (Student's unpaired *t*-test; two-tailed).

F GFP or GFP-IFIX HEK293 cells were infected with HSV-1 (MOI = 5) and cross-linked at 7 h post-infection. Cell lysates were subjected to α-GFP immunoaffinity isolation, and viral DNA binding was assessed by RT–PCR using HSV-1-specific genome sequences (top). Isolated GFP or GFP-IFIX (green arrows) was assessed by Western blotting (bottom).

G Localization of IFIX-GFP in HSV-1-infected HEK293 cells (MOI = 1; 4 h post-infection) was visualized by fluorescence confocal microscopy. Viral protein ICP27: marker for infection. Scale bars, 5 μm.

Several IFIX interactions we identified are related to dsDNA break repair functions and can selectively bind dsDNA ends, such as MRE11A/NBN/RAD50 and XRCC5/6 (Ku80/70) (Pierce *et al*, 2001; de Jager *et al*, 2002). Therefore, we asked whether the IFIX-HIN domain binds preferentially to dsDNA ends. A gel shift assay demonstrated that the recombinant GST-HIN200 domain effectively bound both circularized pcDNA3.1 (pCircular) and *Bam*HI-linearized pcDNA3.1 (pLinear) DNA substrates with equivalent efficiency (Fig6B). Again, IFIX-plasmid DNA binding could be titrated away using excess ISD probe. Thus, IFIX did not appear to preferentially bind dsDNA ends. To further characterize the nature of IFIX-DNA binding, we utilized a dsDNA array containing all possible 10mer oligonucleotide sequences, allowing us to probe for DNA sequence preferences of the IFIX HIN200 domain. According to the distribution and intensities of binding events to the array, the recombinant GST-HIN200 protein did not display any clear sequence specificity (Fig6C). This is consistent with previous structural and biochemical analyses of IFI16 and AIM2 that have demonstrated electrostatic interactions and hydrogen bonding between the HIN200 domains and DNA phosphate–sugar backbone predominantly contribute to PYHIN-DNA binding (Jin *et al*, 2012). As a lack of structural or sequence specificity is critical for the wide and effective recognition of intracellular pathogen DNA during infection, these results further suggest that IFIX may function as a DNA sensor.

### IFIX functions as an innate immune sensor of viral DNA

Having established that IFIX can bind a wide range of dsDNA substrates, we next assessed whether it can localize to viral dsDNA in cells. As we have previously employed an inducible HEK293 cell system to characterize IFI16 DNA sensing functions (Li *et al*, 2012), we opted to test IFIX functions using a similar system. HEK293 cells expressing IFIX-GFP were transfected with Cy3-labeled vaccinia virus (VACV) 70mer (Fig6D), a potent dsDNA immunostimulant (Unterholzner *et al*, 2010). Interestingly, as early as 2 h post-transfection, we observed the formation of IFIX-GFP foci that co-localized with VACV 70mer in the cytoplasm. These cytoplasmic IFIX-containing puncta were exclusively observed in cells successfully transfected with VACV 70mer (Fig6D). As a control, IFI16-GFP displayed similar cytoplasmic foci staining, co-localizing with the VACV 70mer (Supplementary Fig S10). Since the criteria for a host factor to be considered a DNA sensor include both binding to pathogenic DNA and triggering antiviral cytokine expression, we next tested whether IFIX is required for inducing a cytokine response to VACV 70mer. HEK293 cells expressing IFIX-GFP were challenged with VACV 70mer for 6 h, after which *ifn-β* mRNA levels were measured by RT–qPCR (Fig6E). Relative to transfected control cells, IFIX-GFP-expressing cells stimulated with VACV 70mer displayed approximately a 20-fold induction of *ifn-β* mRNA. IFIX-GFP overexpression alone did not induce the expression of *ifn-β*. Thus, similar to IFI16, IFIX can function as a DNA sensor to stimulate type I interferon expression.

Our data establish IFIX as an innate immune sensor of cytoplasmic DNA. Nevertheless, we and others have recently introduced the concept of sensing viral DNA within the nucleus (Li *et al*, 2012; Orzalli *et al*, 2012), identifying IFI16 as currently the only known nuclear sensor. Given the predominant nuclear localization of IFIX, we next examined whether it could also detect viral DNA in the nucleus in HSV-1-infected cells. In addition to our results demonstrating that IFIX limits its replication, we specifically chose HSV-1 as it targets its dsDNA genome exclusively to the nucleus during infection (Li *et al*, 2012; Orzalli *et al*, 2012). Furthermore, the HSV-1 genome is prone to the transcriptional repression functions of PML body components (Everett *et al*, 2008; Lukashchuk & Everett, 2010; Cuchet-Lourenco *et al*, 2013), several of which we identified to physically interact with IFIX. HEK293 cells expressing GFP-IFIX or GFP alone were infected with HSV-1. At 7 h post-infection (hpi), we assayed the association of IFIX or GFP with the HSV-1 genome by chromatin IP and PCR (Fig6F). Only GFP-IFIX associated with a detectable amount of HSV-1 DNA, indicating that IFIX is physically recruited to HSV-1 DNA during infection. Furthermore, using immunofluorescence microscopy, we observed that IFIX retained its nuclear localization following infection. Interestingly, IFIX was still present within nuclear puncta, while its nucleolar and diffuse nucleoplasmic localization appeared reduced (Fig6G and Supplementary Fig S11). As IFIX interaction with PML within nuclear puncta is already observed in uninfected cells, we do not expect that punctate formation is specifically induced by infection. In agreement with this retained nuclear localization, numerous IFIX interactions were conserved in both uninfected and infected cells (Supplementary Fig S12 and Supplementary Table S12). Among the most prominent shared SAINT-filtered interactions were members of the 5FMC complex (LAS1L, SENP3, TEX10, WDR18), also identified as a prominent IFIX-associated complex in uninfected cells. In fact, the 5FMC components were each enriched approximately 3- to 4-fold in IFIX isolations during HSV-1 infection relative to uninfected cells, suggesting an increased association in response to infection. Other shared interactions included proteins known to be involved in immune response, TRIM26 and sequestosome 1 (SQSTM1), the latter of which modulates the activation of NF-κB and is important regulator of autophagy (Pankiv *et al*, 2007; Duran *et al*, 2008). Although detected in both uninfected and HSV-1-infected states, these two interactions were highly enriched and assessed as specific (by SAINT) only following infection. Whether these interactions play roles in housekeeping functions of IFIX or in its antiviral roles remains to be elucidated. It is conceivable that the tetracycline induction of IFIX partly mimics a cellular state in which IFIX is induced by interferon, which potentially explains why many interactions are shared between uninfected and infected states. However, we did observe several interactions unique to infected cells. These included the viral protein UL39, which possesses anti-apoptotic activities and is required for efficient HSV-1 replication (Dufour *et al*, 2011). UL39-IFIX interaction may represent either a viral mechanism for suppressing IFIX functions or a host mechanism for limiting HSV-1 propagation. Furthermore, as expected, the association of IFIX with PML body components PML, ATRX, and DAXX was not detected following infection, as PML is known to be rapidly targeted for degradation by HSV-1 ICP0 (Chelbi-Alix & de The, 1999). Together, our results indicate that IFIX can detect nuclear HSV-1 DNA, establishing it as only the second identified protein capable of sensing viral DNA within the nucleus.

## Discussion

One of the greatest advantages of MS-based interactome analyses is the ability to unbiasedly capture and unambiguously identify a wide-spectrum of interactions for any target protein. Such interactome studies can highlight cellular pathways to which the protein of interest contributes, as well as provide direct insights into mechanisms through which a protein exerts its functions. Therefore, when integrated with other molecular biology- and microscopy-based functional analyses, these ‘omic’ tools are invaluable for defining the functions of uncharacterized protein. Here, we described a streamlined, integrative proteomics approach for characterizing the protein interaction network of the entire human PYHIN family of immune regulators. Although PYHIN proteins have been implicated in regulating immune, inflammatory, apoptotic, and tumorigenic cellular processes, their interactions have remained largely undefined, making our analyses both essential and challenging. By integrating differential tagging, microscopy, immunoaffinity purification, quantitative mass spectrometry, bioinformatics clustering, and functional network analysis, we defined common and distinct features of PYHIN proteins. Overall, we identified over 300 previously unreported PYHIN interacting proteins with roles in antiviral defense, intracellular signaling, DNA damage response, apoptosis, and transcriptional regulation.

Interactions shared between IFI16 and MNDA were functionally enriched in ribosome biogenesis and rRNA processing. This is particularly interesting as both IFI16 and MNDA have been implicated as tumor suppressors, functioning as regulators of cell cycle and apoptotic processes (Song *et al*, 2008, 2010; Liao *et al*, 2010; Sun *et al*, 2014). Several recent studies have linked activation of a ribosomal stress pathway with other p53-activating pathways, such as DNA damage response (Zhu *et al*, 2009; Llanos & Serrano, 2010; Morgado-Palacin *et al*, 2012). It is the collaboration between the components of these canonical cellular stress responses that is thought to be necessary for maximal p53 activity. IFI16 has already been implicated in controlling p53-dependent transcription in response to DNA damage (Aglipay *et al*, 2003; Fujiuchi *et al*, 2004; Liao *et al*, 2010). Furthermore, the majority of the interactions we identified for IFI16 in HEK293 cells were shared with endogenous IFI16 isolated from monocytes and fibroblasts (Fig4F). These interactions may point to mechanisms by which IFI16 and MNDA could act as tumor suppressors.

Also prominent within our PYHIN interactome were proteins involved in transcriptional regulation. For instance, we identified interactions between IFI16 and nucleosome and chromatin remodeling proteins, including SMARCA5 (along with fellow B-WHICH complex components MYBBP1A and DDX21), CHD4, HP1BP3, and SAFB/SAFB2. Other IFI16-associated transcription factors included NKRF, CIRHI1A, and SLTM. While these interactions were confirmed in THP-1 and HFF cells, not all associations with transcriptional regulation functionality were shared between cell types. For example, components of the functionally linked PBAF and WINAC ATP-dependent chromatin remodeling complexes (e.g., ARID1A, SMARCA2, SMARCA4, SMARCC1, PBRM1, BAZ1B) were uniquely enriched in IFI16 complexes isolated from THP-1 monocytes. These remodeling complexes (among others) have well-documented roles in chromatin regulation during 1,25(OH)_2_D3-mediated transcriptional repression, which proceeds through recruitment of the VDR–RXR vitamin D receptor to vitamin D receptor response elements (Haussler *et al*, 1997). This is an important process in differentiation within the myeloid cell lineage. Curiously, another uniquely prominent IFI16 association in THP-1 cells was MNDA, which interestingly is also linked with 1,25(OH)_2_D3 signaling to cause differentiation of monocytes (Gaczynski *et al*, 1990). Given the genetic interaction of IFI16 and MNDA (both are located on human chromosome 1q) and similar expression pattern in the hematopoietic system, it is tempting to speculate some sort of cooperativity or convergence of activities involving vitamin D receptor signaling. More broadly, these data support previous reports that PYHIN proteins can function in either transcriptional activation or repression (Johnstone *et al*, 1998; Xie *et al*, 1998; Johnstone & Trapani, 1999; Chen *et al*, 2006). In particular, recent studies have shown that IFI16 can modulate the expression of both host and viral genes (Orzalli *et al*, 2013). Our interactome points toward a similar function for IFIX, as we observed its abundant association with the 5FMC complex (TEX10, SENP3, LAS1L, WDR18) in both uninfected and HSV-1-infected cells.

**Figure 7:**
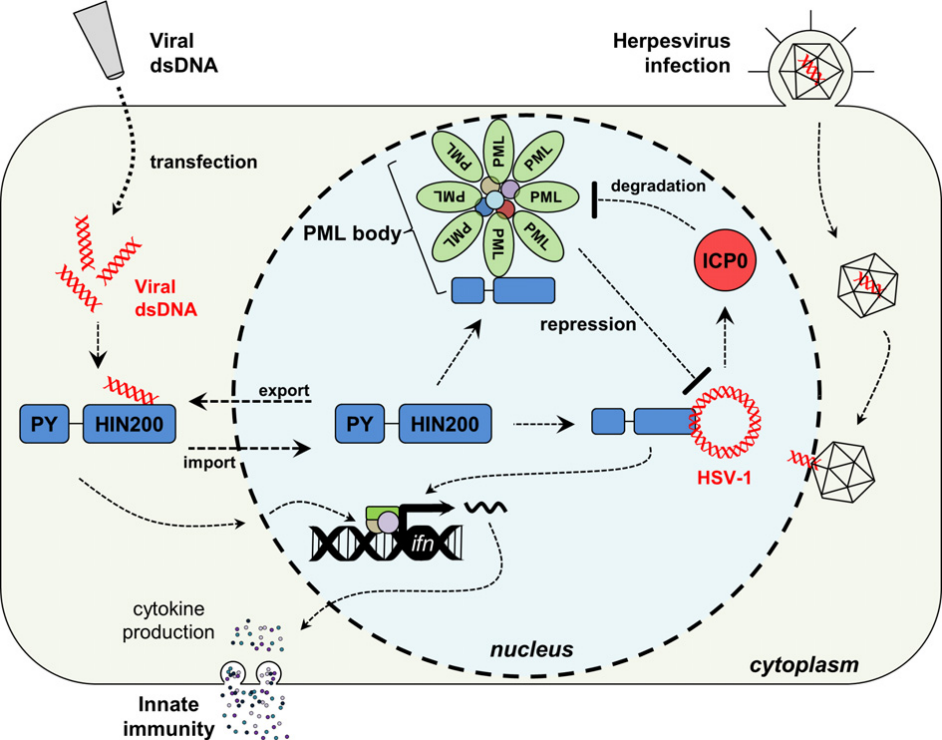
Model for IFIX-mediated intrinsic and innate immune functions IFIX functions as a previously unrecognized viral DNA sensor. IFIX binds viral DNA in the cytoplasm through its HIN200 domain. This initiates innate immunity via induction of interferon-β expression. During HSV-1 infection, IFIX recognizes and binds viral DNA within the nucleus. This IFIX function may be mediated by its association with sub-nuclear PML bodies. To inhibit PML body-mediated intrinsic defenses, HSV-1 E3 ubiquitin ligase ICP0 is known to target PML for its proteasome-dependent degradation, dispersing PML bodies and relieving the imposed repression.

Among the PYHIN interactions identified in this study, those between IFIX and the components of PML bodies were some of the most striking and unexpected. These sub-nuclear proteinaceous foci are scaffolded via self-association of the integral protein PML. Since their discovery, PML bodies have been demonstrated to play central roles in regulating apoptosis, DNA damage repair, transcription, and viral infection. For instance, a PML-associated ATRX-DAXX complex has been demonstrated to chromatinize and transcriptionally repress herpesviruses during infection, while a HIRA-UBN1 complex is thought to coordinate senescence-associated heterochromatinization (Banumathy *et al*, 2009). In fact, given their prominence in antiviral defenses, PML body components are commonly targeted and inhibited by viral proteins. Furthermore, DNA damage pathways are known to be induced during infections with nuclear-replicating DNA viruses (Schwartz *et al*, 2009; Lilley *et al*, 2011). The functions of PML bodies and DDR factors in host defense are further intertwined. For instance, the MRN complex elicits both DNA damage and dsDNA-dependent IFN responses (Kondo *et al*, 2013) and localizes to PML bodies (Lombard & Guarente, 2000; Mirzoeva & Petrini, 2001). Our observed IFIX interactions with PML bodies, Ku70/80, and MRN complexes led us to hypothesize that these proteins may act cooperatively in antiviral response upon deposition of viral genome, and we went on to more closely investigate these possible IFIX functions. Overexpression of IFIX significantly hindered the ability of HSV-1 to replicate in HEK293 cells (Fig5E). Reciprocally, knockdown of IFIX in primary fibroblasts increased HSV-1 titers to an extent comparable to that of a PML knockdown in the same cell type. Therefore, we sought to investigate the mechanism by which IFIX exerts antiviral activities. Using *in vitro* and *in vivo* DNA-binding assays, we demonstrated that IFIX binds foreign dsDNA through its HIN200 domain (model in Fig7). This physical interaction with DNA is not dependent on DNA ends (unlike the IFIX-associated MRN and Ku70/80 complexes we identified) and is not sequence specific (Fig6B and C). Furthermore, we showed that IFIX contributes to the induction of type I IFNs in response to transfection with vaccinia virus dsDNA. Thus, IFIX possesses both characteristics ubiquitous among all currently established DNA sensors: (i) an ability to bind foreign dsDNA in a sequence-independent manner and (ii) a contribution to the induction of innate immune signaling in response to dsDNA.

Interestingly, the predominantly nuclear protein IFIX rapidly co-localized within the cytoplasm with transfected viral dsDNA (Fig6D). The function of IFIX as a cytoplasmic DNA sensor is in agreement with the canonical notion that sensing of pathogenic DNA occurs exclusively in the cytoplasm. Nevertheless, recent studies from several research groups, including ours, have established that nuclear DNA sensing does exist. Thus far, IFI16 has been the only protein shown to have this nuclear function. Importantly, we demonstrate here that IFIX binding to viral DNA was not limited to the cytosol. Following infection with the nuclear-replicating herpesvirus HSV-1, we observed not only binding of IFIX to viral dsDNA but also its retention within the nucleus during infection. Interestingly, we observed the localization of IFIX-GFP within nuclear punctate structures upon HSV-1 infection (Fig6G and Supplementary Fig S11). Furthermore, we found that many of IFIX interactions were lost following infection, including PML and its associated proteins (DAXX, ATRX, HIRA, etc.). This was expected as the HSV-1 viral E3 ubiquitin ligase protein, ICP0, targets PML for degradation early in infection and disperses PML body components (Everett & Maul, 1994; Chelbi-Alix & de The, 1999; Everett *et al*, 2008). As this event is critical for the efficient replication of HSV-1, this observation underscores the role of IFIX as an antiviral factor. It is tempting to speculate that IFIX normally exerts its antiviral activities through its interaction with PML bodies and that, upon degradation and reorganization of PML bodies by HSV-1 ICP0, it is re-assigned to performing either auxiliary or non-restrictive cellular or viral functions (Fig7). Our previous finding that IFI16 is co-opted by HCMV to induce expression of viral genes from the major immediate-early promoter supports this model (Cristea *et al*, 2010). In addition to IFIX interactions disrupted during infection, our current study also identified several interactions conserved in uninfected and HSV-1-infected cells. In fact, members of the 5FMC complex were enriched during infection relative to uninfected cells. As we see a significant decrease in HSV-1 progeny titers in the same cell line (Fig5C), it is possible that the IFIX-5FMC interaction may act as an inhibitor of viral gene transcription. Specifically, the transcriptional regulator targeted by the 5FMC complex, ZNF148, binds the canonical GC box sequence prevalent within both eukaryotic and viral (e.g., HSV-1) gene promoter regions (Merchant *et al*, 1996; Law *et al*, 1998). Furthermore, ZNF148 is known to compete with transcription factor SP1 binding within GC box regions, subsequently inhibiting the transactivation of SP1 target genes (De Bustos *et al*, 2005). As SP1 is a known activator of HSV-1 gene expression (Kim & DeLuca, 2002), the IFIX-5FMC interaction may inhibit HSV-1 transcription through displacement of SP1. Alternatively, the IFIX-5FMC interaction may reflect the co-opting of IFIX by HSV-1 to regulate cellular transcription.

Our findings highlight IFIX as the second protein recognized to date to sense viral DNA in the nucleus, thereby expanding the concept of nuclear sensing. It is interesting to consider our results in the context of other identified DNA sensors. In particular, the recently reported cyclic GMP-AMP synthase (cGAS) (Sun *et al*, 2013), which synthesizes the endogenous STING agonist cyclic di-nucleotide cGAMP in response to cytosolic DNA (Ablasser *et al*, 2013a; Wu *et al*, 2013), is an important component of the cellular immune signaling pathway. However, we and others have demonstrated that IFI16 (Unterholzner *et al*, 2010; Li *et al*, 2012; Orzalli *et al*, 2012), and now IFIX (in this study), also contributes to the expression of IFN-β in response to foreign DNA. Considering that IFI16, like cGAS, requires STING for its putative immune signaling functions, IFI16 may operate within the same signaling pathway and directly or indirectly promote the function of cGAS, as recently suggested (Thompson *et al*, 2014). This may also be the case for IFIX. However, both IFIX (as shown in this study) and IFI16 directly bind to viral DNA with high affinity via their HIN200 domains. Therefore, one alternative hypothesis is that IFI16 and IFIX directly contribute to transcriptional activation of type I interferons in the nucleus. This hypothesis is supported by the interactions with chromatin remodeling complexes and transcriptional regulators that we elucidate in our current study. This PYHIN-mediated transcriptional regulation would seemingly function against viruses twofold, as IFI16 was also previously shown to silence viral gene expression by restricting transcription from viral promoters during infection (Orzalli *et al*, 2013; Johnson *et al*, 2014). Overall, elucidating the possible independent or coordinated functions of cGAS, IFI16, and IFIX requires further study.

In summary, this global overview of PYHIN protein interactions provides a valuable resource for characterizing the functions of these important proteins in human health and disease. Our studies both demonstrate the effectiveness of an integrative proteomics approach in extrapolating protein functions based on interaction networks and define important immune functions mediated by the human PYHIN family.

## Materials and Methods

### Plasmids

The open reading frames (ORFs) of *ifi16b*,*mnda*, and *aim2* were all purchased from Addgene. The *ifix*α*1* ORF was a gift from Dr. Scott S. Terhune (Medical College of Wisconsin). N- and C-terminal GFP fusions were made by subcloning *pyhin* ORFs into pEGFP-C3 and pEGFP-N1, plasmids (Clontech), respectively, using *Xho*I and *Bam*HI restriction sites. For constructing inducible HEK293 cell lines, *gfp* and *pyhin-gfp* fusions were subcloned into pcDNA5/FRT/TO vector. For *gfp* and N-terminal fusions, *Bam*HI and either *Xho*I or *Hind*III restriction sites were utilized. For subcloning C-terminal fusions, *Xho*I and either *Kpn*I or *Not*I restriction sites were used. For knockdown of all PML isoforms in HFFs, overlapping forward and reverse oligos containing an anti-PML shRNA sequence (Everett *et al*, 2007) were annealed and cloned into the lentivirus packaging vector pLK0.1 (The RNAi Consortium; Addgene Plasmid 10878) using *Age*I and *Eco*RI restriction sites. For knockdown of all IFIX isoforms, pLK0.1 containing an anti-IFIX shRNA sequence (TRCN0000107296) was purchased from Thermo Scientific. Lentiviral helper vectors psPAX2 (Addgene; Plasmid 12260) and pMD2.G-VSV-G (Addgene; Plasmid 12259) were gifts from Dr. Thomas Shenk (Princeton University). To generate the described IFIX-GST truncations, the nucleotide sequences encoding the IFIX pyrin domain (amino acids 1–100) and the HIN200 domain (amino acids 200–492) were inserted into the pGEX-4T1 vector via the *Bam*HI and *Xho*I restriction sites. All DNA oligos and primer sequences used in this study are summarized in Supplementary Tables S1, S2, S3, S9, S10 and S11.

### Cell culture and generation of inducible GFP-tagged PYHIN protein cell lines

Inducible HEK293 cells (gift from Dr. Loren W. Runnels, UMDNJ-RWJMS) and primary human foreskin fibroblasts (gift from Dr. Hillary A. Coller, UCLA) were cultured in Dulbecco's modified Eagle's medium (Life Technologies, Carlsbad, CA, USA) at 37°C in 5% CO_2_. THP-1 monocytes (gift from Dr. Thomas E. Shenk, Princeton University) were cultured in Roswell Park Memorial Institute 1640 medium (Life Technologies) at 37°C in 5% CO_2_. Prior to their use, THP-1 monocytes were differentiated using 1 μg/ml phorbol 12-myristate 13-acetate (PMA) (Sigma-Aldrich) for 48 h. All cell culture media were supplemented with 10% fetal bovine serum and 1% penicillin–streptomycin. To construct all inducible HEK293 cell lines, cells were co-transfected with pcDNA5/FTR/TO encoding either N- or C-terminally *gfp*-fused *ifi16b, ifix*α*1, aim2, or mnda* ORFs and the pOG44 helper plasmid. Stable cell lines were selected using 150 μg/ml hygromycin B and 10 μg/ml blasticidin (Life Technologies). For immunoaffinity purifications, cells were treated with 1 μg/ml tetracycline for 16 h to induce GFP-tagged PYHIN protein expression prior to harvesting cell lysates for immunoaffinity isolation. Cell populations were observed to be homogenously GFP-positive 16 h post-induction.

### Reagents and antibodies

VACV 70mer and ISD oligonucleotides were both synthesized as their two respective complementary single strands by Integrated DNA Technologies (Coralville, IA, USA). The sense strand was synthesized as either unlabeled, 5′-biotinylated, or 5′-Cy3-labeled forms. To generate double-stranded DNA (dsDNA), 10 μM sense strand and 10 μM antisense strand were mixed in annealing buffer (50 mM sodium phosphate buffer, pH 7.5, 150 mM NaCl, and 5 mM MgCl_2_), boiled at 95°C for 5 min, and then slowly cooled to room temperature. Liposome-mediated transfections of indicated DNA constructs were performed using Lipofectamine 2000 (Life Technologies) transfection reagents according to the manufacturer's instructions. An in-house developed rabbit polyclonal α-GFP antibody was used for immunoaffinity isolation of all GFP-tagged PYHIN proteins and for their detection by immunofluorescence and Western blotting. Immunoaffinity isolation and detection of endogenous IFI16 was accomplished using a 1:1 (w/w) mixture of two monoclonal α-IFI16 antibodies (ab500004 and ab55328). For Western blotting, α-tubulin (T6199; Sigma-Aldrich), α-STING (IMG-6422A; IMGENEX), α-IRF3 (sc9082; Santa Cruz Biotechnology), α-GAPDH (ab9484; Abcam), α-CD11b (555388; BD Biosciences), α-PARP (9542L; Cell Signaling), and α-ICP27 (sc-69806; Santa Cruz Biotechnology) antibodies were used. For immunofluorescence microscopy, α-PML (sc-9862; Santa Cruz Biotechnology), α-Daxx (sc-7152; Santa Cruz Biotechnology, Inc), α-ATRX (sc-15408, Santa Cruz Biotechnology), α-Mre11a (pab12381; Abnova), α-H2A.X phospho-Ser139 (05-636, Millipore) antibodies were used.

### Viruses

Wild-type HSV-1 (strain 17+) stocks were grown in U2OS cells. For infections, virus was diluted in DMEM supplemented with 2% (vol/vol) FBS and applied to cell monolayers at the indicated MOI. Cells were incubated with viral inoculums at 37°C for 1 h to allow the virus adsorb and enter, after which viral inoculums were removed and the cells washed once with PBS. Cells were then overlayed with DMEM supplemented with 2% (vol/vol) FBS, and the infection was allowed to proceed for the indicated amount of time. For measuring progeny virus titers, both cells and culture supernatants were collected at the indicated time, buffered using MNT (200 mM MES, 30 mM Tris–HCl, 100 mM NaCl, pH 7.4), sonicated and spun, serially diluted to the appropriate concentration, and titered on U2OS cells by plaque assay.

### Cryogenic cell lysis and immunoaffinity purification of PYHIN proteins

For interactome analyses, immunoaffinity-purified PYHIN proteins and their associated protein complexes were assessed via MS/MS analysis, as previously described (Cristea *et al*, 2005; Joshi *et al*, 2013). Specifically, cells were washed and collected in PBS, then snap-frozen in freezing buffer (20 mM K-HEPES, 1.2% polyvinylpyrrolidone (w/v), pH 7.4) via liquid nitrogen. For isolating nuclei from differentiated THP-1 monocytes, cells were first fractionated using a hypotonic buffer (10 mM HEPES, 10 mM KCl, 1.5 mM MgCl_2_, 1 mM DTT, PIC, 0.5% IGEPAL, pH 7.4.) and nuclei were washed extensively with PBS prior to snap-freezing. Frozen cell materials were ground using a Retch MM301 Mixer Mill (Retch, Newtown, PA, USA) for 1.5 min at 30.0 Hz, ten times, with intermittent re-cooling in liquid nitrogen. Half of a gram of the resulting cell powder was resuspended in 6 ml optimized lysis buffer [20 mM K-HEPES pH 7.4, 0.11 M KOAc, 2 mM MgCl_2_, 0.1% Tween-20 (v/v), 1 μM ZnCl_2_, 1 μM CaCl_2_, 0.6% Triton X-100, 200 mM NaCl, 10 μg/ml DNase I (alternatively, 100 U/ml benzonase for the isolations in differentiated THP-1 and HFF cells), 1/100 protease inhibitor cocktail (PIC, Sigma), 1/100 phosphate inhibitor cocktails 2 and 3 (PHICs, Sigma)] and was incubated at room temperature (RT) for 10 min to allow DNase I or benzonase digestion. The suspension was then homogenized for 30 s at 20,000 rpm using a PT 10-35 GT Polytron (Kinematica, Bohemia, NY, USA) and centrifuged at 7,000 *g* for 10 min at 4°C for clarifying the homogenate. The resulting pellet was solubilized in SDS sample buffer to assay for efficiency of protein solubilization by Western blotting. PYHIN proteins were immunoprecipitated for 1 h at 4°C from the supernatant using 7 mg M-270 epoxy magnetic beads (Life Technologies) conjugated with either 35 μg of an in-house developed rabbit polyclonal anti-GFP antibody or 35 μg of an equimolar mixture of two α-IFI16 monoclonal antibodies. Antibody-conjugated magnetic beads were prepared as previously described (Luo *et al*, 2010). Following immunoprecipitation, magnetic beads were extracted and washed six times with lysis buffer (lacking DNase I, PIC and PHICs). Immunoisolated protein complexes were eluted off the beads at 70°C for 10 min using TEL buffer (141 mM Tris, pH 8.5, 2% lithium dodecyl sulfate, and 0.51 mM EDTA). Proteins retained in the supernatant (‘flow-through’) were acetone precipitated and solubilized in SDS sample buffer for analyzing PYHIN protein isolation efficiency. After the first elution, beads were boiled in SDS sample buffer at 95°C for 10 min for assessing efficiency of elution.

### Sample preparation and mass spectrometry analysis

Immunoisolated protein complexes were reduced with 100 mM DTT at 37°C for 30 min, then digested in-solution with trypsin by a filter-aided sample preparation (FASP) method (Wisniewski *et al*, 2009), as described in Joshi *et al* (2013) and Kramer *et al* (2011). Specifically, protein samples were washed twice with UB buffer (8 M urea in 0.1 M Tris–HCl pH 8.0) in Microcon YM-30 ultrafiltration device (Millipore) followed by centrifugation (14,000 *g*) for 15 min. Proteins were then alkylated with 50 mM iodoacetamide in UB buffer at RT for 30 min, washed twice with UB buffer, and then finally washed twice with 50 mM ammonium bicarbonate (ABC). Proteins were digested with 5 ng/ml trypsin in 50 mM ABC at 37°C overnight. Following digestion, the filter unit was washed three successive times with water and centrifuged to collect the tryptic peptides. Peptides were then desalted with in-house packed C18 (Empore) stage tips, vacuum-concentrated, and acidified with 1% formic acid. For visualizing immunoisolates, an equal fraction of all IPs were resolved by 4–12% continuous gradient SDS–PAGE (Life Technologies) and stained with Coomassie-based SimplyBlue™ SafeStain (Life Technologies). Alternatively, for endogenous IFI16 interactions in differentiated monocytes and primary fibroblasts, immunoisolates were tryptically digested in-gel as previously described (Guise *et al*, 2012).

For LC-MS/MS, tryptic peptides were analyzed on an Ultimate 3000 nanoRSLC system (Dionex Corp., Sunnyvale, CA, USA) coupled online with an ESI-LTQ-Orbitrap Velos ETD mass spectrometer (ThermoFisher Scientific, San Jose, CA, USA). Reverse-phase chromatography was performed with mobile phase A 0.1% FA/0.1% acetic acid in water and mobile phase B 0.1% FA/0.1% acetic acid in 97% ACN. Peptides were separated over a 180-min gradient (4% B to 35% B) with 250 nl/min flow rate and analyzed by MS scans followed by data-dependent collision-induced dissociation (CID) MS/MS fragmentation of top 20 most abundant ions. The following parameters were used: FT preview scan disabled, waveform injection and dynamic exclusion enabled, automatic gain control target value of 1 × 10^6^ for MS and 1 × 10^4^ for ion trap MS/MS scan, max ion injection time of 300 ms for MS and 125 ms for MS/MS scan. For MS scan: *m/z* range of 350–1,700 and resolution of 60,000; for MS/MS spectra: minimum signal of 5,000, isolation width of 2.0, normalized collision energy of 30% and activation time of 10 ms.

### Database searching and peptide/protein identification

MS/MS data were first searched by SEQUEST (version 1.20) in Proteome Discoverer (version 1.3, ThermoFisher) using a database including human protein sequences (SwissProt, release 2010_11), common contaminants, and reversed sequences (21,569 total entries). SEQUEST parameters were as follows: full enzyme specificity for trypsin, ion mass tolerances of 10 ppm for precursor ions and 0.5 Da for fragment ions, fixed modification including cysteine carbamidomethylation, and dynamic modifications including methionine oxidation, serine, threonine, and tyrosine phosphorylation. Additional variable modifications of lysine acetylations, asparagine, and glutamine deamidation were included in a refining search by X!Tandem algorithm in Scaffold (version 3.6, Proteome Software Inc.), with the same parameters as above.

### Assessing interaction specificity with SAINT

Following protein identification (peptide and protein false-discovery rate < 1%, by Scaffold), the unweighted, label-free spectral counts for all identified proteins were exported from Scaffold to assess the interaction specificity using SAINT (Choi *et al*, 2011). Spectral count matrices were generated for each individual PYHIN bait and included the following information: prey gene name, prey accession number, prey amino acid length, and prey spectral counts. The required amino acid sequence lengths were retrieved from the UniProtKB database. An individual matrix contained the above information for all prey proteins identified in all replicates from GFP-only and both N- and C-terminally GFP-tagged PYHIN bait isolations (considered unique experiments). The same three GFP-only control isolations were used for each PYHIN protein SAINT analysis. For SAINT filtering of IFI16 interactions in THP-1 and HFF cells, two IgG control isolations from the appropriate cell type were used in addition to the three HEK293 GFP-only controls. Matrices were reformatted to SAINT-compatible input files and used for SAINT (ver. 2.3) probabilistic scoring using the following parameters: lowmode = 0, minfold = 1, and norm = 1 (Choi *et al*, 2012). SAINT-assigned individual probability scores for each bait–prey pair were averaged across replicates. Pairs with averaged probability scores ≥ 0.85 (for IFI16, IFIX, and AIM2) or ≥ 0.95 (for MNDA) were considered as specific, putative interactions. The same SAINT probability score filtering threshold of ≥ 0.85 was applied to endogenous IFI16 interactions in THP-1 and HFF cells to keep SAINT analyses comparable across all examined cell types. Using these averaged SAINT scores ensured that all candidates passing the thresholds were identified in both replicates. To increase the stringency of our analysis, only SAINT-filtered interactions with > 3 spectral counts for two replicates of the N- or C-terminally tagged PYHIN isolation for which that interaction passed SAINT were retained within the PYHIN interaction network.

### Hierarchical clustering

SAINT-filtered prey proteins from all PYHIN protein isolations were combined into a non-redundant list with the assigned, unweighted spectral counts from each isolation. Spectral counts were averaged across replicates, log_2_-transformed, and imported into Multiexperiment Viewer (MeV, version 4.9.0). Prey and bait proteins were then clustered using Pearson's correlation distance metric (average linkage).

### Functional PYHIN interaction network assembly

Interactions retained following SAINT and manual filtering (see above) for either N- or C-terminally tagged PYHIN isolations were combined into a single, comprehensive list for each individual PYHIN protein. If an interaction was found to be specific by SAINT in only one of the differentially tagged baits, that interaction additionally had to have been observed (i.e., averaged ≥ 1 spectral count per replicate) within the N- or C-terminally tagged isolation for which it did not pass SAINT to be considered a true interaction of that individual PYHIN protein. For complete list, the interactions successfully incorporated into the PYHIN family interactome refer to Supplementary Table S4. SAINT-filtered interactions which did not pass this criterium can be found in Supplementary Table S5. The remaining interactions were assembled into functional interaction networks using the Web-based STRING database (Szklarczyk *et al*, 2011). All default parameters, except for ‘text mining’, were enabled for assigning curated functional and physical protein interactions. Probability scores ≥ 0.7 (for IFIX and AIM2) or ≥ 0.9 (for IFI16 and MNDA) were used. STRING networks exported as xml files and loaded into Cytoscape (ver. 2.8.3) (Shannon *et al*, 2003; Smoot *et al*, 2011) for further network manipulation. As the putative interactions do not exist in the STRING database, a PYHIN interaction matrix was additionally generated in parallel, pairing each PYHIN bait with all corresponding SAINT-filtered preys. These matrices were also imported into Cytoscape, and all STRING networks and interaction matrices were merged to generate a comprehensive PYHIN protein family interaction network (Supplementary File S1). Each interaction is represented by nodes (bait or prey gene) connected with network edges. For simplicity, many interactions are not visualized in this way, and interaction is denoted by proximity to the respective PYHIN bait. All network genes were assigned GO full terms through the Cytoscape interface and color-coded according to their molecular functions. Genes were also given different shapes corresponding to the number of PYHIN proteins with which they specifically interact, as determined by SAINT. For the interaction network shown in Fig4A, nodes are colored according to their normalized spectral abundance factors (NSAF) (Weiss *et al*, 2010). In essence, NSAF is the spectral abundance of a given protein normalized for its amino acid length, expressed as a percentage of the sum of all included NSAF values.

### Immunocytochemistry (ICC) and fluorescence microscopy

For ICC experiments, sub-confluent cells were seeded on 13-mm glass cover slips. For studies performed in HEK293 cells, cover slips were pre-treated with 0.2 μg/cm^2^ poly-D-lysine. Following attachment, cells were treated with 1 μg/ml tetracycline for 24 h to induce expression of GFP-tagged PYHIN proteins. For all ICC experiments, cells were first fixed in 2% (v/v) paraformaldehyde (PFA) in phosphate-buffered saline (PBS) for 15 min and then permeabilized with 0.1% (v/v) Triton X in PBS for 15 min. For the following, 3–5-min washes were performed using PBS-T (0.2% (v/v) Tween-20 in PBS) between sequential steps. Next, cells blocked with 3% (w/v) BSA in PBS-T for 1 h. For immunostaining, cells were sequentially probed for 1 h with primary antibody in 3% (w/v) BSA in PBS-T, then incubated for 1 h with the appropriate secondary antibody conjugated to either Alexa488 or Alexa568 fluorophores (Life Technologies) in 3% (w/v) BSA in PBS-T. Nuclei were stained with 1 μg/ml DAPI in PBS-T for 10 min. All described steps were performed at room temperature. Stained cells were mounted with Aqua-Poly/Mount (Polysciences, Inc.) and visualized on a Leica SP5 confocal microscope (Leica Microsystems) using a 63× immersion oil objective.

### shRNA-mediated knockdown of PML and IFIX in HFFs

Lentivirus was produced in the HEK293T packaging cell line (ATCC #CRL-11268) by co-transfecting lentiviral packaging vectors psPAX2, pMD2.G-VSV-G, and pLK0.1 (containing target shRNA sequence) using Lipofectamine 2000. Cell supernatants containing lentivirus were collected at 40, 56, and 72 h post-transfection and filtered through a 0.45-μm membrane. Target HFF cells were transduced for three consecutive days with filtered lentivirus and selected for at least 1 week using 2 μg/ml puromycin (Invivogen) and then maintained thereafter in 500 ng/ml puromycin. Fibroblasts were assayed for target knockdown efficiency using either RT–qPCR or Western blotting.

### RNA isolation, reverse transcription, and qPCR

Total cellular RNA was extracted from cells with TRIzol reagent (Life Technologies) according to the manufacturer's instructions. mRNA then was reverse-transcribed via the poly-A tail using poly(dT) oligonucleotide primers and RETROscript reverse transcription kit (Life Technologies). For quantitative PCR experiments, the resulting cDNA was amplified using gene-specific primers and SYBR Green PCR master mix (Life Technologies) with an ABI 7900HT Fast Real-Time PCR System (Life Technologies). Relative quantification was performed by the ΔΔ*C*_T_ method, using *gapdh* or *β-actin* mRNA as an internal control. All primers used for qPCR quantification can be found in Supplementary Table S3.

### Recombinant proteins and electrophoresis mobility shift assays (EMSA)

IFIX-PY-GST (aa 1–100) and IFIX-HIN200-GST (aa 200–492) were inducibly expressed with IPTG in *E. coli* Rosetta (DE3) strain at 25°C for 5 h. GST fusion proteins were then purified with glutathione–Sepharose 4B (GE Healthcare) in PBS (10 mM Na_2_HPO_4_, 1.8 mM KH_2_PO_4_, 2.7 mM KCl, 140 mM NaCl) with 2 mM DTT and stored at −80°C prior to their use. For EMSA, 2 pmol recombinant protein and 20 fmol biotinylated ISD dsDNA were incubated in binding buffer (10 mM Tris, 50 mM KCl, 1 mM DTT, 50 μg/ml BSA, pH 7.5) at ambient temperature for 30 min. For competition assays, the mixtures were supplemented with unlabeled ISD, circular pcDNA3.1 plasmid, or *Bam*HI-linearized pcDNA3.1. All utilized dsDNA substrates were purified by QIAquick PCR Purification Kit (Qiagen, Valencia, CA, USA). After incubation, the mixtures were separated on a non-denaturing polyacrylamide gel and transferred to nylon membranes in 0.5× TBE buffer. Detection of biotinylated DNA was performed using the LightShift Chemiluminescent EMSA Kit (Pierce, Rockford, IL).

### Protein-Binding Microarray (PBMs)

PBM experiments were performed as in (Berger *et al*, 2006). Custom 60-mer single-stranded DNA microarray (Agilent) with approximately 44,000 features consisting of all possible 10-mer was made double-stranded using a universal primer. The microarray was incubated with 2 μg recombinant GST-IFIX-HIN200 protein in PBS, 2% (w/v) milk, 50 ng/μl salmon testes DNA (Sigma), and 0.2 μg/μl BSA at 20°C for 1 h. After extensive washes, the microarray was stained with Alexa-488-conjugated anti-GST antibody to visualize formation of DNA–protein interactions using a GSI Lumonics ScanArray 5000 scanner (Packard Instrument, Meriden, CT, USA). Next, raw intensity data were normalized and enrichment scores were calculated by the Universal PBM Analysis Suite developed by the Bulyk Lab (http://the_brain.bwh.harvard.edu/PBMAnalysisSuite/index.html). Using position weight matrices constructed by the ‘Seed-and-Wobble’ algorithm (Berger *et al*, 2006), DNA binding preferences were illustrated by the Web-based tool enoLOGOS (Workman *et al*, 2005).

### Chromatin immunoprecipitation

HEK293 cells expressing GFP-IFIX or GFP were infected with HSV-1 (17+) strain at MOI of 5. At 16 hpi cells were cross-linked with 1% paraformaldehyde (PFA) for 15 min, followed by quenching with 125 mM glycine. Cell lysates were extracted with lysis buffer (20 mM K-HEPES pH 7.4, 0.1 M sodium acetate, 2 mM MgCl_2_, 0.1% (v/v) Tween-20, 1 μM ZnCl_2_, 1 μM CaCl_2_, 0.6% Triton X-100, 200 mM NaCl, and 1/100 protease inhibitor cocktail (PIC, Sigma)) for 30 min on ice and bath-sonicated for 30 s, three times. GFP-IFIX or GFP was immunoaffinity isolated on magnetic beads conjugated with in-house GFP antibody for 2 h at 4°C. After extensive washing, the isolated protein–DNA complexes were eluted in SDS buffer. Cross-linking was reversed by overnight incubation at 65°C. DNA was purified with Qiaquick PCR Purification Kit (Qiagen), and viral DNA was detected by PCR using HSV-1 gene-specific primers.

### Data availability

The mass spectrometry proteomics data have been deposited to the ProteomeXchange Consortium (Vizcaino *et al*, 2014) via the PRIDE partner repository with the dataset identifier PXD001572 and 10.6019/PXD001572. The protein interactions have been submitted to the IMEx consortium through IntAct (Orchard *et al*, 2014) and assigned the identifier IM-23523.

## Supporting Information

Supplementary Tables S12

Review Process File
