# Supplementary material for: The functional interactome of PYHIN immune regulators reveals IFIX is a sensor of viral DNA
Source: Mol Syst Biol. 2015 Feb 9;11(2):787. doi: 10.15252/msb.20145808 (PMC4358659; doi:10.15252/msb.20145808)
Supplement: Supplementary file 1 [file msb0011-0787-sd1.pdf]

Supplementary Information Table of Contents

"The functional interactome of PYHIN immune regulators reveals IFIX is a sensor of viral DNA"

	Description	Pg
Supplementary Figure S1	Investigating the PYHIN proteins in HEK293 cells	2
Supplementary Figure S2	Validating cell-based tools for probing the PYHIN family interactome	3
Supplementary Figure S3	Efficiency of isolations for GFP-tagged AIM2 and MNDA	4
Supplementary Figure S4	Distribution of SAINT scores	5
Supplementary Figure S5	IFI16 interactome as a function of enrichment index	6
Supplementary Figure S6	IFIX interactome as a function of enrichment index	7
Supplementary Figure S7	MNDA interactome as a function of enrichment index	8
Supplementary Figure S8	AIM2 interactome as a function of enrichment index	9
Supplementary Figure S9	Stability of PYHIN family interactions	10
Supplementary Figure S10	Co-localization of IFIX-GFP and IFI16-GFP with VACV in the cytoplasm	11
Supplementary Figure S11	Localization of IFIX-GFP during HSV-1 infection	12
Supplementary Figure S12	Interactions of IFIX-GFP during HSV-1 infection	13
Legends	Supplementary Table Legends	14
Supplementary Table S1	Primers for N- or C-terminally GFP tagging of pyhin genes	15
Supplementary Table S2	Primers for generating inducible HEK293 cell lines	16
Supplementary Table S3	Primers used for Reverse Transcription-Quantitative PCR	17
Supplementary Table S4	SAINT-filtered interactions observed in both N- and C-terminal isolations	NA
Supplementary Table S5	SAINT-filtered interactions unique to either N- or C-terminal isolations	NA
Supplementary Table S6	Non-specific interactions excluded from the PYHIN family interactome	NA
Supplementary Table S7	Nuclear IFI16 Interactions in Differentiated THP-1 Monocytes	NA
Supplementary Table S8	IFI16 Interactions in Human Foreskin Fibroblasts	NA
Supplementary Table S9	Primers for generating GST-tagged IFIXα1 truncations	18
Supplementary Table S10	Oligonucleotide sequences of VACV 70mer and ISD	19
Supplementary Table S11	Primers used for detection of HSV-1 genomic DNA	20
Supplementary Table S12	IFIX Interactions following HSV-1 infection	NA

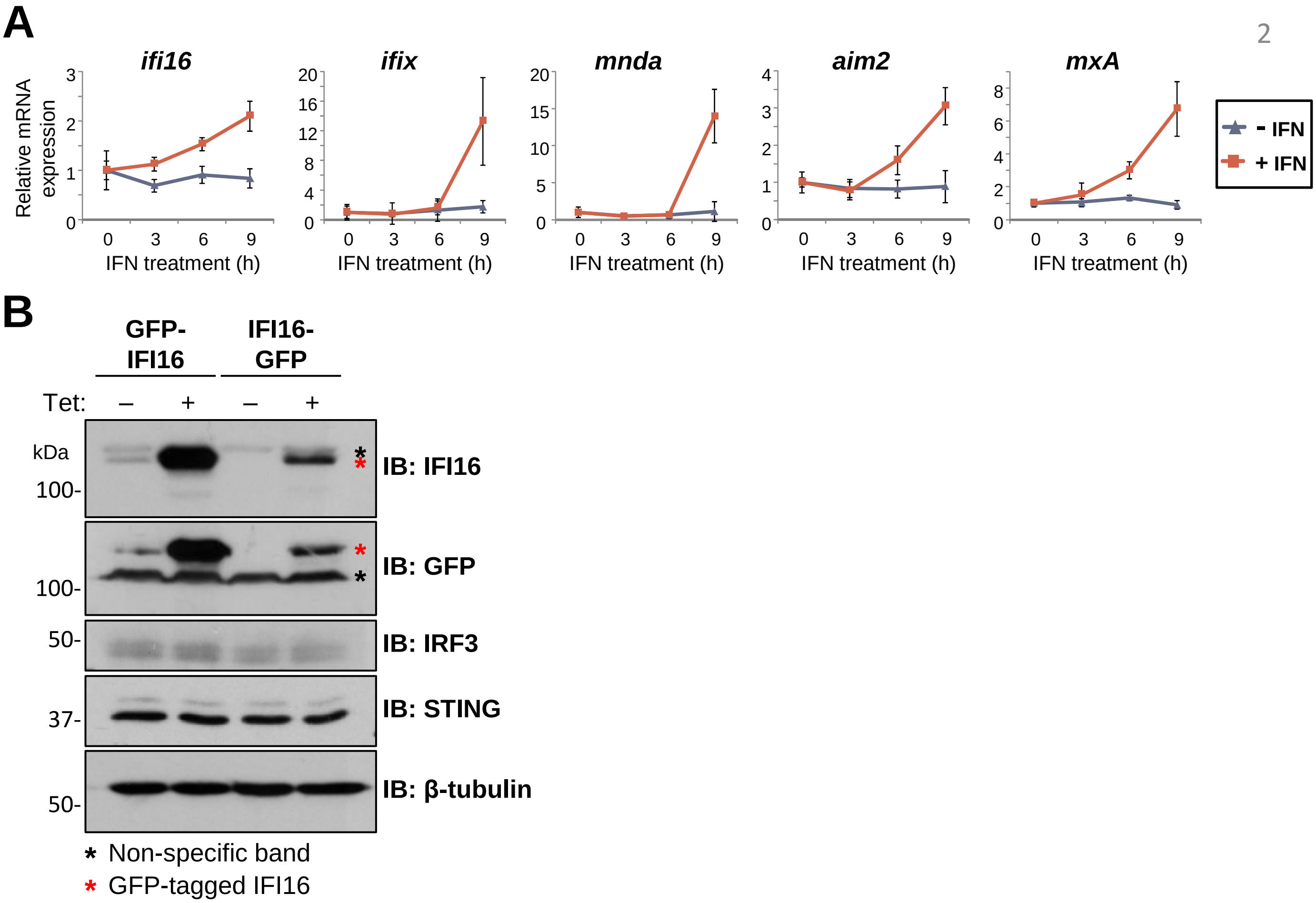

Figure S1. Investigating the PYHIN proteins in HEK293 cells.
(A) RNA was collected from HEK293 cells treated with IFN-β (1000U/mL) for the indicated amount of time. RT-qPCR analysis was performed to measure the induction of *pyhin* family genes. mRNA levels are normalized to cellular *gapdh* levels. The basal expression levels in wild type HEK293 cells for *ifi16, ifix, mnda, aim2*, and *mxA* relative to *gapdh* were 2.9E-6, 2.1E-5, 9.7E-5, 5.7E-7, and 2E-6, corresponding to raw Ct values of ~29, 20, 22, 30, and 28, respectively. Mean values ± SD (n=3) are shown. **(B)** Lysates from uninduced or tetracycline-induced HEK293 cell lines with either GFP-IFI16 or IFI16-GFP transgenes were probed by Western blot for the presence of immune regulators STING and IRF3 using protein-specific antibodies.

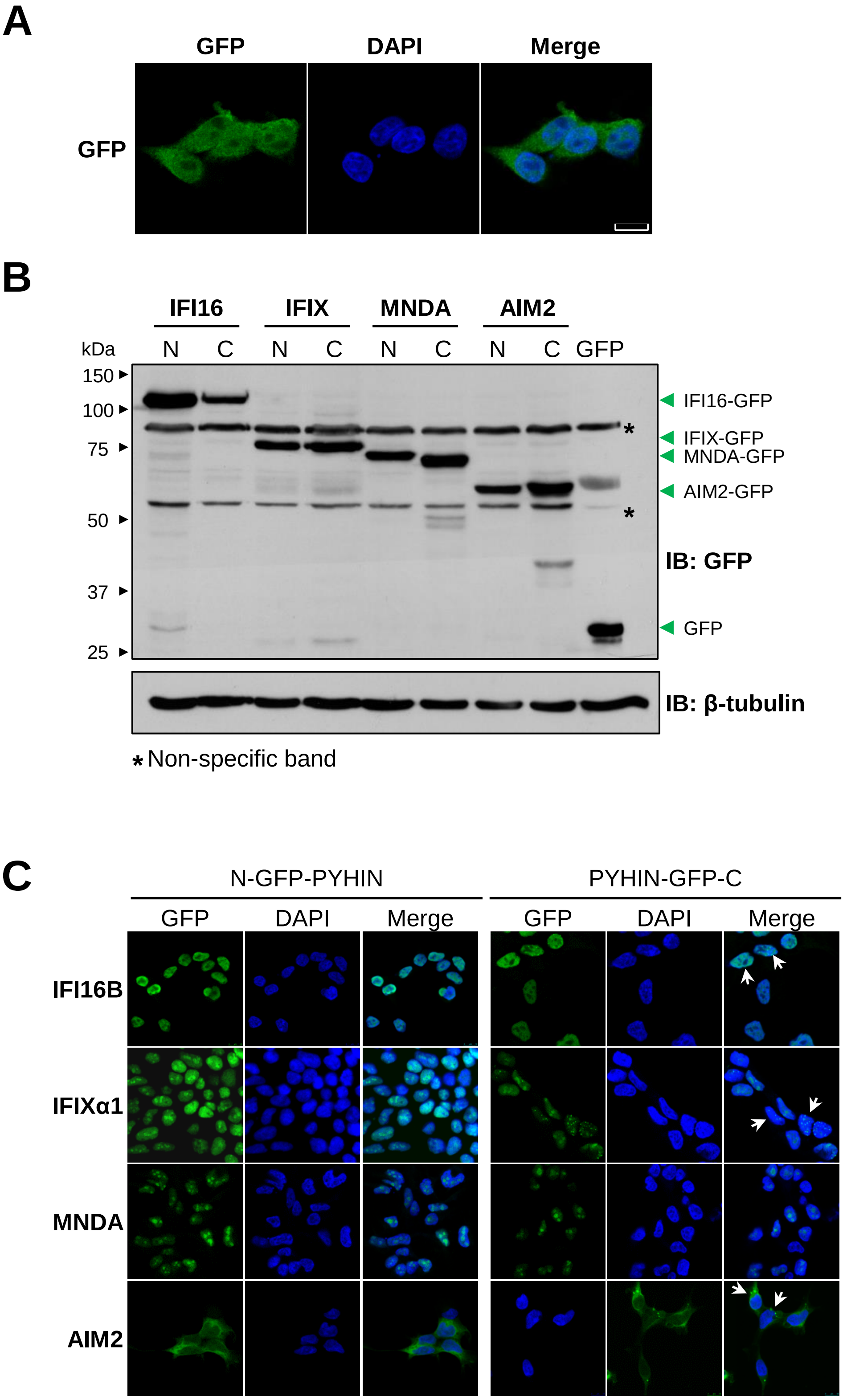

Figure S2. Validating cell-based tools for probing the PYHIN family interactome.
(A) Immunofluorescence confocal microscopy was used to image control HEK293 cell line inducibly expressing GFP only. Scale bar, 10 µm. **(B)** Lysates from all 8 HEK293 cell lines induced to express N- or C-terminally GFP-tagged PYHIN transgenes were assessed Western blots. All GFP-tagged proteins were detected using GFP-specific antibody and are indicated by green arrows. **(C)** Immunofluorescence confocal microscopy was used to quantify the frequency of PYHIN punctate structures observed for the differentially tagged PYHIN proteins. Quantification of the distributions are provided in Figure 1. White arrows indicate examples of cells that were scored as possessing punctate PYHIN structures.

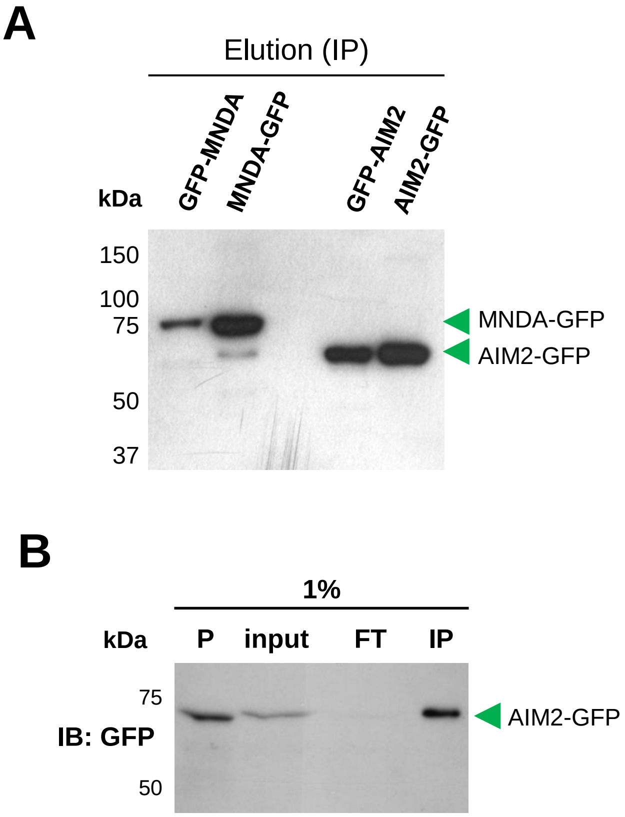

Figure S3. Efficiency of isolations for GFP-tagged AIM2 and MNDA.
(A) Original Western Blots (modified versions shown in Fig. 1F) from the samples ran for mass spectrometry for assessing isolation efficiency of N- and C-terminally GFP-tagged MNDA and AIM2. Equal proportions (1%) of the elution, flow-through and pellet fractions were loaded and the blots were exposed simultaneously (20 sec exposure). Green arrows indicate the GFP-tagged PYHIN proteins. **(B)** Additional example of Western blot demonstrating AIM2-GFP isolation efficiency.

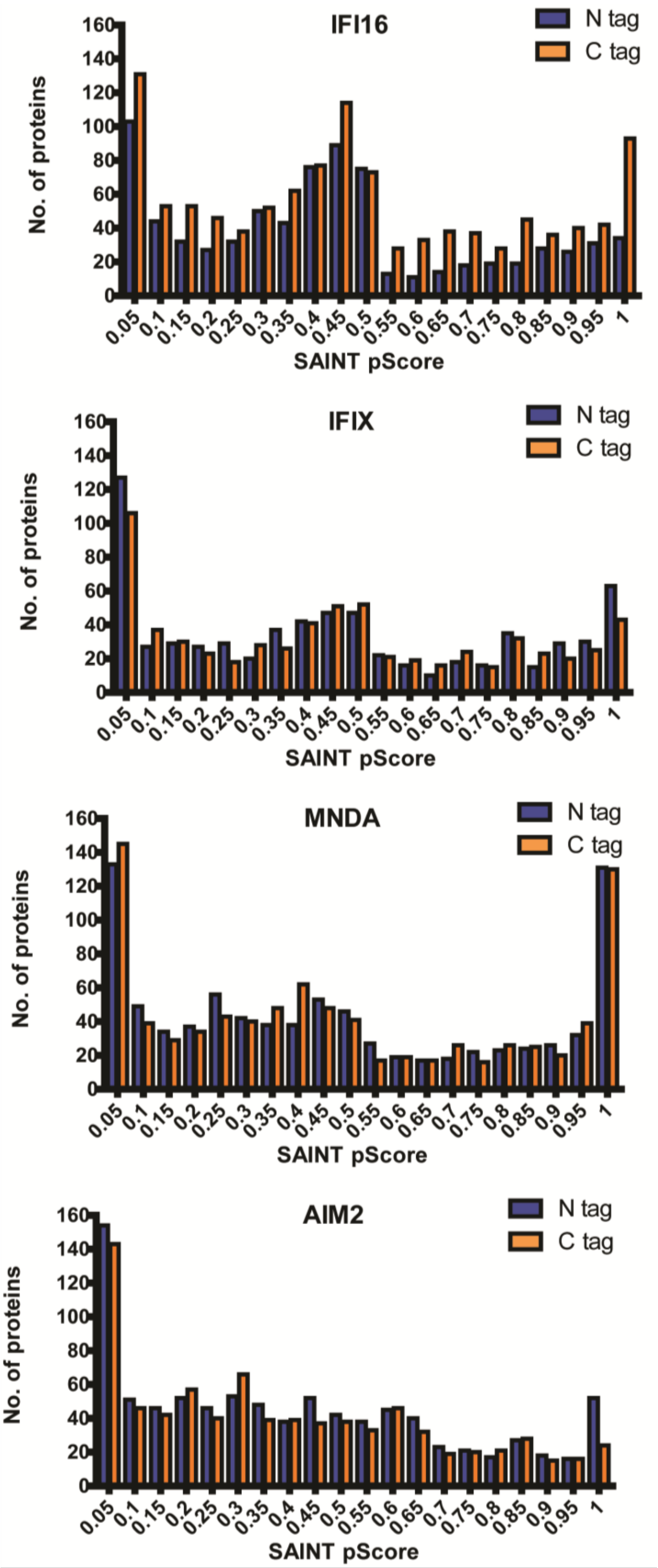

Figure S4. Distribution of SAINT scores.
The distribution of averaged SAINT scores (IFI16, IFIX, MNDA n=2; AIM2 n=3) for all proteins co-isolated with N- (blue) or C- (orange) terminally GFP-tagged PYHIN proteins. Scores are binned in 0.05 increments.

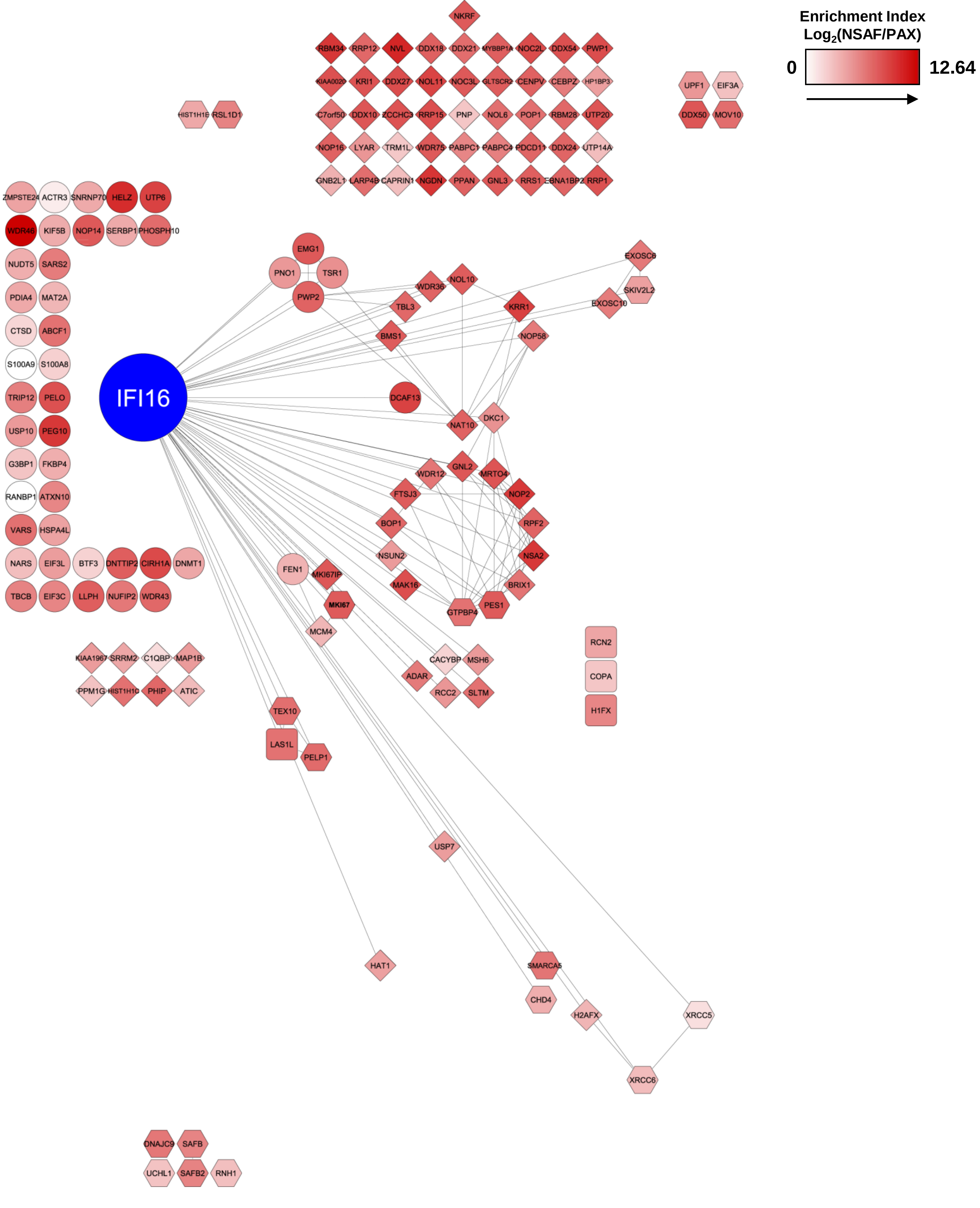

Figure S5. IFI16 interactome as a function of enrichment index. Interaction candidates of IFI16 with node color gradient representing increasing relative IP abundance versus whole cell (NSAF/PAX); see Materials and Methods). Positions and shapes of nodes are conserved from Figure 3.

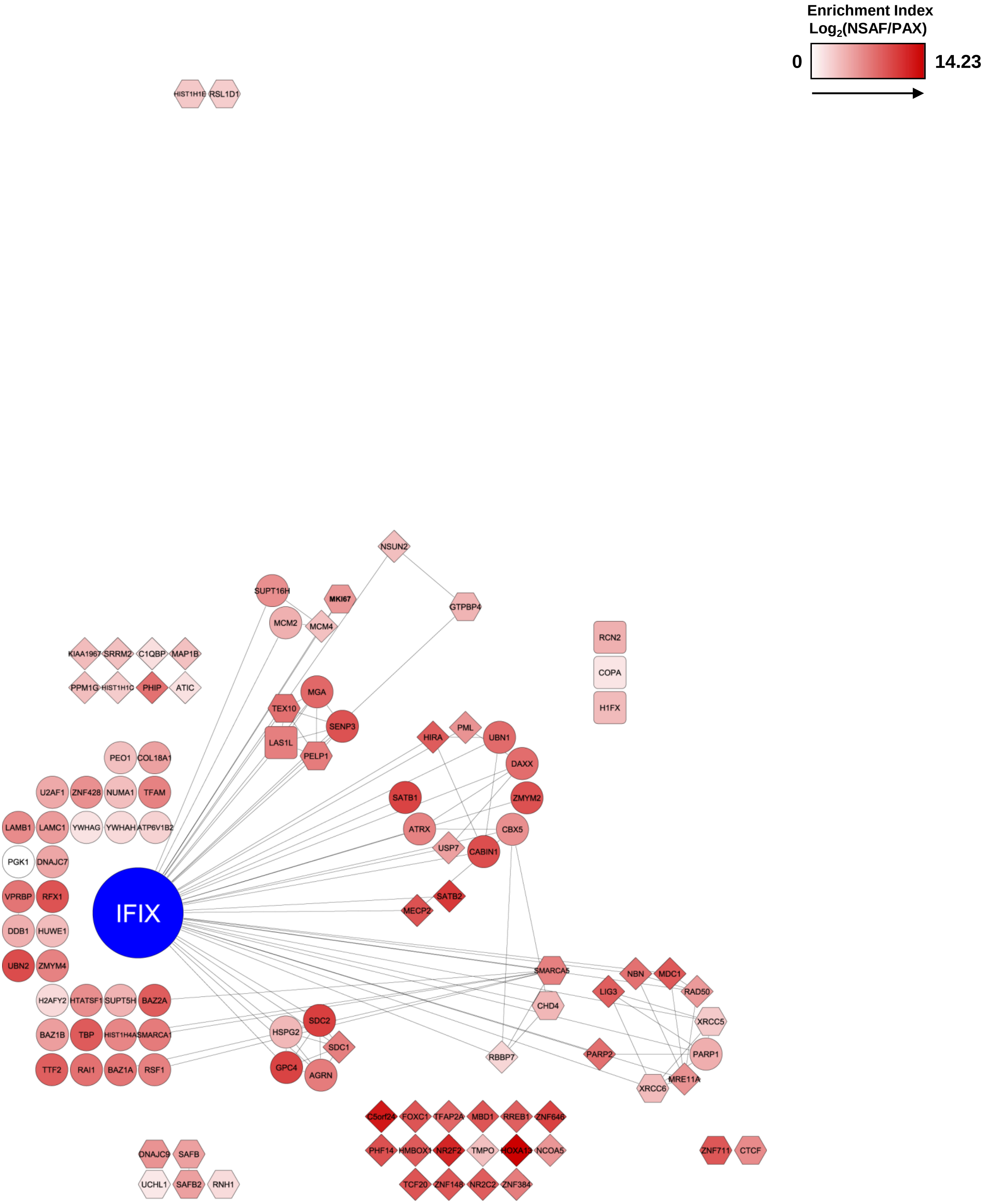

Figure S6. IFIX interactome as a function of enrichment index. Interaction candidates of IFI1X with node color gradient representing increasing relative IP abundance versus whole cell (NSAF/PAX); see Materials and Methods). Positions and shapes of nodes are conserved from Figure 3.

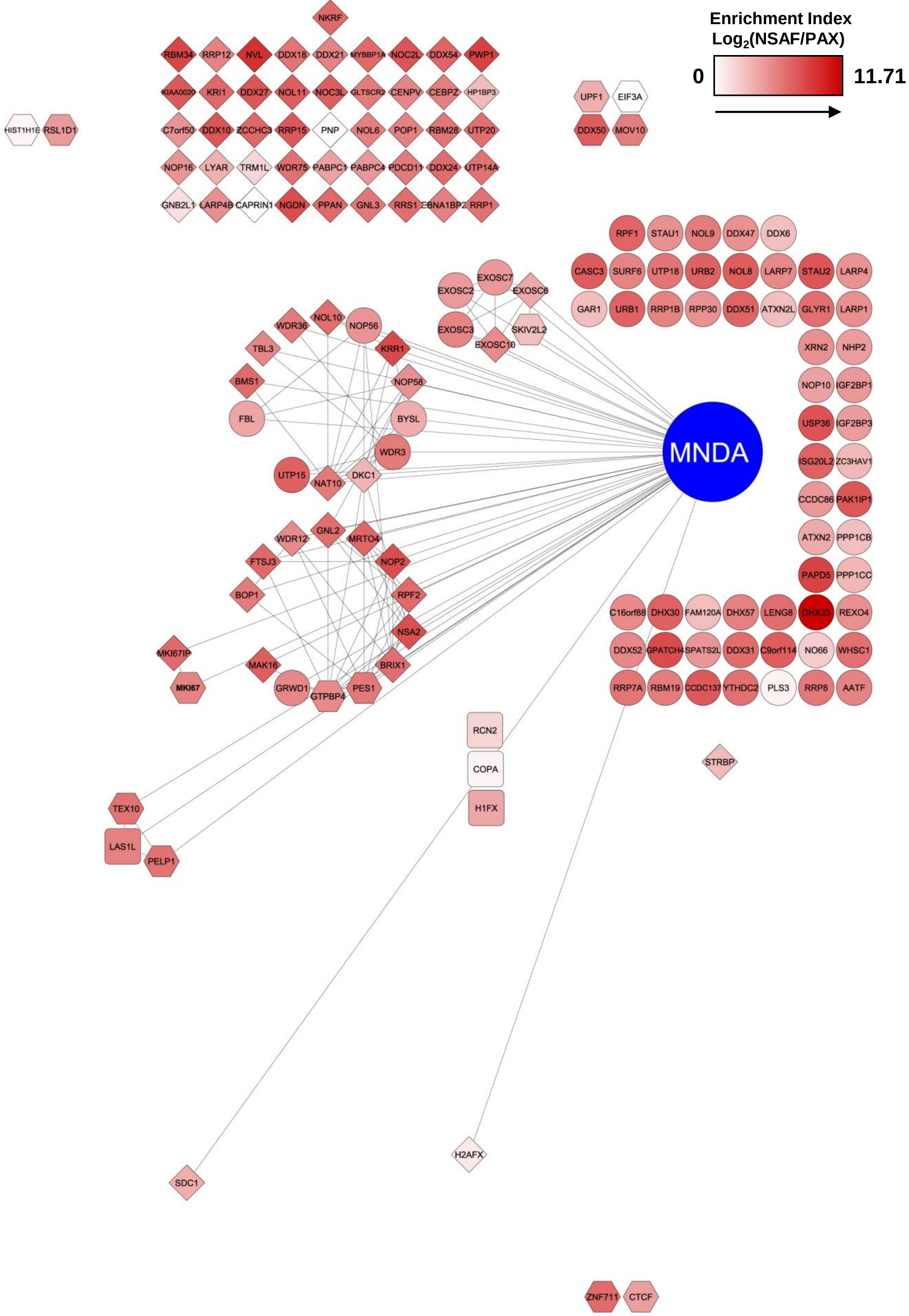

Figure S7. MNDA interactome as a function of enrichment index. Interaction candidates of MNDA with node color gradient representing increasing relative IP abundance versus whole cell (NSAF/PAX); see Materials and Methods). Positions and shapes of nodes are conserved from Figure 3.

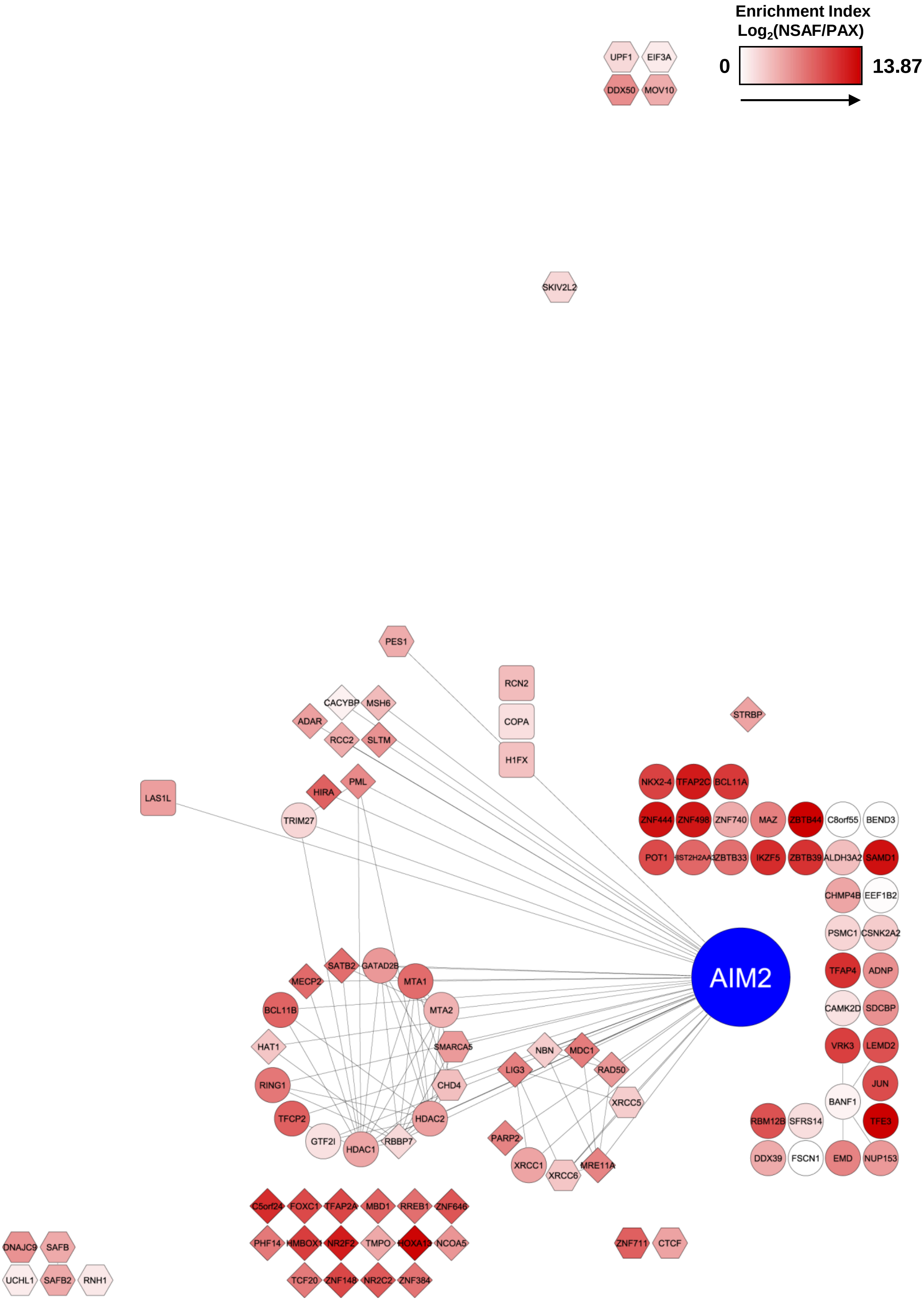

Figure S8. AIM2 interactome as a function of enrichment index. Interaction candidates of AIM2 with node color gradient representing increasing relative IP abundance versus whole cell (NSAF/PAX); see Materials and Methods). Positions and shapes of nodes are conserved from Figure 3.

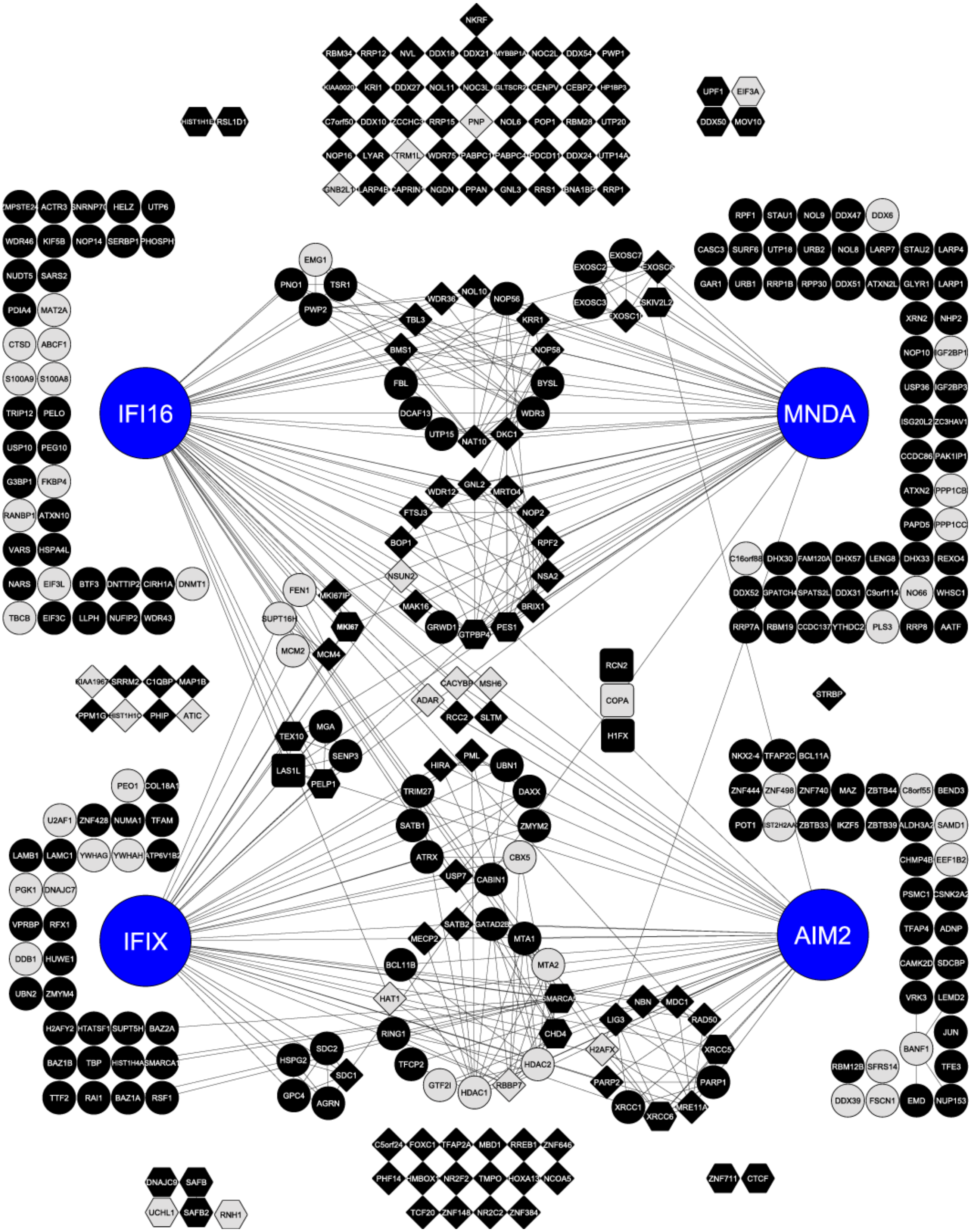

Figure S9. Stability of PYHIN family interactions. PYHIN protein interaction network from Fig 3 has been re-colored to indicate the 60 interactions (grey nodes) that no longer pass the SAINT scoring thresholds (see Materials and Methods) after including three additional controls from the Crapome server in the analysis. The Crapome database controls (CC173, CC175, and CC177) were selected to match the cell type (HEK293), affinity tag (GFP), support (magnetic beads), and instrument (Orbitrap Velos) of our current experimental design.

A Co-localization of IFIX-GFP with viral DNA in the cytoplasm

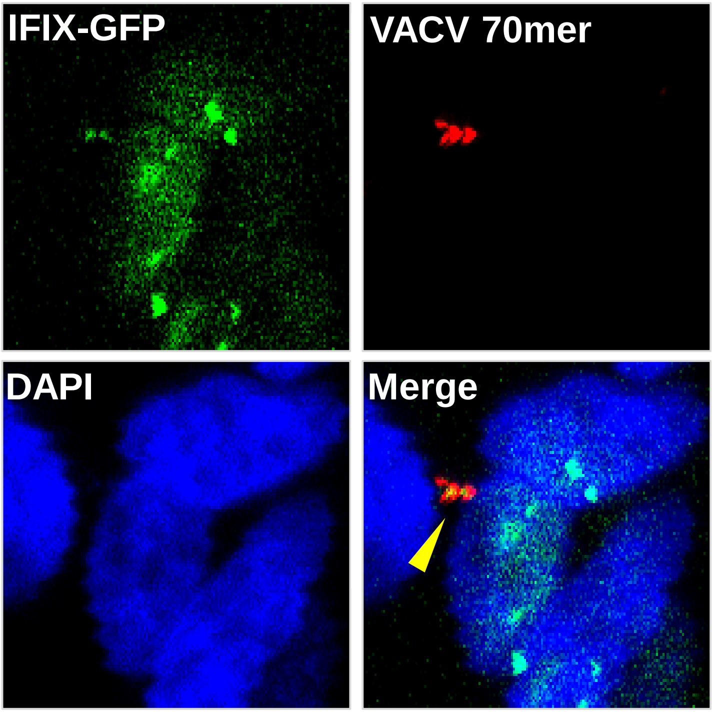

B Co-localization of IFI16-GFP with viral DNA in the cytoplasm

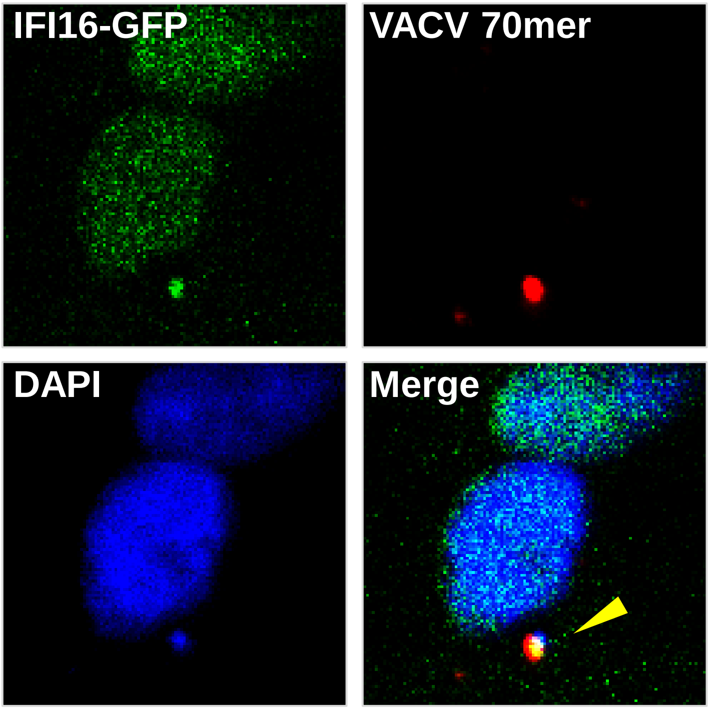

Figure S10. Co-localization of IFIX-GFP and IFI16-GFP with VACV in the cytoplasm. (A) Immunofluorescence confocal microscopy image (63x objective) showing co-localization of IFIX-GFP and transfected Cy3-VACV 70mer in HEK293 cells (yellow arrows). (B) Confocal microscopy image (63x objective) showing co-localization of IFI16-GFP with transfected Cy3-VACV 70mer in HEK293 cells (yellow arrows).

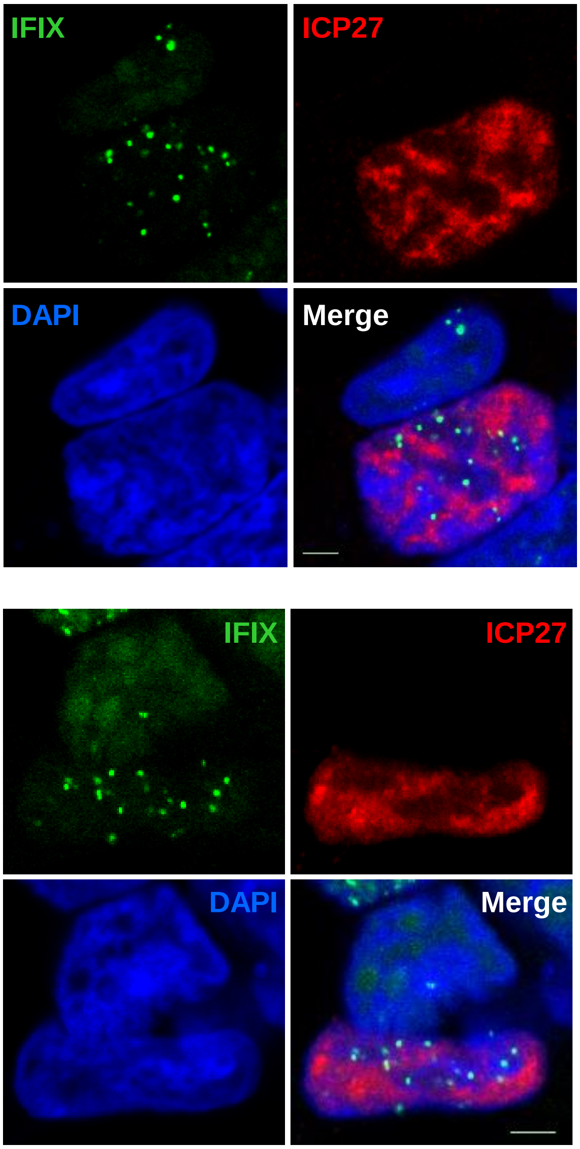

Figure S11. Localization of IFIX-GFP during HSV-1 infection. Immunofluorescence confocal microscopy images (63x objective) illustrating additional representative images of IFIX-GFP localization in HEK293 cells at 4 hours post infection with HSV-1 (MOI = 1). The viral protein ICP27 was used as a marker for infection and to facilitate the direct comparison of IFIX localization in uninfected and infected cells.

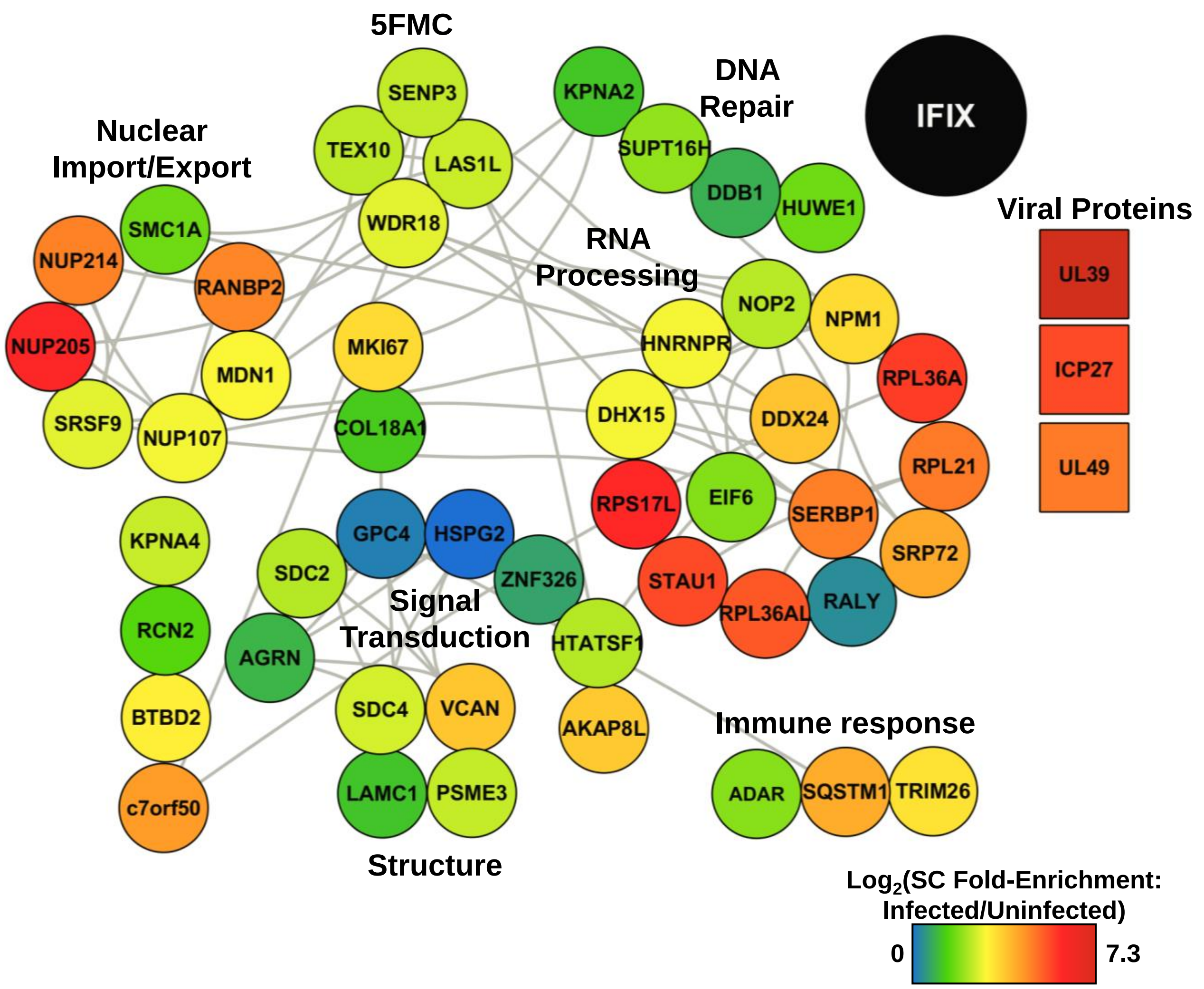

Figure S12. Interactions of IFIX-GFP during HSV-1 infection. IFIX-GFP was immunoaffinity purified (N=2) from HEK293 cells at 4 hours post infection with HSV-1 (MOI = 5), in parallel with control GFP (N=2). Protein interactions that passed SAINT filtering (>0.85) are illustrated. The Log_2 fold-enrichment of these interactions compared to those in uninfected cells is illustrated with colors. Edges represent physical interactions between nodes based on STRING analysis of the SAINT-filtered prey proteins.

Supplemental Excel Table Legends

Supplementary Table S1. Primers for N- or C-terminally GFP tagging of *pyhin* genes. Legend provided with the table below.

Supplementary Table S2. Primers for generating inducible HEK293 cell lines. Legend provided with the table below.

Supplementary Table S3. Primers used for Reverse Transcription-Quantitative PCR. Legend provided with the table below.

Supplementary Table S4. SAINT-filtered Interactions Observed in Both N- and C-terminal Isolations. Protein interaction candidates that were classified as specific after SAINT analysis in both N- and C-terminal isolations of IFIX, IFI16, MNDA, or AIM2. For each protein, the following information is provided: UniProt accession, gene symbol, protein length in amino acids, description, average SAINT score (Prob) and spectral counts (SC) for each PYHIN IP and GFP IP controls.

Supplementary Table S5. SAINT-filtered Interactions Unique to Either N- or C-terminal Isolations. Protein interaction candidates that were classified as specific after SAINT analysis for IFIX, IFI16, MNDA or AIM2 isolations, but were unique to either the N- and C-terminal isolations. This subset of proteins was excluded from the interactome network analysis. For each protein, the following information is provided: UniProt accession, gene symbol, protein length in amino acids, description, average SAINT score (Prob) and spectral counts (SC) for each PYHIN IP and GFP IP controls.

Supplementary Table S6. Non-specific Interactions Excluded from the PYHIN Family Interactome. Proteins that were classified as non-specific after SAINT analysis. For each protein, the following information is provided: UniProt accession, gene symbol, description, protein length in amino acids, average SAINT score (Prob) and spectral counts (SC) for each PYHIN IP and GFP IP controls.

Supplementary Table S7. Nuclear IFI16 Interactions in Differentiated THP-1 Monocotyes. For each protein, the following information is provided: UniProt accession, gene symbol, description, protein length in amino acids, average SAINT score (Prob) and spectral counts (SC) for each PYHIN IP and GFP IP controls.

Supplementary Table S8. IFI16 Interactions in Human Foreskin Fibroblasts. For each protein, the following information is provided: UniProt accession, gene symbol, description, protein length in amino acids, average SAINT score (Prob) and spectral counts (SC) for each PYHIN IP and GFP IP controls.

Supplementary Table S9. Primers for generating GST-tagged IFIXα1 truncations. Legend provided with the table below.

Supplementary Table S10. Oligonucleotide sequences of VACV 70mer and ISD. Legend provided with the table below.

Supplementary Table S11. Primers used for detection of HSV-1 genomic DNA. Legend provided with the table below.

Supplementary Table S12. IFIX Interactions during HSV-1 Infection. For each protein, the following information is provided: UniProt accession, gene symbol, description, protein length in amino acids, average SAINT score (Prob) and spectral counts (SC) for each PYHIN IP and GFP IP controls.

Primer	Sequence
**IFI16b**-F-XhoI	5'- GCCG CTCGAG **ATGGGAAAAAAATACAAGAACATTG**
**IFI16b-Stop**-R-BamHI	5'- GCGC GGATCC **TTACGGAAGAAAAAGTCTGGTGAAGTTTC**
**IFI16b-NoStop**-R-BamHI	5'- GCGC GGATCC **CGGAAGAAAAAGTCTGGTGAAGTTTC**
**IFIXα1**-F-XhoI	5'- GCGC CTCGAG **ATGGCAAATAACTACAAGAAAATTG**
**IFIXα1-NoStop**-R-BamHI	5'- GCGC GGATCC **CGAGGAACTGCTGGATGGCGGTT**
**MNDA**-F-XhoI	5'- GCCG CTCGAG **ATGGTGAATGAATACAAGAAAATTCT**
**MNDA-Stop**-R-BamHI	5'- GCGC GGATCC **TTACGATTAACATTCATTGGTCCTTCCTT**
**MNDA-NoStop**-BamHI	5'- GCGC GGATCC **CGATTAACATTCATTGGTCCTTCCTT**
**AIM2**-F-XhoI	5'- GCCG CTCGAG **ATGGAGAGTAAATACAAGGAGATAC**
**AIM2-Stop**-R-BamHI	5'- GCGC GGATCC **TTACGTGTTTTTTTTTTGGCCTTAATAACCTT**
**AIM2-NoStop**-R-BamHI	5'- GCGC GGATCC **CGTGTTTTTTTTTTGGCCTTAATAACCTT**

Supplementary Table S1. Primers for N- or C-terminally GFP tagging of *pyhin* genes. Primers for subcloning *pyhin* genes into pEGFP-C3 or pEGFP-N1 expression vectors to generate N- and C-terminally tagged constructs, respectively (Figure 1).

Primer	Sequence
**GFP**-F-BamHI	5’- GCGC GGATCC **ATGGTGAGCAAGGGCGAGG**
**GFP-Stop**-R-XhoI	5’- CCGCCG CTCGAG **TTACTTGTACAGCTCGTCCATG**
**GFP**-F-HindIII	5’- CCCC AAGCTT **ATGGTGAGCAAGGGCGAGG**
**IFI16b-Stop**-R-SalI	5’- ACGC GTCGAC **TTAGAAGAAAAAGTCTGGTG**
**IFIXα1-Stop-**R-XhoI	5’- GCCG CTCGAG **TTAAGGAACTGCTGGATGGCGG**
**MNDA-Stop**-R-BglII	5’- GCGC AGATCT **TTACGATTAACATTCATTGGTCCTTCCTT**
**AIM2-Stop**-R-BamHI	5’- GCGC GGATCC **TTACGTGTTTTTTTTTTGGCCTTAATAACCTT**
**IFI16b**-F-KpnI	5’- GCGG GGTACC **ATGGGAAAAAAATACAAGAACATT**

Supplementary Table S2. Primers for generating inducible HEK293 cell lines. Primers for subcloning GFP and N- and C-terminally GFP-tagged pyhin genes into pcDNA5/FRT/TO for generating inducible HEK293 cell lines (Figure 1B) are shown.

Primer	Sequence	Figure
qPCR-GAPDH-F	5'- CGACAGTCAGCCGCATCTTCTTT	S1A, S1B, 6E
qPCR-GAPDH-R	5'- GGCAACAATATCCACTTTACCAGAG	S1A, S1B, 6E
qPCR-IFI16-F	5'- GCTTGAAGACCTGGCTGAAA	S1A, S1B
qPCR-IFI16-R	5'- GAGGGTGCAGGTGAAGTAGC	S1A, S1B
qPCR-IFIX-F	5'- GCAACCGTCTCACAGCTAAA	S1A, S1B, 5E
qPCR-IFIX-R	5'- CGAGTCTGCTCTTTGGACATC	S1A, S1B, 5E
qPCR-MNDA-F	5'- CCACTACCCCAGACCTCATC	S1A, S1B
qPCR-MNDA-R	5'- TGGGGAACATTTCTTCTTGC	S1A, S1B
qPCR-AIM2-F	5'- TTTGGCAAAACGTCTTCAGG	S1A, S1B
qPCR-AIM2-R	5'- TGCAGCAGGACTCATTTCAG	S1A, S1B
qPCR-β-actin-F	5'- TCCTCCTGAGCGCAAGTACTC	5E
qPCR-β-actin-R	5'- CGGACTCGTCATACTCCTGCT	5E
qPCR-IFN-β-F	5'- GAAGCCTTTGCTCTGGCACAA	6E
qPCR-IFN-β-R	5'- CCTCCCATTCAATTGCCACA	6E

Supplementary Table S3. Primers used for Reverse Transcription-Quantitative PCR. Primers used in for quantification of mRNA transcript abundances in the indicated RT-qPCR experiments are shown.

Primer	Sequence
**GST-IFIXα1-PY**-F-BamHI	5’- GCGC GGATCC **GCAAATAACTACAAGAAAATTG**
**GST-IFIXα1-PYStop**-R-XhoI	5’- GCCG CTCGAG **TTATTTGACTGGAATGGATTCAATT**
**GST- IFIXα1-HIN200-**F-BamHI	5’- GCGC GGATCC **AACCGTCACGCAACTGCCAG**
**GST- IFIXα1-HIN200Stop-**R-XhoI	5’- GCCG CTCGAG **TTAAGGAACTGCTGGATGGCGG**

Supplementary Table S9. Primers for generating GST-tagged IFIXα1 truncations. Primers for cloning GST-IFIX-PY (aa 1-99) and GST-IFIX-HIN200 (aa 200-492) to be expressed from pGET4T-1 are shown. Purified recombinant proteins were used for the EMSA assays shown in Figure 6A-B.

Primer	Sequence
ISD-F	5’- TACAGATCTACTAGTGATCTATGACTGATCTGTACATGATCTACA
ISD-R	5’- TGTAGATCATGTACAGATCAGTCATAGATCACTAGTAGATCTGTA
VACV70mer-F	5’- CCATCAGAAAGAGGTTTAATATTTTTGTGAGACCATCGAAGAGAGAAAG AGATAAAACTTTTTTACGACT
VACV70mer-R	5’- AGTCGTAAAAAAGTTTTATCTCTTTCTCTCTTCGATGGTCTCACAAAAATA TTAAACCTCTTTCTGATGG

Supplementary Table S10. Oligonucleotide sequences of VACV 70mer and ISD. The above oligonucleotide pairs were annealed and used for various experiments. ISD was used for the EMSA assays shown in Figure 6A-B. VACV 70mer was used in cell assays shown in Figure 6D-E.

Primer	Sequence
HSV-1-RL2-F	5’- CTGTCGCCTTACGTGAACAA
HSV-1-RL2-R	5’- CATCCAGAGGCTGTTCCACT
HSV-1-UL30-F	5’- AGCGAATTCGAGATGCTGTT
HSV-1-UL30-R	5’- CCTTTATCTTGCTGCGCTTC
HSV-1-US6-F	5’- AGCAGGGGTTAGGGAGTTGT
HSV-1-US6-R	5’- GCATCCACCAAGGCATATTT

Supplementary Table S11. Primers used for detection of HSV-1 genomic DNA. Primers used to detect the presence of HSV-1 genomic DNA following chromatin immunopurification of GFP or GFP-IFIX (Figure 6F) are shown.
