# Supplementary material for: The functional interactome of PYHIN immune regulators reveals IFIX is a sensor of viral DNA
Source: Mol Syst Biol. 2015 Feb 9;11(2):787. doi: 10.15252/msb.20145808 (PMC4358659; doi:10.15252/msb.20145808)
Supplement: Supplementary file 9 [file msb0011-0787-sd9.pdf]

The functional interactome of PYHIN immune regulators reveals IFIX is a sensor of viral DNA

Benjamin A. Diner, Tuo Li, Todd M. Greco, Marni S. Crow, John Fuesler, Jennifer Wang, Ileana M. Cristea

Corresponding author: Ileana M. Cristea, Princeton University

Review timeline:

Submission date:	01 April 2014
Editorial Decision:	28 May 2014
New submission:	13 October 2014
Editorial Decision:	01 December 2014
Revision received:	10 December 2014
Accepted:	23 December 2014

Editor: Thomas Lemberger

Transaction Report:

(Note: With the exception of the correction of typographical or spelling errors that could be a source of ambiguity, letters and reports are not edited. The original formatting of letters and referee reports may not be reflected in this compilation.)

1st Editorial Decision 28 May 2014

Thank you again for submitting your work to Molecular Systems Biology. We have now finally heard back from the three referees whom we asked to evaluate your manuscript. Reviewer 3 was asked to only evaluate the technical aspects of the proteomics analysis. Reviewer #1 is an expert in innate immunity and reviewer #2 is an expert in proteomics and signaling. As you will see from the ports below, the referees raise substantial concerns on your work, which, I am afraid to say, preclude its publication in Molecular Systems Biology.

While the reviewers recognize the technical quality of the AP-MS analysis, both reviewer #1 and #2 raise significant concerns with regard to the biological insights gained by the analysis. Thus, the choice of HEK293 cells as the experimental system and the use of conditions without differential exposure to DNA seem to limit the relevance of the interaction set for innate immune functions. Additional concerns refer to the fact that the follow-up investigations of IFIX remain relatively independent from the preceding proteomics analysis while the finding of IFIX as a foreign DNA sensor is not completely unexpected given the activities of other family members. As such, both reviewer #1 and #2 feel that the study remains of relatively specialized nature and would not recommend publication in Molecular Systems Biology.

Overall, given the critiques expressed by the reviewers and the low level of suport for publication in Molecular Systems Biology, I see no other choice than to return the manuscript with the message that we cannot offer to publish it. I am sorry not to be able to bring better news on this occasion. In any case, thank you for the opportunity to examine your work and I hope that the points raised in the reports will prove useful to you.

© European Molecular Biology Organization

Reviewer #1:

This paper does a proteomic analysis of proteins interacting with human PYHIN (HIN-200) proteins in HEK293 cells. A large number of novel interactions were found which have a surprising degree of specificity for the different proteins, suggesting there are not many generic HIN or PYD interacting proteins which bind all family members. The authors' approach of tagging both N and C terminus seems to have yielded a reliable indication of interaction. The paper goes on to further characterize IFIX as a nuclear protein with a nuclear export sequence that binds transfected DNA in the cytosol, viral DNA in the nucleus, and associates with PML. IFIX is a protein little studied in the literature, and this work makes a useful contribution in this regard. It is not unexpected that IFIX will bind to foreign DNA and mediate responses to viruses, as this is found in other family members.

I think the main improvement which could readily be made to this paper is in the presentation of what is being studied and the system used. Interactions of these proteins are analyzed in HEK293 cells, without infection or introduction of DNA. The lack of DNA stimulus, as well as the lack of convincing role for these proteins in HEK293 cells, means that interactions with proteins related to foreign DNA responses may not be captured. However, there is a wealth of prior literature implicating PYHIN proteins in other processes such as senescence, apoptosis, cell cycle regulation, DNA damage recognition which may be part of normal homeostasis. The paper could better emphasize the use of these cells to define interactions of PYHIN proteins with widely expressed proteins, related to the broad range of suggested functions. The beginning of the results section tries to convince the reader that HEK293 cells are a good cell type in which to be analyzing interactions of PYHIN proteins related to their innate immune function. I am not convinced, for reasons given below, but I do I think that some useful results have been obtained.

Specific comments

1. A test of the assertion that HEK293 cells are a good model to assess interactions of PYHIN factors is the fact that the approach used cannot expose the known role of AIM2, in initiating an inflammasome response. HEK293 are used a lot for reconstituting innate immune pathways, and they are useful because they lack most of these receptor systems. The results section says "We and others have previously demonstrated IFI16- and AIM2-dependent immune functions" in HEK293. I have not checked all these papers but the cited Fernandes-Alnemri paper shows AIM2 dependent functions in these cells only by transfecting in all the components of the system which are lacking:- AIM2, ASC, caspase-1 and IL-1b. This is hardly a recommendation for this cell line as a model of AIM2 responses. The whole opening of the paper seeks to validate HEK293 as a cell line where PYHIN proteins are normally active. This is not convincing, and an alternative approach is needed. I do not believe that this cell line has significant levels of expression of the factors claimed. MNDA for instance is exquisitely myeloid restricted, as shown in online array data (GNF symatlas). A reference which looks like it is included to support HEK expression of MNDA does not use HEK cells (Fotouhi-Ardakani). Expression data shown in the paper is real time PCR, normalized so that no impression can be gained of the absolute level of expression. I recommend abandoning the attempt to convince readers that HEK293 cells normally have functional levels of all these PYHIN proteins. But if this expression data is to be shown, it should be shown as just the ratio of gene expression to a standard housekeeping control gene, so that an impression can be gained of absolute level. Samples coming up at late cycle number are of little consequence. It would also have been better to compare expression to peripheral blood cells, which really do express these factors. Even then, if you wanted to convince us that these factors are expressed, there are very good antibodies for AIM2 and IFI16.

2. Similarly for Figure 5C and Sup Fig 1A, please don't normalize PCR results to one of the samples, but show data normalized to gapdh control for each sample. Unnecessary normalization loses information.

3. Although some of the protein:protein interactions described here may be relevant in the response to foreign DNA, it should be noted that the pull downs were done from cells not exposed to such

© European Molecular Biology Organization

DNA. Thus these interactions might be more relevant to other homeostatic roles of these proteins. This may provide a better focus for the paper.

4. I think conclusions about PYHINs associating with DNA damage factors like Ku proteins are problematic. If you pull down HEK cell extracts with DNA, the dominant proteins found are Ku. Pull-downs of PYHINs will have associated DNA, and this will bring down DNA damage proteins without them necessarily being in any direct association with the PYHIN. This is in fact a caveat of the whole experimental approach here. I would have made more of a point of this except that the PYHIN factors pulled down different spectra of proteins. However, any other proteins that bind DNA are suspect as being false positive. Comment should be made on this. Also, figure 4D looks at co-precipitation of Ku80 and GFP-IFIX HIN or PYD. To make this data presentable, the sample needs to be well treated with DNase prior to or during the pull-down. Otherwise this needs to be left out. Proof of an association which depended on binding together to DNA would be problematic. How do you exclude independent binding of proteins to a long piece of DNA?

5. Figure 4B. Colocalization of IFIX and PML looks really good. DAXX and ATRX show no indication of colocalization. If you have expression of one component fairly diffusely all over the cell there will be apparent overlap of colors. Unless there is clear enrichment of both factors in a similar pattern, this does not indicate colocalization, as optical slices from confocal microscopy still have a finite thickness. The interpretation of this data is not appropriate.

6. Given that the N and C terminally tagged IFIX constructs really seem to have quite different localization, one diffuse in the nucleus, the other punctate, does it surprise you that the proteins you identified are similar? Were there any strong proteins that you have omitted that might relate specifically to the location in puncta?

7. If IFIX permits induction of IFN-b in response to DNA transfection, what is its role relative to cGAS which seems to be essential for the detection of foreign DNA leading to IFN. Do you propose they are both essential in this system? Is cGAS expressed in these cells?

Minor points

1. The authors draw incorrect analogies between mouse and human proteins (introduction page 3 - ifix mouse homolog pyhin1; results page 8 - IFI16 murine homolog p204). Published work shows clearly that AIM2 is the only gene with an ortholog in mouse and human (cited reference, Cridlan 2012). The other family members have expanded separately in each species. Thus IFI16 is not closely related to p204 and IFIX is not related to pyhin1 any more than they are to other mouse genes. In fact IFIX is quite unlike mouse pyhin1, having a different class of HIN domain. Nomenclature is problematic for these proteins. These proteins should not be discussed as if they are orthologs.

2. Results section says IFIX shows puncta (Figure 1C, arrows). There are no arrows on the IFIX panel.

3. Introduction. The reference to Pontillo, 2012 is out of place and mis-cited. This paper finds NLRP1 polymorphisms associated with lupus (not autoinflammation), and is unrelated to the PYHIN proteins being discussed here. This sentence should be deleted, I don't believe any autoinflammatory diseases have been connected with PYHIN proteins.

4. A NES is already defined for p204 which I believe is conserved in IFI16 (Mol Cell Biol 2000 20:7024). Is this a conserved sequence with what you identify?

5. It could be noted that GFP tends to dimerize, so this may have helped initiate some of the puncta.

6. IFIX puncta were observed with one IFIX isoform in an early published paper. This should be commented on.

7. Results section says "Likewise, no punctate structures were observed for MNDA". This is not likewise, as all other proteins showed some puncta.

Reviewer #2:

Summary
The authors have presented a comprehensive MS-based proteomic study that aims to develop a protein interaction network for all four members of the PYHIN family of proteins. PYHIN proteins are defined by the presence of PY domains (that mediate protein-protein interactions of inflammatory signalling complexes) and HIN200 domains (that bind to foreign/viral dsDNA). In this capacity PYHIN proteins act as immune sensors and inflammatory mediators when the cell is faced with infection by viruses. Despite ample evidence in the literature (from this lab and others) that the PYHIN family members IFI16 and AIM2 are functionally important as immune sensors, little is known regarding the function of the other two family members, IFIX and MNDA. The authors employ an inducible cell-based expression system, immuno-affinity purification of both N and C terminally GFP-tagged versions of the four PYHIN proteins, and label-free quantitative MS analysis to build an interactome for these important regulators of innate immunity. The authors subsequently demonstrate, using a combination of techniques (including fluorescence microscopy, biochemical, and cell-based assays), that IFIX is a bona fide immune sensor capable of binding to viral dsDNA and inducing cellular expression of an important inflammatory cytokine. Finally, the authors identify and test the function of a putative nuclear export signal (NES) within the PY domain of IFIX, which in combination with its predicted nuclear localization signal (NLS), is proposed to allow recognition of viral dsDNA in both the cytoplasm and the nucleus.

General remarks
The data are convincing enough to support many of the study conclusions (despite limitations with many of the microscopy experiments); however, in many places more extensive use of controls and some additional experimental parameters would be required for a truly high quality publication. The immunofluorescence data as presented are often problematic: In many instances it is not possible to distinguish the features described by the authors. This might be a conversion problem that occurred in the manuscript submission process, but images should be reprocessed to ensure that they are of the highest quality possible.

Probably the most interesting aspect of the work is the notion of there being a potential balance between nuclear import and export functions of IFIX, and how this may be different between the different PYHIN family members. However, this interesting finding did not logically stem from the proteomic aspect of the study, and could have been designed and implemented based purely on bioinformatic analysis of domains/sequences within the different family members. As such, novel mechanistic insight into the PYHIN family of proteins was not really gained from the interactome itself, and most of what was newly shown for IFIX could have been predicted based on previously known functions of the other family members. The paper therefore feels like two stories joined together - one which is a proteomic study of PYHIN family members that, although a useful resource on its own, itself didn't yield anything particularly novel about PYHIN protein function. This is then followed by a study demonstrating an innate immune sensor function for IFIX, which was mainly predicted from other family members, and did not require the initial proteomic study in order to be conceived.

As presented, the study therefore offers only an incremental advancement in our knowledge of viral innate immunity, and will be of interest primarily to specialists of the field rather than a broad audience such as that reached by Molecular Systems Biology.

In general the study would also greatly benefit from being more concisely presented. The entire paper is in need of extensive editing, as much of the introduction and results sections are laden with details that detract from the overall presentation of the key findings/conclusions. Furthermore, the flow of arguments is not always logically presented and ideas tend to "jump around" excessively.

Major points
1) In the introduction of the paper, the importance of studying the PYHIN family of proteins is made clear and the study rationale is evident; however, this information needs to be more concisely relayed. In many ways the introduction reads like a detailed review of the literature on PYHIN

© European Molecular Biology Organization

proteins. Far too much background detail is given in the results section as well, which should instead make use of only the most relevant references to the literature in order support the experimental rationale.

2) Are there antibodies available in order to perform Western blotting for endogenous PYHIN proteins? In Fig 1A it would be nice to see that this induction of PYHIN protein expression translates to the protein level. Furthermore, the ability to blot for endogenous proteins would be particularly useful to show what degree of overexpression is being achieved with the inducible expression system being employed. As it is, we have no way of knowing how close the induced expression is to endogenous levels. Furthermore, whether the endogenous proteins behave the same way as the transfected ones in immunofluorescence would be nice to comment on; this is particularly important for those proteins whose localization is affected when the different tags are used.

3) Too many conclusions are being drawn from insufficient microscopy evidence (note that the entire section on pages 9-11 is also very confusing). Examples include:
Fig 1C needs to be approached in a quantitative manner. At the very least, a large number of cells need to be imaged and the number of puncta need to be counted across the various conditions in order for any conclusions to be drawn regarding the presence or (especially) absence of puncta with the various constructs. The authors should also show a single representative cell with higher magnification for each constructs. In its current form, it is difficult to observe the substructures mentioned by the authors.
In Figure 4B, the IFIX staining shows a high degree of variability in between panels as well as signal saturation in some instances. To facilitate the comparison, the authors should ensure that the signal intensity is equal across samples (for instance the IFIX staining is much weaker in the PML column than in the DAXX/ATRX one). Furthermore, the amount of IFIX puncta staining should be quantified across many cells to determine the normal distribution of IFIX puncta.
Figure 5B would benefit from simple counting of the number cells that display IFIX puncta in VACV transfected vs non-transfected cells.
In Figure 5D again something quantitative is required. The ratio of the GFP signal to the Cy3 signal across a number of cells for the different mutants would be one approach. As it is, these two examples of a negative result are not fully sufficient to demonstrate that this is a functional NES.

4) The rationale for changing the SAINT score cut-off for different baits is not clear; how were these cut-offs selected? Are they based on overlap with the literature, precision recall, etc.?
Was the expression level in the negative control samples (and the localization of the expressed protein) comparable to that of the baits analysed? From the Figure 1E, it does not appear so (meaning that the GFP is detected at much lower levels than the other baits), and this can drastically affect the recovery of the contaminant (sticky) proteins. Since there is very little here in terms of validation of the new interactions, this is an important concern here. Furthermore, the total spectral counts across the three GFP replicates shown in Supplementary indicates a lot of variability in recovery, with sample 3 having twice as many spectra as sample 1. Since the software the authors chose to use (SAINT, and they are seemingly running each bait separately across the controls) depends critically of the negative controls, a major concern is that if these controls do not properly model the background, the conclusions are invalid. Have the authors tried to perform the same analysis but including many more controls and perhaps selecting the controls based on the total spectral counts in their negative control purifications? How stable is their interactome under these conditions?
It is also not clear the authors requirement of having a SAINT score {greater than or equal to} 0.85 (or 0.95) in the AP-MS results with both N- and C-terminal GFP tag to be considered "specific" since the immunofluorescence data presented in Figure 1C does suggest different behaviors when the tag location is changed. This should probably be commented on.
At the minimum, the authors should revisit their statistical analysis and generate suppl. table 3-5 anew and use this opportunity to improve their table naming scheme (the "specific common", "uncommon" and "non-specific uncommon" labels are really confusing and not widely use in the field).

5) Additional controls and/or experimental parameters would be helpful/essential in a few of the experiments. Examples include:
- Fig 1E - GFP blots of induced cell lysates to show how consistent the degree of

induction/expression is across the different baits would be beneficial.
- In Figure 1F, the blots presented should be continuous to enable relative comparison between fractions (which is not the case for MNDA and AIM2). In addition, the relative amount loaded in each lane should be shown in the panel or in the Figure legend.
- Fig 4C - removal and/or mutation of the NLS would be a nice control to show that it specifically directs nuclear localization. The use of an NLS mutated GFP-IFIX-HIN200 construct could then demonstrate whether Ku80 can only bind to IFIX when it is in the nucleus.
- In Figure 4D, there is an excess of Ku80 in the GFP-IFIX-HIN200 sample lane. As this sample is the one showing the positive results, the authors should rerun/repeat the experiments to obtain equal loading and strengthen their data. Furthermore, I would like to see an extra control sample where ethidium bromide is added to the samples to ensure that the interaction is not DNA dependent (see PMID: 1495986 for details). In this co-IP both bait and prey are known to associate with DNA so the DNaseI added in the buffer during lysis may not be enough to completely prevent DNA dependent protein interactions.
- Fig 5C - I would like to see that induction of an "irrelevant gene" such as GFP alone does not induce IFN expression in the presence of VACN dsDNA transfection. Furthermore, this experiment has good dynamic range, and therefore would be a particularly useful opportunity to test the various sequence/domain/structural features of IFIX with an actual functional readout. For example, testing the necessity of the NLS and the NES for IFN induction would be particularly informative.
- Fig 5C and E - a nice control would be to use AIM2 in these experiments, since it lacks an apparent NLS and localizes primary to the cytoplasm it might be predicted that it should therefore be unable to detect HSV-1 dsDNA but might still be able to detect the VACV transfected DNA.
- In Figure 5E, the WB needs to be displayed properly with both GFP and GFP-IFIX appearing on a single blot with molecular weight marker displayed properly. In its current format, the blot appears VERY suspicious.

6) In Figure 5B-D, the authors describe the role of IFIX and of its NES sequences in DNA sensing. To better characterize the function of IFIX here, the authors should test the capacity of their IFIX NES mutant in inducing IFN-beta expression in response to vaccinia virus DNA. One would expect that mutating the first NES motif would drastically reduce IFN-beta expression in response to VACV and thus, make a strong point for the authors' model.

7) The lengthy description of the expression results for the PYHIN proteins in HEK293 cells is unnecessary; simply state that they are expressed, and importantly, that their expression level is induced by interferons.

8) Have the authors attempted to perform some of the GFP-MS experiments after treatment with nucleases such as RNAse and DNAse to estimate how much of the interactome is due to protein-nucleic acid rather than protein-protein interactions? Have they tried to test the effect of IFN-b on the interactome?

Minor points
1) Fig 1A and S1A - it might be helpful to state that/whether these are all normalized to GAPDH expression, as this information is only available in the methods.

2) In the Supplementary Figures, the font choice and resolution seems particularly poor.

3) On page 9, the nature of the "protein-dependent cytotoxic effects" could be briefly mentioned.

4) I'm not sure the use of the word "pancellular" is appropriate - to me this implies "across all cell types". Something like "uniform distribution" might be more appropriate.

5) Fig 1D - In the workflow, why is "PTM mapping" included for this particular study? Unless I have missed something, this is not at all relevant.

6) For Figure 2B, I suggest that the authors expand their legend to describe the constituent of the main clusters (color bars) either by GO terms and/or protein complexes present in them.

7) In Figure 3, the authors should explore using the edge thickness to display spectral count information and/or the nodes outline for SAINT score (the better the thicker for instance).

8) The resolution of immunofluorescence images shown in Figure 4B and 4C is too low.

9) In Figure 5D, the authors seem to have forgotten to show the localization of the WT IFIX construct. While this was shown in Figure 1C, it would be easier for readers if it was also present in this panel.

Reviewer #3:

This review, as requested, comments only on the proteomics methodology and data analysis.

The authors have used a reasonably standard affinity-purification mass-spectrometry (AP-MS) strategy using GFP tags to explore the interactome of the PYHIN family of proteins.Both N- and C-terminal recombinant tagged pbait proteins have been used (and for which good agreement was seen) and in sum have identified ~350 PYHIN interacting proteins, as gauged using statistical techniques. The proteomics analysis is thorough and well-done; the authors have verified the expression patterns of the recombinant bait proteins as well as using a cell line that apparently expresses the endogenous PYHIN proteins. Data analysis is also quite adequate. A few clarifications would help the reader:

The authors mention comparison of identified proteins to control AP-MS experiments of their own (3 expts) as well as to the CRAPome resource for filtering AP-MS contaminants. They also use the Saint program to score each bait-prey pair. However it may help the reader to know how reproducible the prey hits were. This reviewer understands that at least 2 reps were performed for each bait - for how many preys were identifications made in both replicate AP-MS experiments? Also if a prey was identified in 1 or more control AP-MS - were these automatically excluded from further analysis?

New submission 13 October 2014

(point-by-point response: see next page)

Point-by-point reply to the reviewers' comments for the manuscript MSB-14-5332

We wish to express our gratitude to the reviewers for their insightful comments and recommendations. We are delighted that the reviewers appreciated the novelty of our findings with regard to understanding the interactions of the PYHIN proteins and the discovery of IFIX as a novel sensor of viral DNA. Based on the reviewers' advice, we have performed a series of additional experiments that address all of the reviewers' concerns and unequivocally support our conclusions. As a result, we have introduced one new figure (Figure 4), additional 11 new/revised figure panels (Figure 1B-C, Figure 5B-E, Figure 6B-E, G) within the manuscript, 11 new supplementary figures/panels, and have revised the text accordingly. As suggested by the reviewers, we have also tightened the introduction and the results sections. In what follows, we present a point-by-point answer to the reviewers' comments. The reviewers' comments are shown in italics and our answer is marked with ">". Page and figure numbers in our answers refer to the revised manuscript.

Reviewer #1:

This paper does a proteomic analysis of proteins interacting with human PYHIN (HIN-200) proteins in HEK293 cells. A large number of novel interactions were found which have a surprising degree of specificity for the different proteins, suggesting there are not many generic HIN or PYD interacting proteins which bind all family members. The authors' approach of tagging both N and C terminus seems to have yielded a reliable indication of interaction. The paper goes on to further characterize IFIX as a nuclear protein with a nuclear export sequence that binds transfected DNA in the cytosol, viral DNA in the nucleus, and associates with PML. IFIX is a protein little studied in the literature, and this work makes a useful contribution in this regard. It is not unexpected that IFIX will bind to foreign DNA and mediate responses to viruses, as this is found in other family members.
I think the main improvement which could readily be made to this paper is in the presentation of what is being studied and the system used. Interactions of these proteins are analyzed in HEK293 cells, without infection or introduction of DNA. The lack of DNA stimulus, as well as the lack of convincing role for these proteins in HEK293 cells, means that interactions with proteins related to foreign DNA responses may not be captured. However, there is a wealth of prior literature implicating PYHIN proteins in other processes such as senescence, apoptosis, cell cycle regulation, DNA damage recognition which may be part of normal homeostasis. The paper could better emphasize the use of these cells to define interactions of PYHIN proteins with widely expressed proteins, related to the broad range of suggested functions. The beginning of the results section tries to convince the reader that HEK293 cells are a good cell type in which to be analyzing interactions of PYHIN proteins related to their innate immune function. I am not convinced, for reasons given below, but I do I think that some useful results have been obtained.

> The reviewer makes several important points that we have carefully addressed. As detailed in the point-by-point reply to specific comments (please see below), we have performed a series of new experiments to: 1) demonstrate the validity of our interactions by comparing the interactions observed in HEK293 cells with interactions in cell types commonly used to study viral infection and induced immune responses—primary human fibroblasts and differentiated monocytes, and 2) assess interactions following stimulation with foreign DNA by performing isolations following infection with herpes simplex virus 1 (HSV-1). Additionally, as indicated by the reviewer, our initial manuscript failed to emphasize enough that the PYHIN proteins, in addition to their roles in immune response, have important functions in transcription and chromatin assembly, apoptosis, cell cycle regulation and DNA damage response. To address the reviewer's concern, we have now rewritten the introduction and have added to our interpretation of the results to emphasize these important additional PYHIN protein functions.

> As detailed below, we have addressed all the specific comments and we thank the reviewer for the careful assessment of our paper and all the constructive suggestions; we think that the manuscript is substantially improved as a result of this revision. We are excited to report the first study of the protein interaction networks for this family of proteins, and to define IFIX as a novel sensor, able to detect viral DNA and trigger interferon response. As the reviewer indicates, despite a great interest in this family of proteins, there is still very little knowledge regarding their interactions. Not only is there little information about the interactions of IFI16 and AIM2 with regards to their house-keeping functions or immune response, but there is even less known about MNDA or IFIX, and the functions of IFIX remain to be characterized. In fact, currently there are only two papers that studied IFIX. Therefore, its role in sensing and immune response remained to be elucidated and is addressed for the first time in our manuscript. We respectfully disagree with the reviewer's comment that this is "not unexpected"… "as this is found in other family members". We agree that, given the presence of a HIN domain, it was likely for IFIX to bind DNA, similar to IFI16 and AIM2 from the same family of proteins. However, the function mediated by this DNA binding was not clear or expected. AIM2 binds viral DNA, but does not trigger interferon response and functions within a distinct pathway from IFI16. Furthermore, MNDA is not known to bind to viral DNA or act as a sensor. Therefore, the ability of IFIX to bind viral DNA and the function and nature of this binding have not been characterized prior to our study.

Specific comments
1. A test of the assertion that HEK293 cells are a good model to assess interactions of PYHIN factors is the fact that the approach used cannot expose the known role of AIM2, in initiating an inflammasome response. HEK293 are used a lot for reconstituting innate immune pathways, and they are useful because they lack most of these receptor systems. The results section says "We and others have previously demonstrated IFI16- and AIM2-dependent immune functions" in HEK293. I have not checked all these papers but the cited Fernandes-Alnemri paper shows AIM2 dependent functions in these cells only by transfecting in all the components of the system which are lacking:- AIM2, ASC, caspase-1 and IL-1b. This is hardly a recommendation for this cell line as a model of AIM2 responses. The whole opening of the paper seeks to validate HEK293 as a cell line where PYHIN proteins are normally active. This is not convincing, and an alternative approach is needed. I do not believe that this cell line has significant levels of expression of the factors claimed. MNDA for instance is exquisitely myeloid restricted, as shown in online array data (GNF symatlas). A reference which looks like it is included to support HEK expression of MNDA does not use HEK cells (Fotouhi-Ardakani). Expression data shown in the paper is real time PCR, normalized so that no impression can be gained of the absolute level of expression. I recommend abandoning the attempt to convince readers that HEK293 cells normally have functional levels of all these PYHIN proteins. But if this expression data is to be shown, it should be shown as just the ratio of gene expression to a standard housekeeping control gene, so that an impression can be gained of absolute level. Samples coming up at late cycle number are of little consequence. It would also have been better to compare expression to peripheral blood cells, which really do express these factors. Even then, if you wanted to convince us that these factors are expressed, there are very good antibodies for AIM2 and IFI16.

> We absolutely agree with the reviewer that these cells are not ideal for studying immune response; however, there are currently no antibodies available for IFIX and MNDA; we have tried to generate one for IFIX, spending quite a bit of time and funds for this, and it is unfortunately not appropriate for immunoaffinity purification (IP) studies. While not ideal, these HEK293 cells have provided a common system for all four PYHIN proteins, allowing their tagging and induction using tetracycline. To address the reviewers concerns we have included additional experiments (point 1.2 below) and have also included additional clarification (points 1.1 and 1.3 below):

> 1.1. Clarification: While, as stated above, we completely agree that these cells are not ideal for studying immune response, we would like to clarify that the HEK293 cells that we used are capable of inducing IFN-β in response to foreign DNA. We have already demonstrated this in the manuscript by showing in previous figure 5C (current figure 6E) that IFIX is necessary in these cells for the induction of IFN-beta following binding to foreign DNA (from vaccinia virus, VACV). These inducible cells express the protein STING (a known hub for DNA stimulated immune signaling) and IRF3 (a transcription factor required for IFN induction) and we have now added a western blot showing this (new supplementary figure S1C); this is why we have also shared these cells with Andrew Bowie's lab in Scotland to be used in their immune response studies. The reviewer is correct that the inflammasome function of AIM2 can only be recapitulated in these cells by co-transfection of inflammasome components (ACS, caspase-1), and that these cells are not otherwise a good model for showing inflammasome activity. We have stated the lack of these components and of inflammasomes in our originally submitted manuscript; we have now trimmed the introduction and results sections to avoid any misunderstanding. In this paper, we focused on the ability of IFIX to bind to foreign DNA and help induce interferon expression, and these cells are usable for these studies, being known to be able to induce interferon response. In this paper, we are not studying inflammasome functions of the PYHIN family of proteins. The IFN-β response was also previously reported in these cells and used to characterize other DNA sensor functions, such as for cGAS (e.g., Ablasser A., Nature 2013; Ablasser A, Journal of immunology 2014), and we have now included reference to these papers in our manuscript, and have clarified this aspect (page 7).

> 1.2. New Experiments: To further validate the value of our interactome dataset in HEK293 cells, we have added new isolations in cells commonly used to study viral infection and resulting immune response—differentiated monocytes (THP1 cells) and primary fibroblasts (HFF cells) (new Figure 4). We focused our analyses on the interactions of endogenous IFI16, for which we have tested antibodies for efficient immunoaffinity purifications of the endogenous protein. THP1 cells are routinely used in innate immune studies, and we (Li et al, Cell Host Microbe 2013) and others (Orzalli et al, PNAS 2012) have already reported the immune function of IFI16 in HFFs following infection with herpesviruses. We assessed the expression and localization of IFI16 in these cells. While IFI16 is predominantly nuclear in HEK293 and HFF cells, it is localized to both the nucleus and the cytoplasm in THP1 cells (Figure 4). Therefore, while our HEK293 and HFF experiments would mainly reflect nuclear interactions, the THP1 cells would reflect both nuclear and cytoplasmic. To ensure an adequate comparison of the IFI16 nuclear interactions, we fractionated the differentiated THP1 cells and performed isolations from the nuclear fraction. While also detecting cell-specific associations, overall, our results demonstrate a significant overlap in the IFI16 interactions in HEK293, differentiated monocytes and primary fibroblasts. Approximately 75% of the interactions detected for the GFP-tagged IFI16 in HEK293 cells were also observed for the endogenous IFI16 in differentiated monocytes and fibroblasts. Therefore, in addition to validating and complementing our current interactome in HEK293 cells, these studies of IFI16 interactions will be important for the scientific community in further studying the diverse functions of IFI16 in immune response and transcription regulation. These results emphasize that there are numerous important interactions of PYHIN proteins that are revealed by our interactome study in HEK293 cells.

> 1.3. Clarification: As recommended by the reviewer, our manuscript was reworded to better emphasize the bigger picture of our interactome datasets. The reviewer is absolutely correct with this remarks, and we expect this interactome to provide a much-needed resource for future studies on these proteins, which are not limited to their functions in immune response, but also in transcription, cell cycle, apoptosis etc.

2. Similarly for Figure 5C and Sup Fig 1A, please don't normalize PCR results to one of the samples, but show data normalized to gapdh control for each sample. Unnecessary normalization loses information.

> In our initial submission, in Sup Fig. 1A, we normalized the mRNA expression of each *pyhin* gene by cellular *gapdh* levels in each cell type. We then selected HeLa cells as our point of reference for relative quantification, as the expression of all four *pyhin* genes appeared to be the lowest in HeLa cells (relative to the other cell types assayed). Thus, this was merely done for data presentation purposes. Given the reviewer's concerns, we have chosen to remove this panel to avoid any misunderstandings. We wanted to avoid any misinterpretation about the extent to which *pyhin* genes are expressed based on the usage of HeLa cells as the point of reference. Furthermore, as our initial figure showed comparison of HEK293 cells and HFFs with two cell types (CEM-T lymphocytes and HeLa cells) that were not further used in our study, we felt that this panel was of limited value. Finally, we felt that it was more important to demonstrate that all 4 *pyhin* genes were inducible by interferon within HEK293 cells. Additionally, we felt that it was more relevant to include Western blots (Supplementary Figure S1B) showing the expression of basic components of cell intrinsic immune signaling (STING and IRF3) in HEK293.
> As for the other qPCR experiments shown in the paper (current Supplementary Figure S1A, Figure 5E, and Figure 6E (formerly Figure 5C)), we respectfully disagree with the reviewer that the "delta delta Ct" is inappropriate for displaying relative qPCR-based quantification data. We have analyzed our qPCR data according to the methods developed by Livak and Schmittgen (2001) (PMID: 11846609). The wide acceptance of this method is underscored by the fact that it was referenced by more than 40,000 scientific literatures (google scholar) and specifically used to quantify relative IFN-beta induction (e. g. PMID: 19631370, 23258413, 20890285). We hope that the reviewer will agree that this is a suitable representation of relative fold changes in interferon expression and IFIX levels.

3. Although some of the protein:protein interactions described here may be relevant in the response to foreign DNA, it should be noted that the pull downs were done from cells not exposed to such DNA. Thus these interactions might be more relevant to other homeostatic roles of these proteins. This may provide a better focus for the paper.

> We completely agree with the reviewer's comment that many of these interactions will be reflective of the house-keeping functions of PYHIN proteins, and we believe that there is great value in this. We have revised the introduction and results sections to better emphasize the diverse roles of the PYHIN proteins. Furthermore, we have also strengthened the value of identifying the IFIX-PML interaction, demonstrating that both of these cellular proteins act as antiviral factors, impacting the growth of HSV-1 (new Figure 5C-E). Additionally, to further address the reviewer's comment, we performed new experiments to characterize the interactions of the novel sensor that we report in this manuscript, IFIX, in response to foreign DNA. Given our findings that IFIX binds to herpesvirus DNA, we have selected the HSV-1 infection as a relevant form of stimulation with foreign DNA. Interestingly our results showed that numerous IFIX interactions are conserved and enriched following infection with HSV-1 (new supplementary Figure S12). Whether these diverse interactions play roles in housekeeping functions of IFIX or in its antiviral roles remains to be elucidated. It is conceivable that the tetracycline-induction of IFIX partly mimics a cellular state in which IFIX is induced by interferon, which would potentially explains why many interactions are shared between uninfected and infected cells states. Additionally, we observe that IFIX interacts with several viral proteins, including UL39 that possessed anti-apoptotic activities and is required for efficient HSV-1 replication. This interaction may act to inhibit IFIX functions or may represent means for IFIX to limit viral replication. We are discussing these aspects on page 24.

4. I think conclusions about PYHINs associating with DNA damage factors like Ku proteins are problematic. If you pull down HEK cell extracts with DNA, the dominant proteins found are Ku. Pull-downs of PYHINs will have associated DNA, and this will bring down DNA damage proteins without them necessarily being in any direct association with the PYHIN. This is in fact a caveat of the whole

experimental approach here. I would have made more of a point of this except that the PYHIN factors pulled down different spectra of proteins. However, any other proteins that bind DNA are suspect as being false positive. Comment should be made on this. Also, figure 4D looks at co-precipitation of Ku80 and GFP-IFIX HIN or PYD. To make this data presentable, the sample needs to be well treated with DNase prior to or during the pull-down. Otherwise this needs to be left out. Proof of an association which depended on binding together to DNA would be problematic. How do you exclude independent binding of proteins to a long piece of DNA?

> The reviewer is correct that associations with DNA-binding proteins are challenging to interpret. We consider this very important and have experience with such studies, as many projects in our lab focus on DNA-binding proteins. This is why all of our immunoaffinity purification analyses have already been done following treatment with DNase. This should have been part of our methods section, but, unfortunately, as the reviewer noted, it may not have been highlighted enough. We have now revised the text to clearly emphasize that all of our experiments, including the IP-MS and the validation of the interaction with Ku proteins, have all been done in the presence of DNase (page 9). Furthermore, the new isolations that we have included for IFIX following HSV-1 infection and for IFI16 in fibroblasts and THP1 cells were all done in the presence of benzonase, which digests both types of nucleic acids (RNA and DNA) and is more effective in a wider range of buffer conditions than DNase. The association of IFI16 with the Ku80 was observed in HEK293 cells, as well as with endogenous IFI16 in differentiated monocytes in the presence of benzonase (new Figure 4).
> One change to mention is that, given the addition of numerous new figures and tables, we have removed our results describing the interaction of the HIN domain with Ku80. We aimed for the revised manuscript to be more focused and present a proper flow from the interaction to our functional analyses on IFIX. Therefore, we have added several new experiments (Figure 5C-E and Figure 6) which we feel more directly characterize IFIX function as a broad-acting intracellular DNA sensor and general antiviral factor. We have performed new experiments demonstrating that, similar to PML, the levels of IFIX impact viral replication (new Figure 5C-E). Furthermore, we expanded our characterization of the binding of IFIX to dsDNA. We now show that IFIX binds both circular and linear DNA, therefore not having preference for DNA ends (new Figure 6B). We also generated an array to assess binding to dsDNA and demonstrated that IFIX binds DNA in a sequence nonspecific manner (Figure 6C). These properties strengthen our conclusion that IFIX acts as an effective sensor of foreign dsDNA. Overall, this revision allowed us to generate a better flow for the manuscript and the main points that we want to get across to the readers. We feel that as a result, the manuscript is much more focused.

5. Figure 4B. Colocalization of IFIX and PML looks really good. DAXX and ATRX show no indication of colocalization. If you have expression of one component fairly diffusely all over the cell there will be apparent overlap of colors. Unless there is clear enrichment of both factors in a similar pattern, this does not indicate colocalization, as optical slices from confocal microscopy still have a finite thickness. The interpretation of this data is not appropriate.

> To address the reviewer’s concern, we have performed additional microscopy studies and have added new images for ATRX and DAXX (Figure 5B). As described in our results section, these images illustrate partial co-localization between IFIX. This partial co-localization is expected as ATRX and DAXX are known to be transiently associated with PML bodies, and therefore, would have additional localizations. We are mentioning this on page 18.

6. Given that the N and C terminally tagged IFIX constructs really seem to have quite different localization, one diffuse in the nucleus, the other punctate, does it surprise you that the proteins you

identified are similar? Were there any strong proteins that you have omitted that might relate specifically to the location in puncta?

> As indicated in our manuscript, for all PYHIN proteins we chose to focus on the common interactions for their N and C terminally tagged proteins in order to limit interactions that may be altered or formed by the tagging. With regard to the interactions of IFIX, the reviewer is correct in stating that, while IFIX had either nuclear diffuse or nuclear punctate appearance when tagged at the N- or C-terminus, most interactions were common. We have now included quantification across hundreds of cells to better characterize the localization of the four PYHIN proteins (see new Figures B-C). As shown in new Figure 1B and new supplementary Figure S2C, while the IFIX localization within nuclear puncta exists in most cells (quantified in Figure 1B), this punctate localization frequently coexists with diffuse nucleoplasm staining. Therefore, many interactions are expected to be common. One other factor that is worth noting is that, for all C-terminally tagged proteins, we observe some degree of loss of the bait protein in the pelleted fraction prior to the IP (Figure 1F). This suggests that the lysis buffer conditions used may not be stringent enough to fully capture the proteins present in the aggregates/puncta. We tried to maintain a balance in the stringency of the lysis buffer, in which we obtained a good recovery of the tagged proteins, but still retained interactions. We have revised the corresponding results sections to discuss these aspects.

7. If IFIX permits induction of IFN-b in response to DNA transfection, what is its role relative to cGAS which seems to be essential for the detection of foreign DNA leading to IFN. Do you propose they are both essential in this system? Is cGAS expressed in these cells?

> The reviewer brings up an exciting research topic, which is of great interest to us. Several labs, including ours (Cell Host Microbe 2013, PNAS 2012) have shown that IFI16 is required for the detection of herpesvirus DNA within the nucleus leading to IFN response during infection. More recently, cGAS was also found as a sensor of foreign DNA, and currently shown to function in the cytoplasm. Here, we show that IFIX is also contributing to the sensing of foreign DNA to induce interferon expression. The connections between IFI16 and cGAS or between IFIX and cGAS are currently unknown, and defining the mechanisms or possible functional connections is outside the scope of our manuscript. We did not observe cGAS among IFIX or IFI16 interactions, and we did not detect it in whole HEK293 cell lysates by western blotting. Therefore, we do not see yet a connection to pursue this, and this would form a different project. However, it is tempting to hypothesize several possible models. As IFIX is required for induction of IFN-beta expression in these cells (as shown in this study in Figure 6) and IFI16 was reported to be required for IFN-beta response in 293 cells (PNAS 2012), these results would suggest that IFIX and IFI16 do not require the presence of cGAS for their functions in DNA sensing. Nevertheless, in other cell types, cGAS could act upstream of IFIX or there could be separate, parallel DNA sensing mechanisms that may act to amplify the propagation of immune signals.

Minor points

1. The authors draw incorrect analogies between mouse and human proteins (introduction page 3 - ifix mouse homolog pyhin1; results page 8 - IFI16 murine homolog p204). Published work shows clearly that AIM2 is the only gene with an ortholog in mouse and human (cited reference, Cridlan 2012). The other family members have expanded separately in each species. Thus IFI16 is not closely related to p204 and IFIX is not related to pyhin1 any more than they are to other mouse genes. In fact IFIX is quite unlike mouse pyhin1, having a different class of HIN domain. Nomenclature is problematic for these proteins. These proteins should not be discussed as if they are orthologs.

> We thank the reviewer for the constructive criticism. As it is unclear whether IFIX and murine Pyhin1 are true orthologs or not, we have removed this comparison from the introduction. For IFI16 and p204, however, we cautiously argue that a possible functional homology could exist, as both proteins are the only ones that contain two HIN domains and a pyrin domain of their respective species (Cridlan 2012), and both proteins were shown to function as innate DNA sensors (Unterholzner 2010). In addition, results in this manuscript (Figures 3 and 4) and others (Lee 2013) showed that both IFI16 and p204 interact with the ATP-binding cassette family protein ABCF1. Therefore, although the two may not be strictly defined as sequence orthologues, functional analogies are supported by existing and emerging evidence and may benefit future investigations into these dual-HIN-domain proteins. To avoid any potential confusion, we have revised the sentence to:
"ABCF1 was recently reported to interact with a murine PYHIN protein p204 (Lee et al, 2013), which like IFI16 regulates cytokine induction in response to dsDNA (Unterholzner et al, 2010)."

2. Results section says IFIX shows puncta (Figure 1C, arrows). There are no arrows on the IFIX panel.

> Thank you. We have revised this figure to include statistical assessment of protein localization in numerous cells, and have corrected the absence of arrows.

3. Introduction. The reference to Pontillo, 2012 is out of place and mis-cited. This paper finds NLRP1 polymorphisms associated with lupus (not autoinflammation), and is unrelated to the PYHIN proteins being discussed here. This sentence should be deleted, I don't believe any autoinflammatory diseases have been connected with PYHIN proteins.

> We have removed the sentence and have revised the introduction to make it more focused.

4. A NES is already defined for p204 which I believe is conserved in IFI16 (Mol Cell Biol 2000 20:7024). Is this a conserved sequence with what you identify?

> The NES previously defined for p204 *(Mol Cell Biol 2000 20:7024)* spans from Residue 24 to 33, which correspond to the second NES motif identified in this work. A sequence alignment is provided below to illustrate the NES sequences identified by us for IFI16 and others for p204. Our results showed that only NES1 of IFI16, which is distinct from the putative NES of p204, is the only one required for cytoplasmic IFI16 to detect transfected DNA. We also would like to point out that these sequences at the N-terminus of IFI16 and p204 fall within their corresponding pyrin domains. Significant mutations to these sites may also negatively impact the proper folding of the pyrin structure and the functions of other motifs within this domain. Therefore, we chose to engineer the smallest mutations possible to these sites, which are alanine substitutions for leucine or isoleucine residues. In contrast, the work on p204 NES only considered a mutant that completely removed residues from 24 to 33, and consequently may render the entire pyrin domain dysfunctional.

```
                      NES-1              NES-2
IFI16  1    MGKKYKNIVLLKGLEVINDYHFRMVKSLLSNDLKLNLKMREEYDKIQIADLMEEKFRGDA  60
            M   +YK IVLL+GLE IN ++F + KSLL+ DL L     +E+Y  IQIA++MEEKF  D+
P204   1    MVNEYKRIVLLRGLECINKHYFSLFKSLLARDLNLERDNQEQYTTIQIANMMEEKFPADS  60
                                24 ---------- 33 NES reported for p204
```

> We have included the above answer just to make sure that we address all the questions raised by the reviewer. As we have now included substantial new experiments to 1) strengthen our interactome data (new Figure 4 and supplementary Figures S5-S9)), 2) provide a study of IFIX interactions during HSV-1

infection (new Figure S12), and 3) further characterize the role of IFIX as a sensor and its impact on HSV-1 infection (new panels in Figures 5 and 6), our manuscript became quite lengthy. To make sure that we maintain a good flow for the reader, we have now removed our experiments that defined the IFIX NES to make space for these new studies. This ensured a better transition from the interaction studies to our experiments defining IFIX as a DNA sensor, and a more focused manuscript.

5. It could be noted that GFP tends to dimerize, so this may have helped initiate some of the puncta.

> This is an important point and we are aware of this. It is noteworthy that we do not observe puncta in the free GFP cell lines that we have generated, in the N-terminus tagged proteins, or in the C-terminus tagged MNDA. Therefore, the puncta is likely not to be driven by GFP dimerization alone, but by some spatial accumulation of the protein. Furthermore, we have used GFP tagging in many of our previous interaction studies, including our interactome of the eleven human histone deacetylase and of numerous viral proteins and have not observed this type of puncta. However, this does not completely eliminate the possibility that the GFP may still further enhance the puncta appearance once a protein is accumulated within a spatially constrained region.

6. IFIX puncta were observed with one IFIX isoform in an early published paper. This should be commented on.

> Thank you for pointing this out. Nuclear puncta has been observed for the gamma isoform of IFIX. We have now added this information and the relevant reference to our manuscript (page 8).

7. Results section says "Likewise, no punctate structures were observed for MNDA". This is not likewise, as all other proteins showed some puncta.

> Thank you for noticing this error, which we have now corrected.

Reviewer #2:

Summary

The authors have presented a comprehensive MS-based proteomic study that aims to develop a protein interaction network for all four members of the PYHIN family of proteins. PYHIN proteins are defined by the presence of PY domains (that mediate protein-protein interactions of inflammatory signalling complexes) and HIN200 domains (that bind to foreign/viral dsDNA). In this capacity PYHIN proteins act as immune sensors and inflammatory mediators when the cell is faced with infection by viruses. Despite ample evidence in the literature (from this lab and others) that the PYHIN family members IFI16 and AIM2 are functionally important as immune sensors, little is known regarding the function of the other two family members, IFIX and MNDA. The authors employ an inducible cell-based expression system, immuno-affinity purification of both N and C terminally GFP-tagged versions of the four PYHIN proteins, and label-free quantitative MS analysis to build an interactome for these important regulators of innate immunity. The authors subsequently demonstrate, using a combination of techniques (including fluorescence microscopy, biochemical, and cell-based assays), that IFIX is a bona fide immune sensor capable of binding to viral dsDNA and inducing cellular expression of an important inflammatory cytokine. Finally, the authors identify and test the function of a putative nuclear export signal (NES) within the PY domain of IFIX, which in combination with its predicted nuclear localization signal (NLS), is proposed to allow recognition of viral dsDNA in both the cytoplasm and the nucleus.

General remarks

The data are convincing enough to support many of the study conclusions (despite limitations with many of the microscopy experiments); however, in many places more extensive use of controls and some additional experimental parameters would be required for a truly high quality publication. The immunofluorescence data as presented are often problematic: In many instances it is not possible to distinguish the features described by the authors. This might be a conversion problem that occurred in the manuscript submission process, but images should be reprocessed to ensure that they are of the highest quality possible.

Probably the most interesting aspect of the work is the notion of there being a potential balance between nuclear import and export functions of IFIX, and how this may be different between the different PYHIN family members. However, this interesting finding did not logically stem from the proteomic aspect of the study, and could have been designed and implemented based purely on bioinformatic analysis of domains/sequences within the different family members. As such, novel mechanistic insight into the PYHIN family of proteins was not really gained from the interactome itself, and most of what was newly shown for IFIX could have been predicted based on previously known functions of the other family members. The paper therefore feels like two stories joined together - one which is a proteomic study of PYHIN family members that, although a useful resource on its own, itself didn't yield anything particularly novel about PYHIN protein function. This is then followed by a study demonstrating an innate immune sensor function for IFIX, which was mainly predicted from other family members, and did not require the initial proteomic study in order to be conceived.

As presented, the study therefore offers only an incremental advancement in our knowledge of viral innate immunity, and will be of interest primarily to specialists of the field rather than a broad audience such as that reached by Molecular Systems Biology.

In general the study would also greatly benefit from being more concisely presented. The entire paper is in need of extensive editing, as much of the introduction and results sections are laden with details that detract from the overall presentation of the key findings/conclusions. Furthermore, the flow of arguments is not always logically presented and ideas tend to "jump around" excessively.

> We thank the reviewer for the constructive comments. As detailed below in the point-by-point reply to the specific comments (please see below), we have now added numerous control experiments, quantitative measurements for the microscopy images (i.e., cell count), and present a series of additional experimental evidence that address all the concerns and questions raised. Among these, we have added experiments on PML and IFIX to generate a better transition between the interactome study and the functional analyses on IFIX. Furthermore, we have followed the reviewer's suggestion and have revised the introduction and the results sections to make them shorter and more focused. We believe that our study will be of interest to a broad scientific community, as we report the first study of the protein interaction networks for PYHIN proteins and define IFIX as a novel sensor. Our interactome study provides a rich resource for numerous future studies on characterizing the mechanisms involved in the important housekeeping functions (transcriptional regulation, apoptosis, cell cycle) of these PYHIN proteins, as well as in their roles in antiviral and immune response. Furthermore, our functional studies on IFIX establish this protein as only the second sensor identified to date able to sense viral DNA both in the nucleus and cytoplasm. Therefore, our study will be of value to scientists from various fields of research, including transcriptional regulation, apoptosis, immune response, and antiviral functions. We thank you for all your comments, which we have carefully addressed; please see our specific answers below.

Major points

1) In the introduction of the paper, the importance of studying the PYHIN family of proteins is made clear and the study rationale is evident; however, this information needs to be more concisely relayed. In many ways the introduction reads like a detailed review of the literature on PYHIN proteins. Far too much background detail is given in the results section as well, which should instead make use of only the most relevant references to the literature in order support the experimental rationale.

> We agree with this concern. Initially, we wanted to give the broad audience of MSB a general idea of the many cellular functions in which the PYHIN proteins are involved. As this is the first study of the protein interactions for these proteins, our excitement to properly present the current knowledge and the significance of these proteins resulted in a thorough, but unfortunately lengthy, Introduction and Results sections. We agree that the overall wordiness has detracted from the focus of the manuscript. Consequently, we have now shortened the introduction considerably, and have also removed some of the interpretations and reiterations from the Results sections.

2) Are there antibodies available in order to perform Western blotting for endogenous PYHIN proteins? In Fig 1A it would be nice to see that this induction of PYHIN protein expression translates to the protein level. Furthermore, the ability to blot for endogenous proteins would be particularly useful to show what degree of overexpression is being achieved with the inducible expression system being employed. As it is, we have no way of knowing how close the induced expression is to endogenous levels. Furthermore, whether the endogenous proteins behave the same way as the transfected ones in immunofluorescence would be nice to comment on; this is particularly important for those proteins whose localization is affected when the different tags are used.

> We agree with Reviewer 2 that the use of endogenous antibodies targeting the PYHIN proteins would be ideal. Unfortunately, antibodies are not available for IFIX and MNDA. In fact, given our findings regarding IFIX functions in host defense against viruses, we have tried to generate an antibody against this protein. However, after quite a bit of time and money spent in this, we still did not obtain an antibody of sufficient quality for either western blotting or immunoaffinity purification. Additionally, in our hands, the multiple anti-AIM2 antibodies that we have tested were unsuitable for Western blot, immunofluorescence, and immunoaffinity purification experiments. The lack of antibodies is a main reason for designing and generating this cell-based system, which allowed us to have a uniform platform for studying all PYHIN proteins. We have now clarified this in the results section on page 7. To further address the reviewers concern, we performed several new experiments and have made changes to the manuscript, as detailed below:

a) For IFI16, which is the only PYHIN protein for which we have suitable antibodies, we have performed immunoaffinity purifications of endogenous IFI16 in two cell types relevant in studying immune response to viral infection (primary fibroblasts and differentiated monocytes). We have included a new results section (pages 15-17) and a new figure (Figure 4) that showed that ~75% of the interactions observed for IFI16-EGFP in HEK293 cells were retained with endogenous IFI16 in these different cell types.

b) We also clarified the writing in the results section to indicate that the localization observed for the GFP-tagged PYHIN proteins is in agreement with previous literature reports. In fact, even nuclear puncta was previously reported for IFIX, but for its gamma isoform. We have revised the text and have added the necessary references (page 8).

c) As an additional note, one intriguing aspect in studying these proteins that has to be considered is that inducing these proteins may actually be beneficial for identifying their functional interactions. As

the PYHIN proteins are interferon-inducible, their functions are induced in conjunction with their elevated levels. We have now introduced a brief discussion of this point on page 23.

3) Too many conclusions are being drawn from insufficient microscopy evidence (note that the entire section on pages 9-11 is also very confusing). Examples include:
Fig 1C needs to be approached in a quantitative manner. At the very least, a large number of cells need to be imaged and the number of puncta need to be counted across the various conditions in order for any conclusions to be drawn regarding the presence or (especially) absence of puncta with the various constructs. The authors should also show a single representative cell with higher magnification for each constructs. In its current form, it is difficult to observe the substructures mentioned by the authors.

> In response to Reviewer's 2 suggestions, we have performed additional microscopy analyses and the Figure 1 has been extensively revised. First, the images comprising Figure 1B have all been changed as the reviewer suggested. We now include a single representative cell at high magnification. Second, we have performed a new large-scale microscopy experiment in which we have unbiasedly counted hundreds of cells across many fields of view for each N- and C-terminally tagged construct of IFI16, IFIX, MNDA and AIM2. The results support our initial observations and are shown in a new panel (Figure 1C). Together, Figures 1B and 1C demonstrate that the observed punctate structures change dramatically in frequency between the N- and C-terminally tagged IFIX constructs. While statistically significant, the effect is lesser for AIM2 and IFI16, and no puncta are observed in either MNDA construct. To supplement Figure 1B and 1C, we have added supplementary Figure S2 which includes examples of the observed types of localization discussed in the manuscript. In addition to adding these statistical measurements, we have now reduced the interpretation of the observed localizations to avoid overstatements (pages 8-9).

In Figure 4B, the IFIX staining shows a high degree of variability in between panels as well as signal saturation in some instances. To facilitate the comparison, the authors should ensure that the signal intensity is equal across samples (for instance the IFIX staining is much weaker in the PML column than in the DAXX/ATRX one). Furthermore, the amount of IFIX puncta staining should be quantified across many cells to determine the normal distribution of IFIX puncta.

> Given the addition of new experiments and figures, this comment now refers to Figure 5B. As shown in new Figure 1B and new supplementary Figure S2, while the IFIX localization within nuclear puncta exists in most cells, this punctate localization frequently coexists with diffuse nucleoplasm staining. To address the reviewer's concern, we have performed additional microscopy studies and have added new images for ATRX and DAXX (Figure 5B). As described in our results section, these images illustrate partial co-localization between IFIX and these transiently associated PML body components. Furthermore, as stated above, in response to the reviewer's comment we have included quantification across many cells to demonstrate the prominence of IFIX within nuclear puncta (new Figure 1C).

Figure 5B would benefit from simple counting of the number cells that display IFIX puncta in VACV transfected vs non-transfected cells.

> We thank Reviewer 2 for the suggestion and agree that the quality of the manuscript benefits from such quantitative studies. For this reason and at the recommendation of Reviewer 2, we performed the

quantitative study shown in current Figure 1C (see above). However, in the panel mentioned here (current Figure 6D), we are illustrating for readers that IFIX co-localizes with transfected VAVC 70mer in the cytoplasm. We feel that because we do not observe any cytoplasmic IFIX puncta in non-transfected cells (having scored over 400 cells; see new Figures 1B-C and Figure SX), that quantifying the increase in cytoplasmic puncta from 0% in non-transfected cells may be misleading to readers. To further clarify the difference in localization, we have now included in the same panel an image of untransfected cells (Figure 6D, no transfect.). In addition to images shown in current Figure 6D, we feel we have unbiasedly and sufficiently demonstrated the non-random distribution of IFIX to VACV 70mer-containing puncta.

In Figure 5D again something quantitative is required. The ratio of the GFP signal to the Cy3 signal across a number of cells for the different mutants would be one approach. As it is, these two examples of a negative result are not fully sufficient to demonstrate that this is a functional NES.

> In this revised manuscript, we have added a substantial amount of new experiments to both strengthen our interactome and our discovery of IFIX as a novel DNA sensor. Given the addition of numerous new figures and tables, we have removed our results describing the identification of the NES for IFIX. In its place, we have added several new experiments (Figure 5C-E and Figure 6), which we feel more directly characterize IFIX function as a broad-acting intracellular DNA sensor and general antiviral factor. We have performed new experiments demonstrating that, similar to PML, the levels of IFIX impact viral replication (new Figure 5C-E). Furthermore, we expanded our characterization of the binding of IFIX to dsDNA. We now show that IFIX binds both circular and linear DNA, therefore not having preference for DNA ends (new Figure 6B). We also generated an array to assess binding to dsDNA and demonstrated that IFIX binds DNA in a sequence nonspecific manner (Figure 6C). These properties strengthen our conclusion that IFIX acts as an effective sensor of foreign dsDNA. Overall, this revision allowed us to generate a better flow for the manuscript and the main points that we want to get across to the readers. We feel that as a result, the manuscript is much more focused.

4) The rationale for changing the SAINT score cut-off for different baits is not clear; how were these cut-offs selected? Are they based on overlap with the literature, precision recall, etc.?

> In our previous publication of the interactome of human histone deacetylases (Joshi et al, Molecular Systems Biology 2013), we constructed several Receiver Operating Characteristic (ROC) curves to estimate true and false positive rates and guide our selection of SAINT score cut-offs. However, for the HIN200 interactome, the lack of significant numbers of well-established interactions precluded this type of analysis. Therefore, to select SAINT score cut-offs we constructed SAINT score distribution histograms, under the assumption that, in the ideal case, the distribution would reflect two populations, i.e. specific and non-specific interactions (see Supplementary Fig S4). While all scoring distributions were similar, for example SAINT scores < 0.50 clearly reflected a separate population of non-specific interactions, slight variations in the scoring distributions > 0.50 between HIN200 proteins required us to select different scoring thresholds to balance specificity (false positive identifications) and sensitivity (maximizing new candidate interactions). This is not unexpected as the composition and stability of protein complexes varies between proteins. Overall, scoring thresholds between 0.85 and 0.95 achieved this balance. We have carefully clarified the selection of SAINT filters in the results section on page 10.

Was the expression level in the negative control samples (and the localization of the expressed protein) comparable to that of the baits analysed? From the Figure 1E, it does not appear so (meaning that the GFP is detected at much lower levels than the other baits), and this can drastically affect the recovery of the contaminant (sticky) proteins. Since there is very little here in terms of validation of the new

interactions, this is an important concern here. Furthermore, the total spectral counts across the three GFP replicates shown in Supplementary indicates a lot of variability in recovery, with sample 3 having twice as many spectra as sample 1. Since the software the authors chose to use (SAINT, and they are seemingly running each bait separately across the controls) depends critically of the negative controls, a major concern is that if these controls do not properly model the background, the conclusions are invalid. Have the authors tried to perform the same analysis but including many more controls and perhaps selecting the controls based on the total spectral counts in their negative control purifications? How stable is their interactome under these conditions?

> From Figure 1E, since the gel lanes were stained by Coomassie, it is difficult to use the degree of staining between different proteins, i.e. PYHIN-GFP and free GFP, to compare relative amounts. To address the reviewer's concern, we assessed the levels of GFP expression in whole cell lysates from the different generated cell lines (PYHIN-GFP or GFP alone). Our anti-GFP Western blot analysis, shown in supplementary Figure S2B, demonstrates similar levels of GFP.

> To further address the reviewer's concern, we now investigated the stability of our interactome. While the proteomic AP-MS workflows were identical, the biological control replicates (and also different PYHIN proteins) were performed by separate individuals at different times. We considered this an important advantage, as it captures the expected variability of AP-MS experiments. However, to further address the reviewer's concern, we have now performed a separate SAINT analysis, supplementing the analysis with three additional controls obtained from the Crapome repository (www.crapome.org). The Crapome is a database of negative control IPs performed in different laboratories using various AP-MS conditions. The controls were selected to match the cell type, the tag, and the instrument used for analysis. However, these controls were, of course, performed by different individuals and some of the experimental conditions varied (e.g., lysis buffers were less stringent than in our current study). After repeating the SAINT analysis using all six negative controls versus each PYHIN protein, the interactome in Figure 3 has been re-visualized in supplementary Figure S9 to indicate the overall stability of the interactions. From the 345 proteins within the HIN200 interactome, over 80% were retained after re-analysis using these three additional negative controls. Only 60 interactions no longer passed the SAINT scoring thresholds. Moreover, this loss is not surprisingly since the added controls had an overall higher background, likely due to their lower buffer stringency. Therefore, based on this result, we feel the background distribution in our paired negative controls recapitulates the variation present within the PYHIN-GFP isolations.

It is also not clear the authors requirement of having a SAINT score {greater than or equal to} 0.85 (or 0.95) in the AP-MS results with both N- and C-terminal GFP tag to be considered "specific" since the immunofluorescence data presented in Figure 1C does suggest different behaviors when the tag location is changed. This should probably be commented on.

> Given the limited prior knowledge of PYHIN protein interactions, we opted to be stringent in our analyses of interactions and focus only on the most prominent associations. Furthermore, we chose to analyze the N- and C-termini tagged proteins in an equivalent manner to avoid arbitrarily imposing a bias. As we have indicated in our manuscript, upon cell lysis, we do observe a slight loss of the baits for all C-terminally tagged proteins in the insoluble pellet (Figure 1F and page 10). It is possible that an even more stringent lysis buffer composition may further improve the extraction. However, from our experience, this is usually accompanied by a loss of weaker or lower affinity interactions. As this is the first interactome for these proteins, we tried to obtain a balance between the isolation, the

maintenance of interactions, and the accumulation of nonspecific associations. We have clarified this on page 9.

At the minimum, the authors should revisit their statistical analysis and generate suppl. table 3-5 anew and use this opportunity to improve their table naming scheme (the "specific common", "uncommon" and "non-specific uncommon" labels are really confusing and not widely use in the field).

> We agree with the reviewer that our original nomenclature was confusing. We have now rectified this and have reworded all the titles of our supplementary tables S3-S5, defining them as “SAINT-filtered Interactions Observed in Both N- and C-terminal Isolations for at least one PYHIN Protein (Fig 3)”, “SAINT-filtered Interactions Unique to Either N- or C-terminal Isolations (Excluded from PYHIN Family Interactome, Fig 3)”, and “Non-specific Interactions Excluded from the PYHIN Family Interactome”, respectively. Additional clarifying statements have been added to the methods sections.

5) Additional controls and/or experimental parameters would be helpful/essential in a few of the experiments. Examples include:
- Fig 1E - GFP blots of induced cell lysates to show how consistent the degree of induction/expression is across the different baits would be beneficial.
> As suggested by the reviewer, we have performed this experiment and have added a new figure (supplementary Figure S2B) showing similar induction levels for most PYHIN proteins, as well as for the GFP control. We have also added relevant discussion regarding the relative expression levels on page 8.

- In Figure 1F, the blots presented should be continuous to enable relative comparison between fractions (which is not the case for MNDA and AIM2). In addition, the relative amount loaded in each lane should be shown in the panel or in the Figure legend.
> We agree with the reviewer that it is important to show the continuous blots to facilitate comparison. As the reviewer has indicated, in figure 1F, we have included continuous blots for IFI16 and IFIX and trimmed versions for MNDA and AIM2. We selected to show the trimmed version to maintain the same order of the pellet (P), flow through (FT) and isolated (IP) fractions and facilitate the comparison for the reader. However, we have also included the full, uncropped blots in the supplementary figure S3A. These blots were exposed simultaneously. Should the reviewer want to see the raw blots, we would be happy to provide them. The bands represent 1% loading of each fraction. We have now clarified this loading in the supplementary figure legend, as well as in the legend of the main figure 1.

- Fig 4C - removal and/or mutation of the NLS would be a nice control to show that it specifically directs nuclear localization. The use of an NLS mutated GFP-IFIX-HIN200 construct could then demonstrate whether Ku80 can only bind to IFIX when it is in the nucleus.
> As mentioned above, given the addition of numerous new figures and tables, we had to remove some of our previous results to facilitate a more focused manuscript. As this is the first paper that investigates IFIX in immune response, we felt that it is more important to establish its role as a DNA sensor. Therefore, we have performed new experiments to further demonstrate its antiviral properties and its binding to foreign dsDNA (new Figures 5C-e and 6B-C). We believe that this revised manuscript provides a better and more focused representation of the main conclusions.

- In Figure 4D, there is an excess of Ku80 in the GFP-IFIX-HIN200 sample lane. As this sample is the one showing the positive results, the authors should rerun/repeat the experiments to obtain equal loading and strengthen their data. Furthermore, I would like to see an extra control sample where ethidium bromide is added to the samples to ensure that the interaction is not DNA dependent (see PMID:

1495986 for details). In this co-IP both bait and prey are known to associate with DNA so the DNaseI added in the buffer during lysis may not be enough to completely prevent DNA dependent protein interactions.

> We agree with the reviewer that the association with Ku proteins is challenging to study, given their well-known DNA-binding properties, and we appreciate the suggestion. We fully appreciate this important concept, as most of the projects in our lab focus on characterizing DNA-binding proteins. Given the overall comments from the editor and the reviewers to focus the manuscript, we have removed the results and the discussion of the interactions with the Ku proteins. This allowed us to focus on the interaction between PML and IFIX and to characterize their antiviral properties, providing a better transition between the interaction and the DNA sensing parts of our study. However, we want to take this opportunity to mention that, while all of our original immunoaffinity purification analyses have been done following treatment with DNase, the new isolations that we have included for IFI16 in fibroblasts and THP1 cells and for IFIX following HSV-1 infection were all done in the presence of benzonase, which digests both RNA and DNA. We observed Ku80 to bind to IFI16 in 293 cells, as well as in nuclei from differentiated monocytes (new Figure 4F).

- Fig 5C - I would like to see that induction of an "irrelevant gene" such as GFP alone does not induce IFN expression in the presence of VACN dsDNA transfection. Furthermore, this experiment has good dynamic range, and therefore would be a particularly useful opportunity to test the various sequence/domain/structural features of IFIX with an actual functional readout. For example, testing the necessity of the NLS and the NES for IFN induction would be particularly informative.

> We have now included the suggested additional controls (new Figure 6E). These results support our initial conclusions. We appreciate the suggestions regarding the NLS/NES, and consider these a starting point for a different follow-up study.

- Fig 5C and E - a nice control would be to use AIM2 in these experiments, since it lacks an apparent NLS and localizes primary to the cytoplasm it might be predicted that it should therefore be unable to detect HSV-1 dsDNA but might still be able to detect the VACV transfected DNA.

> We would like to clarify that AIM2 has not been reported to induce interferon expression in response to dsDNA. Therefore, it cannot serve as a control. AIM2 is known to function in inflammasome activity, which is not the focus of our study. We have revised the introduction to clarify these aspects.

- In Figure 5E, the WB needs to be displayed properly with both GFP and GFP-IFIX appearing on a single blot with molecular weight marker displayed properly. In its current format, the blot appears VERY suspicious.

> We have now included the full, uncropped western blot in current Figure 6F, complete with molecular weight markers. As shown in this panel, despite isolating less GFP-IFIX than control GFP, we still uniquely detect HSV-1 genomic DNA in the GFP-IFIX isolation and not in control GFP isolations.

6) In Figure 5B-D, the authors describe the role of IFIX and of its NES sequences in DNA sensing. To better characterize the function of IFIX here, the authors should test the capacity of their IFIX NES mutant in inducing IFN-beta expression in response to vaccinia virus DNA. One would expect that mutating the first NES motif would drastically reduce IFN-beta expression in response to VACV and thus, make a strong point for the authors' model.

> We agree with the reviewer that these experiments would be particularly interesting and informative. However, in response to general reviewer concerns and comments regarding the focus of our manuscript, we have removed the data related to the characterization of the IFIX NES structures. As

mentioned above, we have added new experiments that we feel further strengthen the main messages of our manuscript.

7) The lengthy description of the expression results for the PYHIN proteins in HEK293 cells is unnecessary; simply state that they are expressed, and importantly, that their expression level is induced by interferons.
> We agree and thank the reviewer for the suggestion. In response, we have now shortened and focused the data interpretation in this section (page 7).

8) Have the authors attempted to perform some of the GFP-MS experiments after treatment with nucleases such as RNAse and DNAse to estimate how much of the interactome is due to protein-nucleic acid rather than protein-protein interactions? Have they tried to test the effect of IFN-b on the interactome?
> All of our original immunoaffinity purification analyses have been done following treatment with DNase. We have now stated this more explicitly in the results section (page 9) and in the methods section. Furthermore, the new isolations that we have included for IFI16 in fibroblasts and THP1 cells and IFIX following HSV-1 infection were all done in the presence of benzonase, which not only digests both RNA and DNA, but also is more effective in a wider range of buffer conditions than DNase I. Regarding the second suggestion of performing these isolations following IFN-b treatment, we would like to respectfully point out that this is already an extensive study with 7 main figures and 12 supplementary figures, and already has significant novelty and biological impact. However, an interesting aspect to consider is the fact that overexpression of these PYHIN proteins may partly mimic their induction by IFN. This may explain why many IFIX interactions are shared between uninfected cells and cells infected with herpes simplex virus 1 (HSV-1) (new supplementary Figure S12). We have added discussion regarding this concept on page 23.

Minor points
1) Fig 1A and S1A - it might be helpful to state that/whether these are all normalized to GAPDH expression, as this information is only available in the methods.
> Thank you for the suggestion. In response we have added in the figure legends for all panels showing qPCR data a statement clarifying the endogenous control (*gapdh* or *β-actin*) used for mRNA normalization.

2) In the Supplementary Figures, the font choice and resolution seems particularly poor.
> Thank you for pointing this out. Originally the images were exported from Power Point at 96 dpi resolution by default. We have now exported them at 300 dpi and improved the quality of the images.

3) On page 9, the nature of the "protein-dependent cytotoxic effects" could be briefly mentioned.
> We have now clarified this on page 7, mentioning that "This transient overexpression avoided the potential PYHIN-mediated cell cycle arrest, apoptosis, and transcriptional changes that may be associated with this family of proteins" and have included the relevant citations.

4) I'm not sure the use of the word "pancellular" is appropriate - to me this implies "across all cell types". Something like "uniform distribution" might be more appropriate.
> As suggested, we have revised our language and replace "pancellular" with "uniform distribution"

5) Fig 1D - In the workflow, why is "PTM mapping" included for this particular study? Unless I have missed something, this is not at all relevant.

> Thank you for noticing this. We have now revised this figure to remove the PTM mapping.

6) For Figure 2B, I suggest that the authors expand their legend to describe the constituent of the main clusters (color bars) either by GO terms and/or protein complexes present in them.
> We have now included in the Figure 2 legend a description of each colored cluster with regards to which PYHIN protein(s) they were determined to specifically interact.

7) In Figure 3, the authors should explore using the edge thickness to display spectral count information and/or the nodes outline for SAINT score (the better the thicker for instance).
> We attempted to include these suggested visual enhancements to Figure 3, however, after overlaying the indicated spectral count and SAINT score data, the figure became very challenging to interpret. And in particular there were too many overlapping edges from the four PYHIN proteins to readily differentiate edge thicknesses, and similarly the differences between the lowest (0.85) and highest (1.0) SAINT scores could not be easily distinguished through node thickness. Therefore, as an alternative visualization we have included in Supplementary Figures S5-8, separate networks for each PYHIN protein, that were extracted from Figure 3 while maintaining the same layout for ease of comparison. Additionally, each node color now represents the relative enrichment of each co-isolated interaction (calculated by a normalized spectral abundance factor) versus its cellular abundance (obtained from the PAX database). These figures provide a richer depiction of the associated mass spectrometry spectral count data while maintaining a pleasant visual style.

8) The resolution of immunofluorescence images shown in Figure 4B and 4C is too low.
> We agree and apologize for the initial problems with the conversion of our figures. We have now corrected this.

9) In Figure 5D, the authors seem to have forgotten to show the localization of the WT IFIX construct. While this was shown in Figure 1C, it would be easier for readers if it was also present in this panel.
> The NES results were now removed, as mentioned above. For the current figure 6D, we have now included the localization of IFIX without transfection of VACV.

Reviewer #3:
This review, as requested, comments only on the proteomics methodology and data analysis.

The authors have used a reasonably standard affinity-purification mass-spectrometry (AP-MS) strategy using GFP tags to explore the interactome of the PYHIN family of proteins.Both N- and C- terminal recombinant tagged pbait proteins have been used (and for which good agreement was seen) and in sum have identified ~350 PYHIN interacting proteins, as gauged using statistical techniques. The proteomics analysis is thorough and well-done; the authors have verified the expression patterns of the recombinant bait proteins as well as using a cell line that apparently expresses the endogenous PYHIN proteins. Data analysis is also quite adequate. A few clarifications would help the reader:

The authors mention comparison of identified proteins to control AP-MS experiments of their own (3 expts) as well as to the CRAPome resource for filtering AP-MS contaminants. They also use the Saint program to score each bait-prey pair. However it may help the reader to know how reproducible the prey hits were. This reviewer understands that at least 2 reps were performed for each bait - for how many preys were identifications made in both replicate AP-MS experiments?

> This is an important point that we did not state clearly in the original manuscript. An important aspect of identifying candidate interactions using SAINT was our use of the average SAINT score between replicate AP-MS experiments replicates. By definition, a SAINT score threshold > 0.50 would ensure that all candidate interactions were detected in both biological replicates. Therefore, using our more stringent threshold (>0.85), we focused on candidate interactions that were present in both replicates. We have added these clarifying sentences to the Results (page 10) and the corresponding Methods section. Alternatively, if the reviewer is asking about identification reproducibility independent of SAINT analysis, then on average between biological replicates of the four HIN200 proteins, 80 +/- 10% of prey proteins were identified in both biological replicates. AS stated above, the proteins that were not present in both replicates were filtered out by SAINT.

Also if a prey was identified in 1 or more control AP-MS - were these automatically excluded from further analysis?

> No, we did not manually exclude proteins based on presence in the control because this does not account for the possibility for relative enrichment between control and bait AP experiments. Instead, we leveraged SAINT's ability to perform a non-biased and flexible analysis of non-specific spectral count distributions between different AP-MS experiments. Therefore, SAINT scores inherently account for both magnitude and reproducibility of contaminant protein detection in control APs.

Editorial Decision 01 December 2014

Thank you again for submitting your work to Molecular Systems Biology. We have now finally heard back from the two referees who accepted to evaluate the revised study. As you will see, the referees are now supportive. They raise however a series of points, which we would ask you to carefully address in a minor revision of the present work. The issues raised by reviewer #2 refer to the need of further clarifications. No additional data are required.

Reviewer #1:

The authors have addressed most of my major criticisms and presented a significantly revised version of their manuscript. In particular, the major conclusions are now supported by experimental data.

While I still believe that this manuscript is outside of the normal scope of MSB (and more appropriate for an immunology journal), the manuscript is of a technical high quality and brings interesting novelty.

Reviewer #2:

This paper has been substantially altered since the last version, with some changes requested by reviewers and some changes the authors' initiative. Some important controls and improved analysis has been introduced. Overall it represents a large amount of work. New work has been introduced using other cell types that confirms a majority of the interactions with IFI16. The proteomics work will provide a useful resource for ongoing work on PYHIN proteins. As noted by reviewer 2, the second part of the paper is like a separate paper, but defines IFIX as a protein binding foreign DNA, and having a contribution to induction of type I interferon.

Receptors involved in the induction of interferon by foreign DNA have been contentious for a number of years. cGAS has now clearly emerged as the essential receptor, with an established mechanism linking it directly to STING. The PYHIN proteins recognise foreign DNA, but it may be that they just modify the cGAS response, rather than directly signalling to STING. I think that the discussion of this paper needs to mention the clear data linking cGAS and STING with this process and acknowledge that how PYHIN proteins fit into this central signalling pathway is unclear. Of note, none of the proteins interacting with IFIX and IFI16 shed any light on a pathway that would lead to interferon synthesis. I suspect their promotion of interferon synthesis is an indirect effect rather than a direct signalling role.

The authors have done a significant amount of extra work, and I do not seek more, but would like clarification on data they probably already have:

Specific Comments
1. One of the major comments I had on the first version of the paper was that the level of expression of PYHIN genes in HEK293, was obscured in the paper. All real time PCR data was expressed relative to one sample, and no western blots were performed (I understand that IFI16 antibody is the only one you have that works, but even this is not used on HEK293 to show endogenous protein or lack thereof. I presume the authors have looked on a western blot with this antibody). I want to make it clear that I do not think it is a requirement for publishing this work that HEKs should normally express PYHIN proteins. I only want a clear description of what they express, and at what level. My concerns about presentation of real time PCR data in the previous version were justified by the statements of both other reviewers that showed they thought your data showed relevant levels of PYHIN expression in HEK293. Available online data (GNF) suggests negligible expression of these factors in HEK293. In fact it would not help your case if HEK293 expressed IFIX, because

then (on the basis of results presented in this paper) they would be expected to be responsive to transfected DNA without introduction of IFIX.

The paper has improved in that it has moved away from directly claiming biologically relevant levels of PYHIN proteins in HEK293. However, the figure on induction of the genes by interferon still obscures their level of expression, and the authors have not made any modification to display of expression data. I do not agree that using a delta delta Ct procedure for processing real time PCR data is appropriate. As I said before, it obscures the real quantification of individual mRNAs. The fact that this method is widely used does not sway me- it is in fact widely misused. I do realise that many prominent researchers and papers use this. But, why would you present levels of mRNA relative to one arbritary sample, when you have the option to present something that has more information content? If PCR is done with good efficiencies of amplification and I see real time PCR of a gene relative to hprt with a level of 0.001, I know that this is a rare mRNA in that cell. When I see a value of 10, I know from experience that this gene is highly expressed. I appreciate that sometimes normalisation helps presentation of data when there is variability in basal expression between experiments (which can seem large variation when expression is very low). There are two options to give more meaningful data. Firstly to present delta Ct results as I suggested previously. Or, alternatively to leave the data as it is, and state in the figure legend something like "the basal level of IFI16 expression relative to b-actin was 0.001" (i.e. the results for the control sample by delta Ct analysis). This affects figure 5E, 6E and S1A.

2. New Figure 4F and description in text - 40 IFI16 interacting proteins were found only in 293 cells. Were there any proteins found uniquely in THP1 and HFF, and if so what were they?

3. New Figure 5B - This colocalisation with PML and other proteins uses IFIX-GFP which spontaneously forms puncta (Fig 1C). The colocalisation with PML is very good, but what is the situation with N terminally tagged IFIX that does not form these puncta - how is PML localised in that case, is it diffuse? Is PML always punctate and could it recruit IFIX? Or is it the other way round, and the generation of IFIX puncta induce recruitment of PML?

4. New Figure 6G - Once again, the localisation of IFIX in response to viral infection is done with the IFIX-GFP which already forms puncta without virus, rather than the N terminally tagged version. Did you look at both? Does the viral infection induce puncta in the N terminally tagged version?

5. Please mention cGAS in your discussion, as the receptor shown to be essential for DNA-dependent interferon induction, in every system where it has been studied.

Minor points
1. Results paragraph 1. The authors have introduced an argument that lacks logic in saying that HEK cells are good for looking at interferon responses but not AIM2 responses. I realise this is in response to my comment, but it rather misses the mark. HEKs normally respond to DNA with neither inflammasome nor interferon responses as they are missing components of both pathways. They are useful in reconstitution experiments for looking at both pathways. The section... "Furthermore, induction of interferon response was reported.......provide a useful system for studying interferon response" should just be substituted with something like "HEK293 cells lack most innate immune signalling pathways but are useful in reconstitution experiments where specific pathway components are reintroduced." OR "HEK293 cells do not normally respond to transfected DNA with type I interferon induction, but this pathway can be reconstituted by introduction of DNA receptor proteins." Also, you show a western blot for STING in these cells, without indicating molecular weight. It would be worth one sentence to acknowledge that experiments in HEK293T cells have shown a lack of STING, and a need for its reintroduction to allow DNA responses (in your reference list - Sun et al. 2013). So is there a difference with 293Ts, or are your HEK293 cells unusual in expressing this?

2. Results last paragraph - "UL39 may represent" should say "UL39-IFIX interaction may represent"...

3. Some of the writing and assertions seem a bit "loose". I can't see the justification for the statement in the introduction that "Considering their substantiated roles in inflammatory and immune

processes, the PYHIN proteins are thought to antagonize the development of autoimmune disorders, including SLE and rheumatoid arthritis." Whilst it has been hypothesised that AIM2 might antagonise SLE development, I cannot see the cited papers relate to this idea, and nor does it flow from the first part of the sentence that an involvement in inflammatory and immune processes would make a protein into an antagonist of autoimmunity.

4. Results says that IFI16 had partial nucleolar staining. The figure seems to show exclusion from the nucleoli.

5. Discussion ..."Members of the 5FMC complex were enriched during infection relative to uninfected cells, suggesting IFIX is co-opted or reprogrammed by HSV-1 to regulate transcription." This interaction could equally well be a cellular attempt to combat the HSV gene transcription.

2nd Revision - authors' response 10 December 2014

(see next page)

Thank you for the careful review of our manuscript. We are delighted that the reviewers have appreciated the substantial amount of work put into this study and the novelty of our findings. In what follows, we present a point-by-point answer to the reviewers' comments. The reviewers' comments are shown in italics and our answer is marked with ">". Page and figure numbers in our answers refer to the revised manuscript.

Editor:

Thank you again for submitting your work to Molecular Systems Biology. We have now finally heard back from the two referees who accepted to evaluate the revised study. As you will see, the referees are now supportive. They raise however a series of points, which we would ask you to carefully address in a minor revision of the present work. The issues raised by reviewer #2 refer to the need of further clarifications. No additional data are required.

1. *please submit your MS data to PRIDE or an equivalent public database and the high quality protein-protein interactions to a database of the IMEX consortium (http://www.imexconsortium.org/). Please refer to the relevant accession numbers in a Data availability section at the end of Materials & Methods.*

> The Data availability section now references the deposited proteomics data: "The mass spectrometry proteomics data have been deposited to the ProteomeXchange Consortium via the PRIDE partner repository with the dataset identifier PXD001572 and 10.6019/PXD001572. The protein interactions have been submitted to the IMEx consortium through IntAct and assigned the identifier IM-23523." (page 43)

2. *please include the Cytoscape session file as Source Data for Figure 3. The .cys file can be zip-compressed (this 'protects' the .cys extension) and uploaded as dataset.*

> We have included with our resubmission a zip file containing the Cytoscape sessions files.

3. *three to four 'bullet points' highlighting the main findings of your study*

> We have now included this, as below:

- The interaction network of the human PYHIN proteins highlights their roles in transcriptional regulation, chromatin remodeling, and DNA damage response.
- IFIX interacts and co-localizes with components of nuclear PML bodies.
- IFIX acts as an antiviral factor, limiting the replication of herpes simplex virus 1 (HSV-1).
- IFIX binds to viral dsDNA and contributes to the onset of innate immunity.

4. *a short 'blurb' text summarizing in two sentences the study (max. 250 characters)*

> We have now included this, as below:
" This study presents the global protein interactome of the human PYHIN proteins (AIM2, IFI16, IFIX, and MNDA) and defines IFIX as an antiviral factor and sensor of viral DNA."

5. *the source file (Adobe Illustrator. PowePoint?) of Figure 7 so that we can rework it into a synopsis image.*

> Figure 7 is now provided as a Powerpoint file.

6. *Please include an author contributions statement after the Acknowledgements section (see http://msb.embopress.org/authorguide)*

> This is already included in our manuscript.

Reviewer #1:

The authors have addressed most of my major criticisms and presented a significantly revised version of their manuscript. In particular, the major conclusions are now supported by experimental data.
While I still believe that this manuscript is outside of the normal scope of MSB (and more appropriate for an immunology journal), the manuscript is of a technical high quality and brings interesting novelty.

> We greatly appreciate the reviewer's positive comments and recognition of our work's novelty.

Reviewer #2:

This paper has been substantially altered since the last version, with some changes requested by reviewers and some changes the authors' initiative. Some important controls and improved analysis has been introduced. Overall it represents a large amount of work. New work has been introduced using other cell types that confirms a majority of the interactions with IFI16. The proteomics work will provide a useful resource for ongoing work on PYHIN proteins. As noted by reviewer 2, the second part of the paper is like a separate paper, but defines IFIX as a protein binding foreign DNA, and having a contribution to induction of type I interferon.

Receptors involved in the induction of interferon by foreign DNA have been contentious for a number of years. cGAS has now clearly emerged as the essential receptor, with an established mechanism linking it directly to STING. The PYHIN proteins recognise foreign DNA, but it may be that they just modify the cGAS response, rather than directly signalling to STING. I think that the discussion of this paper needs to mention the clear data linking cGAS and STING with this process and acknowledge that how PYHIN proteins fit into this central signalling pathway is unclear. Of note, none of the proteins interacting with IFIX and IFI16 shed any light on a pathway that would lead to interferon synthesis. I suspect their promotion of interferon synthesis is an indirect effect rather than a direct signalling role.

> We have followed the reviewer's suggestion and we have now included a discussion regarding this on pages 29-30, as shown below:

“It is interesting to consider our findings in the context of other identified DNA sensors. In particular, the recently reported cyclic GMP-AMP synthase (cGAS) (Sun et al, 2013), which synthesizes the endogenous STING agonist cyclic di-nucleotide cGAMP in response to cytosolic DNA (Ablasser et al, 2013a; Wu et al, 2013), is an important component of the cellular immune signaling pathway. However, we and others have demonstrated that IFI16 (Li et al, 2012; Orzalli et al, 2012; Unterholzner et al, 2010), and now IFIX (in this study), also contribute to the expression of IFN-□ in response to foreign DNA. Considering that IFI16, like cGAS, requires STING for its putative immune signaling functions, IFI16 may operate within the same signaling pathway and directly or indirectly promote the function of cGAS, as recently suggested (Thompson et al, 2014). This may also be the case for IFIX. However, both IFIX (as shown in this study) and IFI16 directly bind to viral DNA with high affinity via their HIN200 domains. Therefore, one alternative hypothesis is that IFI16 and IFIX directly contribute to transcriptional activation of type I interferons in the nucleus. This hypothesis is supported by the interactions with chromatin remodeling complexes and transcriptional regulators that we elucidate in our current study. This PYHIN-mediated transcriptional regulation would seemingly function against viruses two-fold, as IFI16 was also previously shown to silence viral gene expression by restricting transcription from viral promoters during infection (Johnson et al, 2014; Orzalli et al, 2013). Overall, elucidating the possible independent or coordinated functions of cGAS, IFI16, and IFIX requires further study.”

The authors have done a significant amount of extra work, and I do not seek more, but would like clarification on data they probably already have:

Specific Comments

1. One of the major comments I had on the first version of the paper was that the level of expression of PYHIN genes in HEK293, was obscured in the paper. All real time PCR data was expressed relative to one sample, and no western blots were performed (I understand that IFI16 antibody is the only one you have that works, but even this is not used on HEK293 to show endogenous protein or lack thereof. I presume the authors have looked on a western blot with this antibody). I want to make it clear that I do not think it is a requirement for publishing this work that HEKs should normally express PYHIN proteins. I only want a clear description of what they express, and at what level. My concerns about presentation of real time PCR data in the previous version were justified by the statements of both other reviewers that showed they thought your data showed relevant levels of PYHIN expression in HEK293. Available online data (GNF) suggests negligible expression of these factors in HEK293. In fact it would not help your case if HEK293 expressed IFIX, because then (on the basis of results presented in this paper) they would be expected to be responsive to transfected DNA without introduction of IFIX.

The paper has improved in that it has moved away from directly claiming biologically relevant levels of PYHIN proteins in HEK293. However, the figure on induction of the genes by interferon still obscures their level of expression, and the authors have not made any modification to display of expression data. I do not agree that using a delta delta Ct procedure for processing real time PCR data is appropriate. As I said before, it obscures the real quantification of individual mRNAs. The fact that this method is widely used does not sway me- it is in fact widely misused. I do realise that many prominent researchers and papers use this. But, why would you

present levels of mRNA relative to one arbritary sample, when you have the option to present something that has more information content? If PCR is done with good efficiencies of amplification and I see real time PCR of a gene relative to hprt with a level of 0.001, I know that this is a rare mRNA in that cell. When I see a value of 10, I know from experience that this gene is highly expressed. I appreciate that sometimes normalisation helps presentation of data when there is variability in basal expression between experiments (which can seem large variation when expression is very low). There are two options to give more meaningful data. Firstly to present delta Ct results as I suggested previously. Or, alternatively to leave the data as it is, and state in the figure legend something like "the basal level of IFI16 expression relative to b-actin was 0.001" (i.e. the results for the control sample by delta Ct analysis). This affects figure 5E, 6E and S1A.

> We have followed the reviewer's suggestion and have added the information about the basal expression levels to all the figure legends mentioned by the reviewer, as shown below:

Fig. 5E – "The basal levels of *ifix* expression in shSCR HFF cells relative to *actin* was 1.8E-5, which corresponds to a raw Ct value of ~31."
Fig. 6E – "The basal expression levels in the IFIX inducible HEK293 cells for *ifn-β* (right bar at +Tet/-VACV70) and *ifix* (left bar at -Tet/-VAVC70) relative to *gapdh* were 2E-6 and 3E-6, respectively. This corresponds to raw Ct values of ~28 and 27 for *ifn-β* and *ifix*, respectively."
Fig. S1 – "The basal expression levels in wild type HEK293 cells for *ifi16, ifix, mnda, aim2*, and *mxA* relative to *gapdh* were 1.2E-6, 5.2E-4, 9.7E-5, 4.6E-7, and 2E-6, corresponding to raw Ct values of ~29, 20, 22, 30, and 28, respectively."

2. New Figure 4F and description in text - 40 IFI16 interacting proteins were found only in 293 cells. Were there any proteins found uniquely in THP1 and HFF, and if so what were they?

> We have already included the full lists of proteins identified in THP-1 and HFFs in our previous submission as supplementary tables S7 and S8. While these interactions were initially used as validation of our results in HEK293 cells (with ~70% of HEK293 interactions being conserved in at least one other cell type), these different interaction networks also bring interesting cell type-specific differences. We have now included a discussion regarding some interesting interactions on Pages 25-26, as shown below:

"Other IFI16-associated transcription factors included NKRF, CIRHI1A, and SLTM. While these interactions were confirmed in THP-1 and HFF cells, not all associations with transcriptional regulation functionality were shared between cell types. For example, components of the functionally-linked PBAF and WINAC ATP-dependent chromatin remodeling complexes (e.g. ARID1A, SMARCA2, SMARCA4, SMARCC1, PBRM1, BAZ1B) were uniquely enriched in IFI16 complexes isolated from THP-1 monocytes. These remodeling complexes (among others) have well-documented roles in chromatin regulation during 1,25(OH)2D3-mediated transcriptional repression, which proceeds through recruitment of the VDR–RXR Vitamin D receptor to Vitamin D receptor response elements (Haussler et al. 1997). This is an important process in differentiation within the myeloid cell lineage. Curiously, another uniquely prominent IFI16 association in THP-1 cells was MNDA, which interestingly is also linked with 1,25(OH)2D3 signaling to cause differentiation of monocytes (Gaczynski et al, 1990). Given the

genetic interaction of IFI16 and MNDA (both are located on human chromosome 1q) and similar expression pattern in the hematopoietic system, it is tempting to speculate some sort of cooperativity or convergence of activities involving Vitamin D receptor signaling. More broadly, these data support previous reports that PYHIN proteins can function in either transcriptional activation or repression (Chen et al, 2006; Johnstone et al, 1998; Johnstone & Trapani, 1999; Xie et al, 1998)."

3. New Figure 5B - This colocalisation with PML and other proteins uses IFIX-GFP which spontaneously forms puncta (Fig 1C). The colocalisation with PML is very good, but what is the situation with N terminally tagged IFIX that does not form these puncta - how is PML localised in that case, is it diffuse? Is PML always punctate and could it recruit IFIX? Or is it the other way round, and the generation of IFIX puncta induce recruitment of PML?

> PML is well established to act as a scaffold for the formation of nuclear bodies with punctate appearance (Lallemand-Breitenbach & de The, 2010). In agreement with this, in other studies in our lab, we observed a punctate localization for PML in many different types of human cells, which do not overexpress IFIX. Therefore, it is unlikely that IFIX puncta induces the recruitment of PML. Rather, our results suggest that IFIX is likely recruited, through mechanisms still to be determined, to PML-containing nuclear bodies. We have included this discussion on page 18.

4. New Figure 6G - Once again, the localisation of IFIX in response to viral infection is done with the IFIX-GFP which already forms puncta without virus, rather than the N terminally tagged version. Did you look at both? Does the viral infection induce puncta in the N terminally tagged version?

> We were interested in specifically characterizing the behavior of the C-terminus tagged IFIX as 1) we previously reported the formation of oligomerization of the pyrin domain (Cell Host Microbe 2013), which is impeded by its tagging at the N-terminus (Figure 1), 2) we show in this manuscript that the C-terminus tagged construct is able to induce IFN-beta response following transfection with VACV70mer, and 3) the overexpression of C-terminus tagges IFIX inhibited HSV-1 titers. As we already observe IFIX interacting with PML within nuclear puncta in uninfected cells, we do not expect that this punctate formation is specifically induced by infection. In fact, we think that the prior localization of IFIX to PML may provide the opportunity for these proteins to act cooperatively in antiviral response upon deposition of viral genome. We have now clarified this by including a sentence in the results section on page 23: "As IFIX interaction with PML within nuclear puncta is already observed in uninfected cells, we do not expect that punctate formation is specifically induced by infection."

5. Please mention cGAS in your discussion, as the receptor shown to be essential for DNA-dependent interferon induction, in every system where it has been studied.

> As mentioned above, we have included additional discussion of IFI16 and IFIX and their involvement in eliciting IFN-β response to foreign DNA in the context of cGAS studies (Page 29-30).

Minor points

1. Results paragraph 1. The authors have introduced an argument that lacks logic in saying that HEK cells are good for looking at interferon responses but not AIM2 responses. I realise this is in response to my comment, but it rather misses the mark. HEKs normally respond to DNA with neither inflammasome nor interferon responses as they are missing components of both pathways. They are useful in reconstitution experiments for looking at both pathways. The section... "Furthermore, induction of interferon response was reported.......provide a useful system for studying interferon response" should just be substituted with something like "HEK293 cells lack most innate immune signalling pathways but are useful in reconstitution experiments where specific pathway components are reintroduced." OR "HEK293 cells do not normally respond to transfected DNA with type I interferon induction, but this pathway can be reconstituted by introduction of DNA receptor proteins." Also, you show a western blot for STING in these cells, without indicating molecular weight. It would be worth one sentence to acknowledge that experiments in HEK293T cells have shown a lack of STING, and a need for its reintroduction to allow DNA responses (in your reference list - Sun et al. 2013). So is there a difference with 293Ts, or are your HEK293 cells unusual in expressing this?

> It is important to note that the inducible HEK293 cell system used in these studies are derived from HEK293 cells, not HEK293Ts. Our previous discussion did not properly explain this difference. Previous studies have confirmed the presence of STING in normal HEK293 cells (Ishikawa & Barber, 2008) and its absence in HEK293Ts (for instance Sun et al. 2013, as the reviewer mentioned). Moreover, the molecular weight of the sole band we observed by Western blot from lysates of the inducible HEK293 cells using our STING-specific antibody (IMG-6422A, IMGENEX) corresponds to the previously observed molecular weight of STING (≈ 37 kDa), and we have added the molecular weight markers to Figure S1B. Therefore, we believe that HEK293 cells do, in fact, possess some components of the minimal immune signaling pathway, but not the components of the AIM2 inflammasome pathway (importantly ASC). We have already demonstrated that the overexpression of IFI16 in this same cell system is enough to promote IFN response to foreign DNA (Li et al, 2012), likely because of STING's presence. As suggested by the reviewer, we have now specifically clarified that STING is absent in HEK293T cells, but detected in HEK293 cells on page 7, as shown below:

"Previous studies have used HEK293T cells to reconstitute immune signaling pathways by reintroducing specific pathway components, which are normally absent in these cells. (Ablasser et al, 2013a; Ablasser et al, 2014; Ablasser et al, 2013b; Diner et al, 2013; Sun et al, 2013). Therefore, we instead used a system derived from HEK293 cells, in which we could detect endogenous STING and IRF3 (Supplementary Figure S1B), two central components of DNA stimulated immune signaling pathway (Tanaka & Chen, 2012). This is in agreement with previous reports that HEK293 cells already express STING (Ishikawa & Barber, 2008) and do not require its reintroduction for virus-triggered interferon response (Li et al, 2012) as in the case of HEK293T cells."

If necessary, we can provide copies of previous discussion via e-mail with other immunologists, who are in this field and have observed the same expression of STING in HEK293 cells.

2. Results last paragraph - "UL39 may represent" should say "UL39-IFIX interaction may represent"...

> We have revised this sentence on page 24 as suggested by the reviewer.

3. Some of the writing and assertions seem a bit "loose". I can't see the justification for the statement in the introduction that "Considering their substantiated roles in inflammatory and immune processes, the PYHIN proteins are thought to antagonize the development of autoimmune disorders, including SLE and rheumatoid arthritis." Whilst it has been hypothesised that AIM2 might antagonise SLE development, I cannot see the cited papers relate to this idea, and nor does it flow from the first part of the sentence that an involvement in inflammatory and immune processes would make a protein into an antagonist of autoimmunity.

> We agree with the reviewer that the original statement did not clearly tie together the concepts that PYHIN proteins are involved in immune and inflammatory processes, and that it did not properly fit within that paragraph. For the sake of length and clarity, we have now removed this sentence. (page 5)

4. Results says that IFI16 had partial nucleolar staining. The figure seems to show exclusion from the nucleoli.

> We agree with the reviewer there is an inconsistency between the text on page 8 and Figure 1B, top row. For the purpose of clarity, we have removed this statement.

5. Discussion ..."Members of the 5FMC complex were enriched during infection relative to uninfected cells, suggesting IFIX is co-opted or reprogrammed by HSV-1 to regulate transcription." This interaction could equally well be a cellular attempt to combat the HSV gene transcription.

> This is an excellent point, particularly in light of our results that HSV-1 progeny titers are reduced in IFIX overexpressing cells (Figure 5C). In response, we have included discussion on page 29 as below:
"In fact, members of the 5FMC complex were enriched during infection relative to uninfected cells. As we see a significant decrease in HSV-1 progeny titers in the same cell line (Figure 5C), it is possible that the IFIX-5FMC interaction may act as an inhibitor of viral gene transcription. Specifically, the transcriptional regulator targeted by the 5FMC complex, ZNF148, binds the canonical GC box sequence prevalent within both eukaryotic and viral (e.g. HSV-1) gene promoter regions (Law et al, 1998; Merchant et al, 1996). Furthermore, ZNF148 is known to compete with transcription factor SP1 binding within GC box regions, subsequently inhibiting the transactivation of SP1 target genes (De Bustos et al, 2005). As SP1 is a known activator of HSV-1 gene expression (Kim & DeLuca, 2002), the IFIX-5FMC interaction may inhibit HSV-1 transcription through displacement of SP1. Alternatively, the IFIX-5FMC interaction may reflect the co-opting of IFIX by HSV-1 to regulate cellular transcription."
